# What should be discussed when considering an induction of labour? A UK-wide, multi-centre Delphi study to develop a core information set for induction of labour

**DOI:** 10.1136/bmjopen-2026-118024

**Published:** 2026-05-27

**Authors:** Eve Bunni, Carol Kingdon, Vicky Bradley, Alexandra Hunt, Amy Mahdi, Thomas Axcell, Ria Jagadish, Sophie Fox, Millie O’Dair, Charlotte Simms, Yee Tan Munn, Laura Bonnett, Benjamin Greenfield, Caroline Cunningham, Siobhan Holt, Christy Burden, Joanna Ficquet, Elena Otero-Romero, William Parry-Smith, Mairead Black, Abi Merriel, On behalf of the Options Collaborative Group, Deborah Lawlor

**Affiliations:** 1 Bristol Medical School, Bristol, UK; 2 University of Cambridge, Cambridge, UK; 3 Universtiy of Nottingham, Nottingham, UK; 4 University of Liverpool, Liverpool, UK; 5 University of Bristol, Bristol, UK; 6 Liverpool School of Tropical Medicine, LIverpool, UK; 7 North Bristol NHS Trust, Bristol, UK; 8 Liverpool Women's University Hospital, Liverpool, UK; 1 Department of Women's and Children’s Health, Institute of Life Course and Medical Sciences, University of Liverpool, Faculty of Health and Life Sciences, Liverpool, UK; 2 NIHR Applied Research Collaborative, North West Coast, Liverpool, UK; 3 Liverpool Women’s University Hospital, Liverpool, UK; 4 Health Data Science, Institute of Populaton Health, University of Liverpool, Liverpool, UK; 5 Box Hill Hospital, Eastern Health, Melbourne, Victoria, Australia; 6 Royal Liverpool University Hospital, Liverpool, UK; 7 Academic Women's Health Unit, University of Bristol Faculty of Health Sciences, Bristol, UK; 8 Royal United Hospitals Bath NHS Foundation Trust, Bath, UK; 9 Cambridge University Hospitals NHS Foundation Trust, Cambridge, UK; 10 Shrewsbury and Telford Hospital NHS Trust, Shrewsbury, UK; 11 Keele University, Keele, UK; 12 Aberdeen Centre for Women’s Health Research, University of Aberdeen, Aberdeen, UK; 13 Directorate of Women and Children, NHS Grampian, Aberdeen, UK

**Keywords:** OBSTETRICS, Surveys and Questionnaires, Decision Making

## Abstract

**Abstract:**

**Objective:**

To develop a core information set for induction of labour. Rates of induction of labour for childbirth are rising in many high-income countries. In England, a third of women have their labours induced. National guidelines recommend women receive information to make informed decisions about induction.

**Design:**

Two-stage consensus study using modified Delphi.

**Setting:**

UK.

**Participants:**

Pregnant people, parents and professionals.

**Outcomes:**

Stage 1: A long list of information points was identified through a systematic review of reviews, reviewing patient leaflets, qualitative interviews and a stakeholder survey, with ongoing patient, public and professional involvement. Stage 2: Think-aloud interviews were undertaken to refine the Delphi survey before a two-round modified Delphi process where participants voted on the importance of the information items. Pre-specified criteria were used to select items taken forward to a consensus meeting.

**Results:**

199 information points were identified through systematic review (110), patient information leaflets (162), qualitative interviews (58) and a survey (93). 46 unique information items entered the first Delphi round after four think-aloud interviews, 2 items were added following round 2. 368 people (310 parents/58 professionals) participated in round 1 and 177 people (154 parents/23 professionals) in round 2. 44 items met inclusion criteria; one item excluded, and three items were carried forward for consensus meeting discussion where 12 overarching information points were agreed on.

**Conclusions:**

This study has established a consensus-based core information set for induction of labour from a sample of the birthing population and staff providing their care. The resultant set has been populated with evidence in line with national guidelines. It can be used by women and clinicians as a standardised starting point from which to personalise discussions about birth.

**Trial registration number:**

COMET Initiative registration 2600: Developing a core information set for induction of labour.

**STRENGTHS AND LIMITATIONS OF THIS STUDY**

- Parent and professional involvement were central to the development of the set, meaning it encapsulates the core information vital for informed decision-making.
- The core information long-list was generated from systematic reviews, existing patient information leaflets, a national stakeholder survey and pre-existing qualitative interviews for comprehensiveness.
- Information points extracted from existing qualitative interviews reduced research waste and ensured the information needs of many more women were included than would have otherwise been feasible.
- The use of consensus methodology meant attrition in round 2 of the Delphi survey was relatively high, more so for professionals, but not unusual for this methodology and large sample size.
- The education status of the participants was high, despite use of social media and hospital-based recruitment, and the ethnic diversity of participants was slightly less than the national average of 16%, with 87% white British and 13% from other ethnicities, despite efforts to maximise inclusivity.

## Introduction

 Induction of labour refers to interventions used to stimulate the start of labour before its spontaneous onset.^1^ Worldwide, one in five births are induced, mainly in high-income countries, where use is rising.^2^ In the UK, one in three births are now induced, with maternity statistics from England showing an increase in rates from 22% in 2011/2012 to 30% in 2024/2025.^3^ Within this rise, there is a wide variation in rates between maternity care providers, ranging from 16–46%.^4^ The UK’s National Institute for Health and Care Excellence (NICE) recommends women are given comprehensive information about induction of labour and are involved in personalised discussions and decision-making about induction of labour, expectant management or planned caesarean birth.^5^

Women can opt for induction for maternal or fetal indications because they are post-dates or because they request or are offered it to reduce their risk of stillbirth.^6^ Some women report an increased feeling of control.^7^ Observational evidence has suggested that induction of labour may be associated with limitations on birth location, possible increased pain and instrumental birth with its associated increased risk of third or fourth-degree perineal tears.^5^ However, systematic review evidence of randomised controlled trials has not seen an associated increased risk with induction vs expectant management of labour.^8^ The benefits of induction of labour include: reducing risk of unplanned or emergency caesarean, admission to the neonatal intensive care unit (NICU), stillbirth in prolonged pregnancies and shortening the length of labour with use of oxytocin.^6 9^ Nevertheless, women often report that they do not receive enough information about induction and are unclear of the indication, risks, benefits or practicalities of the process.^10^

For women to make informed decisions about induction they need to be equipped with sufficient, evidence-based, consistent information about outcomes and experiences.^11^ Women’s experience of induction of labour is mixed, some studies describe induction as having little effect on satisfaction compared with spontaneous labour,^7^ while others found women were less satisfied when it came to respect, kindness and communication.^12^ Themes of lack of information and choice associated with induction have been repeatedly highlighted globally,^13^,^15^ suggesting a longstanding need for improved informed decision-making about induction of labour.^16 17^ A 2019 qualitative evidence review suggests provision of good quality, appropriately timed information and supporting women to be involved in decision-making will benefit women.^15 17^ A minimum standard of information provision for every woman considering an induction of labour could help fill this gap and improve agency across social and ethnic groups for greater equity in informed decision making. Core information sets offer a systematic approach to collating a minimum-set of information deemed important to women and those working in maternity care. Achieving consensus between groups is key to gathering information that can act as a foundation for clinical conversations.^18^

The aim of this study was to develop a core information set for women who are planning, considering or have been advised to have an induction of labour to support clinical discussions, decision-making and women’s choice.

## Methods

### Study design

**Figure 1:**
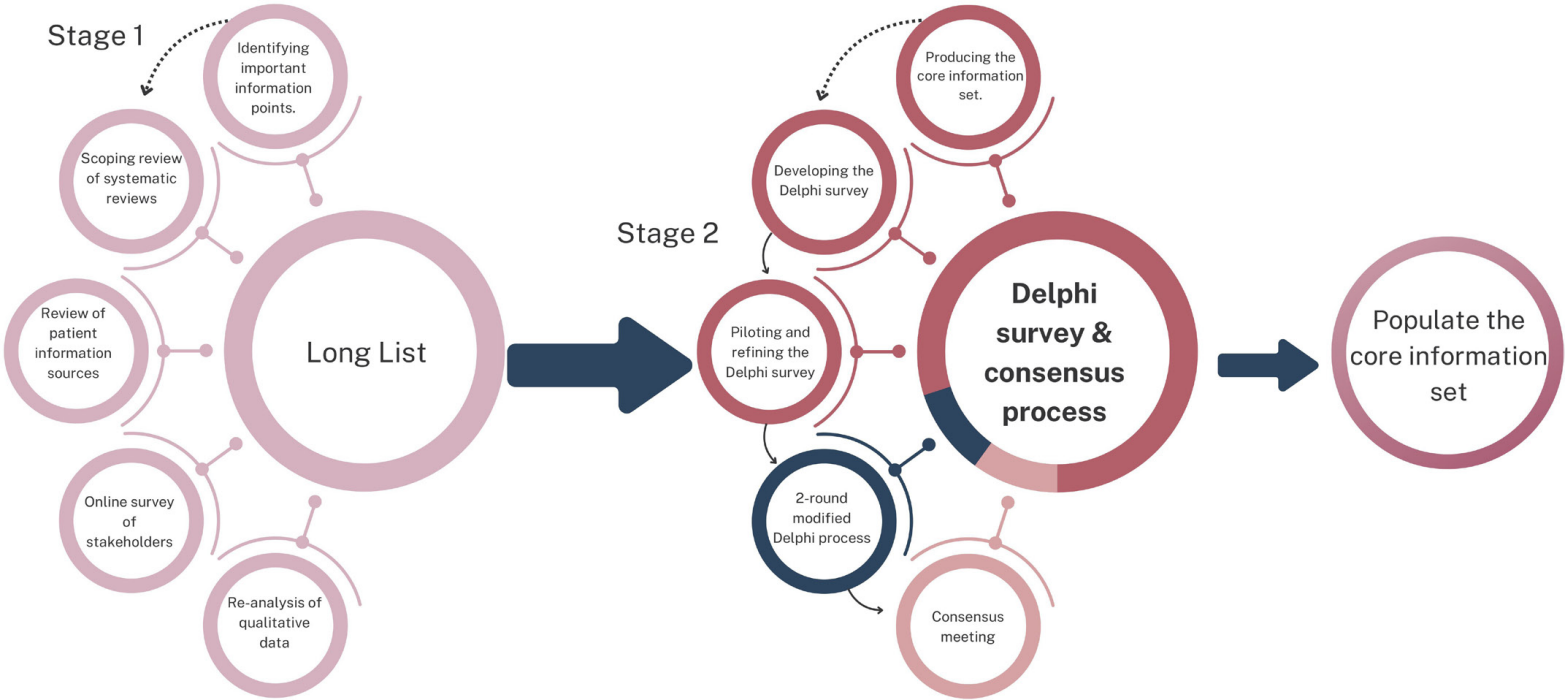
Overview of study design and process.

We conducted a two-stage consensus study using a modified Delphi approach (online supplemental file 1). Figure 1 provides an overview of the study design and process. The study was registered and follows the protocol and standards of the Core Outcome Measures in Effectiveness Trials (COMET) initiative.^19 20^ We adapted the Core Outcome Set-STAndards for Reporting purposes (COS-STAR). Online supplemental file 2 is the COS-STAR checklist.^21^ The induction of labour core information set is part of a programme of Birth Options research. We emulated our published vaginal birth core information set protocol.^22^ A favourable ethical opinion by health research authority for this study was granted on 6 April 2023 by the Southwest Central Research Ethics Committee (23/SW/0022). The research was conducted over a 2-year period between 2023 and 2025.

### Patient and public involvement

Patient and public involvement was embedded throughout the study. Patients contributed directly to discussions during the development of the long list and consensus meetings. Two members of the public sit on the study steering committee. In addition, four involvement groups were held to advise on the style, language and graphics used in the final information set. Three were held online with a total of 36 attendees and one was in-person. This was held in a community venue to encourage involvement of those who otherwise may not engage in research and was attended by 15 parents.

### Participants

Participants for the stakeholder survey, Delphi survey and consensus meetings included pregnant women, those planning pregnancy, recently given birth, healthcare professionals working in maternity care, medico-legal professionals or representatives from groups who have an interest in women’s birthing rights. The groups were split into ‘professionals’ and ‘parents’. Those who indicated that they either worked in maternity care, were a medico-legal professional or from a representative group were categorised primarily into the ‘professional’ group. Professionals were also able to state whether they were a parent and data for this has been provided in table 2. There is no general agreement on the sample size for Delphi surveys. Based on previous research and the specific requirements of this study, we sought to engage at least 100 participants for the Delphi rounds and approximately 20 stakeholders for the consensus meetings.^23^

### Stage 1: Development of the information long-list

#### Review of systematic reviews

We identified quantitative and qualitative systematic reviews to ascertain outcomes and information points reported in studies about induction of labour. Electronic databases were searched for English language systematic reviews published between 2018–2023 (online supplemental file 3). The time frame was specified to ensure up-to-date reviews were included and that the task was efficient. Records were screened in duplicate, full texts retrieved and data extracted for information points.

#### Review of patient information leaflets

An internet search was conducted to identify patient information leaflets. Information sources included NHS trust websites, international maternity organisations, for example, American Colleges of Obstetricians and Gynaecologists and non-NHS national sources, for example, Tommy’s, Royal College of Midwives and National Childbirth Trust (online supplemental file 4).^24^,^26^

#### Re-analysis of existing qualitative data

Three existing datasets were used to minimise research waste: A study about information wanted during antenatal education^27 28^ including focus groups involving women (46 participants) and staff (21 participants), along with individual interviews with women (9 participants); paired antenatal and postnatal interviews (22 interviews) and postnatal interviews (11 participants) along with focus groups of staff members (24 participants) discussing information needed to make decisions about birth; and 17 qualitative interviews about information about vaginal birth.^2229^,^31^ Transcripts were read and information points for induction of labour were extracted.

#### Stakeholder survey

A UK-based online stakeholder survey using REDCap^32^ asked participants to list key information points about induction (online supplemental file 5). Participants included pregnant women, those planning pregnancy, recently given birth, healthcare professionals working in maternity care, medico-legal professionals or representatives from groups who have an interest in women’s birthing rights. They were recruited online through social media and NHS hospitals. The results were thematically analysed to identify key information points.

### Stage 2: Delphi survey and consensus process

All the information points from stage 1 were collated then grouped into subcategories. Members of the research team, patient contributors and stakeholders then removed duplicates, collapsed similar points together and ensured they were in plain English.

A draft Delphi survey was uploaded onto REDCap^32^ and piloted with women over Microsoft Teams, during ‘think aloud’ interviews.^33^ Consent was obtained online prior to the interview. The survey was iteratively updated and agreed on by the team. The process ended when no further changes were made to the survey. The Delphi survey was then translated into Arabic and Polish, as two of the most commonly spoken languages after English. A copy is available in online supplemental file 6).

#### The Delphi survey

We recruited stakeholders to answer the survey including women aged 16 or over, who are antenatal, planning a pregnancy or recently had a baby or their partners; healthcare professionals who work alongside women in labour and postnatally (doctors, midwives, midwifery care assistants, physiotherapists); medico-legal experts who have an interest in obstetrics and representatives from groups who have an interest in women’s birthing rights. Recruitment was via social media (Instagram, Facebook and X), mailing list contacts, posters displayed in clinical areas at six NHS hospital sites and emails to relevant organisations. The Arabic and Polish posters were publicised online and disseminated during antenatal clinics for non-English speakers.

The survey was presented using a two-round modified Delphi process through REDCap.^34^ Each round stayed open for a minimum of 2 weeks to allow sufficient recruitment and follow-up. Demographics were collected including age, area of residence, ethnicity, level of education, parity and modes of birth. Participants were divided into the parent/non-professional group or professional group. Professionals could also identify as parents and if so, could answer the parent-related questions. Professionals were asked their job role, work location and years of experience. To avoid hoax respondents, an increasingly common problem with online survey research, CAPTCHA screening software was used at the beginning of each survey. CAPTCHA is a recognised method of protecting against bots.^35 36^

During round 1, participants rated each information item for inclusion in the core information set on a 9-point Likert scale (1–3 of limited importance, 4–6 important but not critical, 7–9 critically important). Items were excluded after round 1 and 2 if ≥80% of either stakeholder groups (parent/professional) scored the point from 1 to 3 (of limited importance), and <15% from either group scored the item from 7 to 9 (critically important). Participants had the opportunity to contribute further suggestions for information items.

Round 2 of the survey was distributed via the emails provided by the participants during round 1. Three reminders were sent out over the 8-week recruitment period. Information items retained were presented again, along with the participant’s score and the scores for all participants. Participants were asked to vote again using the same 9-point Likert scale described above.

If ≥80% of either stakeholder group scored an information item as critically important^7^,^9^ and <15% from either group scored it as of limited importance (1–3), the information item was carried forward for inclusion in the core information set. Any items that did not reach consensus were carried forward for discussion in the consensus meeting. Items meeting the above exclusion criteria during round 2 were removed from the core information set.

#### Consensus meeting

An online consensus meeting including parents and professionals was held using Microsoft Teams. Retained information points were presented to the attendees, who agreed they were important points. For non-consensus items, median scores for both rounds and histograms displaying the spread of scores across both groups were presented. The group discussed the information point and whether it was critical to include. Anonymous voting then took place using Poll-Everywhere software,^37^ with instant results presented back to the group. Consensus to include the item was defined as ≥80% of attendees voting to ‘include’ the item, exclusion was defined as ≥80% of attendees voting to ‘exclude’ the item. If no consensus was achieved, further discussions and revotes occurred until consensus was obtained. All information points were then discussed and refined until the final core information set was agreed.

#### Populating the induction of labour core information set

The information needed to communicate the final induction core information set was populated using a pre-defined hierarchy of sources including NICE Guidelines, Royal College of Obstetricians and Gynaecologists’ Green Top Guidelines, and systematic reviews giving priority to Cochrane Reviews. Once populated the information points were adapted to create a summarised version, parents and professionals met four times to refine the content and wording for the final populated set. A copy is available in online supplemental file 7.

### Analysis

Descriptive statistics were used to present the demographics of the Delphi survey participants. Pre-specified criteria were determined for exclusion and inclusion of the information points. Any free text responses during the first round of the Delphi process were extracted, collated and relevant new information items were added to round 2 for voting. Histograms and descriptive statistics were created for each domain for the consensus meeting.

## Results

### Stage 1: Development of the information long-list

The systematic review identified 110 information points from 98 papers (the Preferred Reporting Items for Systematic Reviews and Meta-Analyses (PRISMA) diagram depicting this process is online supplemental file 8); 162 information points were extracted from 72 leaflets; and re-analysis of 3 qualitative interviews identified 58 points. The stakeholder survey identified 93 information points from 173 participants. Combining these the initial long list contained 199 information points (online supplemental file 9). After adjustments, removal of duplicates and refinements, the information points were organised into 10 sections for the Delphi survey: Reasons for being offered induction, methods, process, risks and benefits, monitoring during induction, outcomes, pain management, practicalities, induction of labour and the NHS and decision-making about induction of labour. After piloting with four think-aloud interviews, 46 information items were included in the first round of the Delphi survey.

### Stage 2: Delphi survey and consensus process

#### The Delphi survey: round 1

The first round of the Delphi was completed by 368 people, of which 310 (84.2%) were parents and 58 (15.7%) were professionals. The parent group was largely made up of postnatal (238/310) or currently pregnant (107/310) parents; only 10 participants were partners. Participants most frequently had a bachelor’s or postgraduate degree (126 and 137 parents respectively). All but 81/310 participants were 31–40 years old. Participants were recruited from all regions of the UK; 87% parents were white British, 8% were White Other and 5% identified as other ethnicities. No participants completed the Arabic and Polish version of the Delphi surveys. For those that had previously given birth, 46% had experienced a vaginal birth, 24% had an emergency caesarean birth and 9% had an elective caesarean birth. Table 1 reports parent’s participant characteristics.

**Table 1:** Induction of labour core information sets

Characteristics	Parents	
	Round 1 (number and %) n=310*	Round 2 (number and %) n=154*
Roles (able to select more than one)†		
Planning pregnancy	16/371 (4.3%)	10/189 (5.3%)
Currently pregnant	107/371 (28.8%)	56/189 (29.6%)
Given birth in past	238/371 (64.2%)	118/189 (62.4%)
Partner of someone who is currently /previously pregnant	10/371 (2.7%)	5/189 (2.6%)
Person who is currently pregnant—gestation in weeks	27 (9)	25 (10)
Number of children	1.43 (0.80)	1.54 (0.88)
Length of time since most recent birth	2.50 (1.89)	2.77 (2.13)
Person who has previously given birth—type of birth experienced (able to select more than one)†		
Vaginal birth	145/313 (46.3%)	74/161 (46%)
Instrumental birth	65/313 (20.8%)	35/161 (21.7%)
Emergency caesarean birth	74/313 (23.6%)	36/161 (22.4%)
Elective caesarean birth	29/313 (9.3%)	16/161 (9.9%)
Gender		
Female	304/310 (98%)	154/154 (100%)
Male	4/310 (1.3%)	0/154 (0%)
Prefer not to say	2/310 (0.6%)	0/154 (0%)
Ethnicity		
Asian/Asian British	8/310 (2.6%)	4/154 (2.6%)
Mixed/multiple ethnic groups	6/310 (1.9%)	3/154 (1.9%)
Prefer not to say	2/310 (0.6%)	1/154 (0.6%)
White British	269/310 (87%)	132/154 (86%)
White Other	25/310 (8.1%)	14/154 (9.1%)
Age*‡*	34 (5)	34 (5)
Age categories		
21–30	61/310 (20%)	30/154 (19%)
31–40	229/310 (74%)	116/154 (75%)
41–50	19/310 (6.1%)	8/154 (5.2%)
Under 21	1/310 (0.3%)	0/154 (0%)
Highest level of education		
A-levels or equivalent	36/306 (12%)	16/150 (11%)
Bachelor’s degree or equivalent	126/306 (41%)	63/150 (42%)
Completion of secondary education to 16 years	3/306 (1.0%)	2/150 (1.3%)
Other	3/306 (1.0%)	2/150 (1.3%)
Post-graduate degree	137/306 (45%)	67/150 (45%)
Prefer not to say	1/306 (0.3%)	0/150 (0%)
Employment		
Employed full time/on maternity leave	186/306 (61%)	82/150 (55%)
Employed part time/on maternity leave	90/306 (29%)	51/150 (34%)
Homemaker	21/306 (6.9%)	13/150 (8.7%)
Not currently employed	4/306 (1.3%)	2/150 (1.3%)
Other	2/306 (0.7%)	0/150 (0%)
Student	3/306 (1.0%)	2/150 (1.3%)
Area of residence		
East of England	29/306 (9.5%)	9/150 (6.0%)
London	15/306 (4.9%)	7/150 (4.7%)
Midlands	34/306 (11%)	18/150 (12%)
North East England and Yorkshire	37/306 (12%)	19/150 (13%)
North West England	62/306 (20%)	29/150 (19%)
Northern Ireland	7/306 (2.3%)	2/150 (1.3%)
Scotland	17/306 (5.6%)	13/150 (8.7%)
South East England	47/306 (15%)	23/150 (15%)
South West England	44/306 (14%)	24/150 (16%)
Wales	13/306 (4.2%)	5/150 (3.3%)
Other	1/306 (0.3%)	1/150 (0.7%)

* n/N (%); mean (SD).

† Participants could select more than one category.

‡ Mean and SD for age were calculated using the midpoint from age categories.

The professional group was largely made up of midwives (24/58) and obstetricians (13/58). There were some midwifery care assistants, general practitioners (GPs), researchers, members of an interested charity or organisation, and ‘other’ professions working in maternity care including a doula, health visitor, midwife sonographer, a physiotherapist and a paediatrician. Table 2 reports professional participant characteristics.

**Table 2:** Induction of labour core information sets

Characteristics	Professionals	
	Round 1 (number and %) Professional n=29 Professional and parent n=29 n=58*	Round 2 (number and %) Professionals n=8 Professional and parent n=15 n=23*
Role (able to select more than one)†		
As a parent		
Planning pregnancy	3/38 (7.9%)	1/20 (5%)
Currently pregnant	10/38 (26.3%)	7/20 (35%)
Given birth in past	24/38 (63.2%)	11/20 (55%)
Partner of someone who is currently /previously pregnant	1/38 (2.6%)	1/20 (5%)
As a professional		
Midwife	24/60 (40%)	9/23 (39%)
Midwife assistant	5/60 (8.3%)	1/23 (4.3%)
Obstetrics and gynaecology doctor	13/60 (21.7%)	3/23 (13%)
Researcher	3/60 (5%)	1/23 (4.3%)
General practitioner	2/60 (3.3%)	1/23 (4.3%)
Physiotherapist	1/60 (1.7%)	1/23 (4.3%)
Member of charity organisation	1/60 (1.7%)	0/23 (0%)
Other	11/60 (18.3%)	7/23 (30%)
Person who is currently pregnant—gestation in weeks	23 (10)	28 (8)
Number of children	1.59 (0.69)	1.57 (0.76)
Length of time since most recent birth	4.33 (4.12)	3.57 (2.64)
Person who has previously given birth —type of birth experienced (able to select more than one)†		
Vaginal birth	18/28 (64.3%)	8/15 (53.3%)
Instrumental birth	4/28 (14.3%)	4/15 (26.7%)
Emergency caesarean birth	4/28 (14.3%)	3/15 (20%)
Elective caesarean birth	2/28 (7.1%)	0/15 (0%)
Gender		
Female	54/58 (93%)	21/23 (91%)
Male	4/58 (6.9%)	2/23 (8.7%)
Ethnicity		
Asian/Asian British	4/58 (6.9%)	1/23 (4.3%)
Black /African /Caribbean /Black British	2/58 (3.4%)	0/23 (0%)
Mixed/multiple ethnic groups	2/58 (3.4%)	0/23 (0%)
Other ethnic groups	2/58 (3.4%)	0/23 (0%)
White British	40/58 (69%)	19/23 (83%)
White Other	8/58 (14%)	3/23 (13%)
Age‡	37 (10)	38 (11)
Age categories		
21–30	13/58 (22%)	6/23 (26%)
31–40	30/58 (52%)	11/23 (48%)
41–50	7/58 (12%)	2/23 (8.7%)
51–60	7/58 (12%)	3/23 (13%)
61–70	1/58 (1.7%)	1/23 (4.3%)
Highest level of education		
Bachelor’s degree or equivalent	12/27 (44%)	6/14 (43%)
Other	2/27 (7.4%)	1/14 (7.1%)
Post-graduate degree	13/27 (48%)	7/14 (50%)
Employment		
Employed full time/on maternity leave	19/27 (70%)	10/14 (71%)
Employed part time/on maternity leave	6/27 (22%)	3/14 (21%)
Other	1/27 (3.7%)	1/14 (7.1%)
Student	1/27 (3.7%)	0/14 (0%)
Area of work		
East of England	11/58 (19%)	2/23 (8.7%)
London	6/58 (10%)	1/23 (4.3%)
Midlands	4/58 (6.9%)	2/23 (8.7%)
North East England and Yorkshire	2/58 (3.4%)	1/23 (4.3%)
North West England	18/58 (31%)	6/23 (26%)
Northern Ireland	1/58 (1.7%)	1/23 (4.3%)
Scotland	1/58 (1.7%)	1/23 (4.3%)
South East England	2/58 (3.4%)	0/23 (0%)
South West England	7/58 (12%)	6/23 (26%)
Wales	5/58 (8.6%)	2/23 (8.7%)
Unknown	1	1

* n/N (%); mean (SD).

† Participants could select more than one category.

‡ Mean and SD for age were calculated using the midpoint from age categories.

After the first round, no information items met the pre-defined criteria for exclusion (online supplemental file 10). Two additional information points were added by participants from round 1. The new items were possible delays during the process of induction and the likelihood of going into spontaneous labour depending on the number of completed weeks of pregnancy. Round 2 therefore included 48 information points.

#### The Delphi survey: round 2

Email addresses were provided by 365/368 participants from round 1. Of those who provided their email, 177 participated in the second round, giving a response rate of 48%–154 parents (87%) and 23 (13%) professionals. There was a higher dropout in the professionals group. 44 information items met the pre-defined criteria for inclusion and were presented in the consensus meeting for organisation and grouping. One information item met the pre-defined criteria to be removed from the information set*—the financial cost to the NHS of induction*. Three items did not meet criteria for inclusion or exclusion and were therefore carried forward to the consensus meeting (online supplemental file 11).

### Consensus meeting

**Figure 2:**
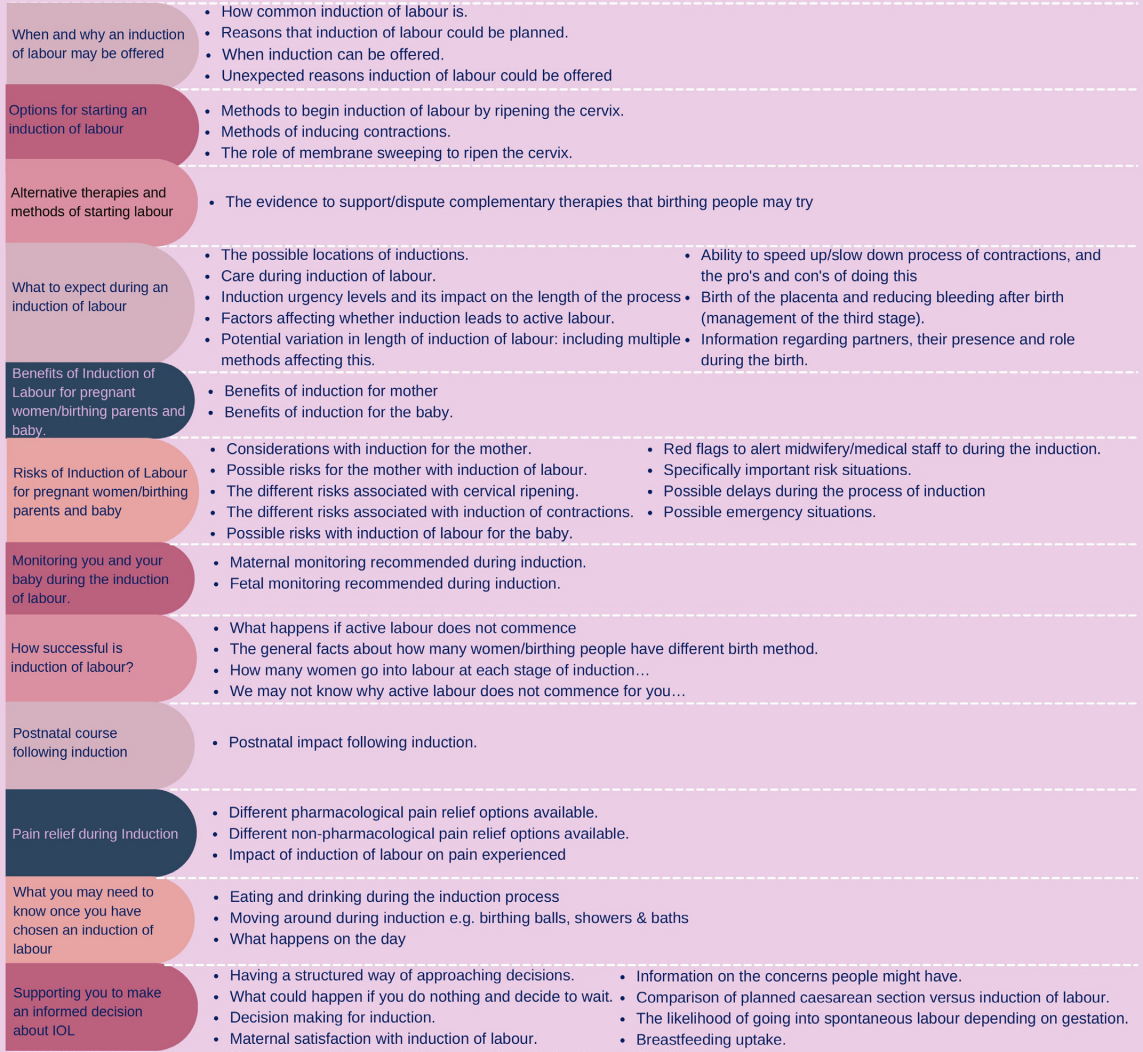
Induction of labour core information set.

25 participants attended the consensus meetings, both parents and professionals. After discussion, voting and re-voting, it was agreed that all three non-consensus items should be included, giving a total of 47 information items in the final induction of labour core information set. Discussions took place to generate ‘umbrella headings’ for the information items as some of the information points were closely related. This discussion began with the initial section headings in the Delphi survey and was developed from there. A final list of 12 overarching components was agreed which included 47 individual information items. These are shown in figure 2, induction of labour core information set.

### Populating the induction of labour core information set

Four engagement meetings were held, three online and one in-person, to refine the content of the core information set. Contributors included parents, midwives, doctors and website developers. Both a short and a longer version of the content for each element of the topic were discussed in the groups. The groups developed accessible wording and designed the flow of the information set. They also requested and outlined images/illustrations to support the communication of the information set.

## Discussion

This study has developed a core information set for induction of labour with parents and professionals to inform discussions and decision-making about induction of labour as recommended in clinical guidelines. The resultant set has 12 overarching components containing the 47 items, deemed to be ‘critically important’ information all women who are considering an induction (figure 2). It provides women and their partners with the information to support their decision-making alongside better equipping healthcare professionals working in maternity care to have a conversation incorporating the evidence important to women. The urgent need for standardised information was highlighted during the formation of the ‘long list’ of information points. While there was some repetition across the range of sources used, there was variability of information within each individual source, with some lacking data that others contained, highlighting a lack of consistency of information. This induction of labour core information set is one part of a series of information sets being developed with the aim of improving and standardising access to evidence-based information for women.^30 38^

Existing evidence shows that the information around choices during induction and what to expect is inadequate, which can contribute to anxiety around birth.^39^ Systematic reviews and qualitative studies report that when women are poorly prepared and informed about induction, they are more likely to have a poor experience, while staff also often feel underprepared to share information for decision-making.^17 29^ It is vital that women are equipped to make choices about birth. For this, they require high-quality accessible information. This is likely to be particularly important for the most vulnerable women. Engagement activities, including with women whose first language is not English or who are not university educated, have highlighted the importance of the short information set to reduce the amount of text to read.^40^ Therefore, an important element of communicating these facts is to ensure that information and data are shared in an understandable way. Women prioritised a friendly design and diagrams to support text and the use of icon arrays. We underpinned this with a survey study which found that while most diagrams are helpful, an icon array with 100 ‘pregnant women’ is likely to be the easiest to understand when communicating risk.^41^ This is in line with existing risk communication literature where best practice is presenting risks as frequency statements, absolute risks and natural frequencies over relative risks and conditional probabilities.^42^

A key strength of this study is its consensus methodology with parents and professionals, which strengthened its essential clinical content and relevance, ensuring it is well situated to support discussions about birthing options.^18^ However, despite our attempts to recruit a diverse sample of participants, we were not able to access a sample completely representative of the birthing population. 87% of participants were white British, 13% were of other ethnicities, falling below the UK average ethnic minority rate of 16%. 85% of parents had higher-level education, which is above the national reading age of 9–11.^43^ With this in mind, the core information set could be more set up for the groups described above. On the other hand, the majority of participants were 31–40 years old, capturing the most common age group to give birth in the UK, according to the most recent national maternity data where 53% of births occur between the age of 30–39, 36% are aged 20–29, with individuals at the extremes of age representing the smallest proportion.^3^

We adopted several strategies to increase the diversity of the participants. The Delphi surveys were online, and the involvement groups were held on Microsoft Teams, supplemented by in-person meetings in a local children’s centre in a socioeconomically disadvantaged area of Liverpool, where we had a majority of either non-English speaking or black and minority ethnic group women. We aimed to capture the opinions of stakeholders from all over the UK, as well as ensuring women who do not usually engage in research had their voices included, particularly while populating the core information set. The survey, study documents and materials calling attention of potential participants to the research were made available in English, Arabic and Polish, the most spoken languages in study sites, with local efforts made to recruit non-English speakers. Nevertheless, no non-English speaking participants attempted the survey. This highlights a gap in engagement; one possible approach in future would be to meet women face to face with interpreting facilities available. Methods of seeking participants through several social media platforms and local hospitals aimed to capture participants across all educational backgrounds but factors including internet access, the survey length, usability and the Delphi study itself may have influenced the educational level of the respondents.^44^

Professionals had the opportunity to indicate whether they were a parent. While this may increase the heterogeneity of this group, the professional status of the participant was considered their primary identity for the sake of the Delphi and feedback between rounds. That said, it was felt that the professional may wish to include their parent status as they may feel that it is part of their core identity.

Limitations of Delphi studies include difficulties with retention; attrition rates can vary from 0–92%.^23^ The drop-out rate between rounds was 52% across respondents. Initial observations reveal that if the group is split into parents and professionals, the dropout rate is 50% among parents and 60% among professionals. There is a higher dropout rate among professionals, highlighting a potential issue maintaining recruitment of professionals between Delphi rounds in comparison to parents. Furthermore, of the professionals that responded in the second round, 65% were also parents, while it was a 50/50 split in round 1. This may indicate that the group who feel most strongly about having this information available are parents and that professionals have multiple competing demands on their time.^45^

The general guidance to mitigate dropout in Delphi studies is to provide a short between-round time frame and send reminders out to participants.^46^ This Delphi had a 2-week between-round time frame, and three reminders were sent out to round 1 survey participants via email with the aim of maintaining interest and ownership. Although the response rate falls within the normal bounds of survey research, it is noteworthy that, in a Delphi study, the sample size and heterogeneity of the sample can affect response rates between rounds.^46 47^ Thus, in an effort to be inclusive, this study required a larger sample size, potentially contributing to the dropout rate.

The induction of labour core information set is intended to be immediately useful to clinicians tasked with delivering current clinical guideline recommendations for informed decision-making.^5^ This standardised information may help to address local practice patterns. The intention is not to oversimplify the complexity of decision-making about induction of labour.^48^ On the contrary, this induction of labour core information set is a response to the complexity of decision-making for women when faced with options that have consequences for mothers and babies. A 2025 survey of decision-making and knowledge around inductions of labour in Ireland reports 49% of women did not feel fully informed, 66% insufficiently informed and 30% did not know they could decline an induction.^49^ Age, parity and type of maternity care significantly influenced the decision process. This core information set goes some way to address the improvements they suggest are needed.

Further research is required. During involvement meetings, women suggested that local data are important for making birth decisions. However, that was beyond the scope of this study with its predefined hierarchy of evidence and commitment to use evidence underpinning NICE guidelines, systematic reviews and national statistics. That said, not all the evidence women wanted was available to populate the CIS. For example, while existing research shows induction of labour can reduce the likelihood of stillbirth, which disproportionately affects Black and Asian mothers, those with obesity and older women, there is no high quality, clear evidence to include information for women on the optimal timing of induction in these groups. The hierarchy of literature used by the research team highlights that guidelines contain mixed quality evidence within them. However, when populating the sets, women in our engagement groups felt that communicating the strength of evidence would overcomplicate the information. Instead, they favoured transparency by including references to the sources used. Future research should address questions important to women, for example the optimal timing of induction of labour, and find ways to engage women from all groups in information development and use.

### Conclusion

This study has established a core information set for induction of labour with a sample of the birthing population, their partners and healthcare professionals. This set acts as a standardised starting point from which clinicians can tailor information to support birth options. The set has been populated with the best available evidence in line with national guidelines. However, gaps in obstetric research evidence remain. Further work is required to disseminate and use the information in a clinical setting and engage women across diverse socio-economic and ethnic groups for personalised maternity care.

## Supplementary material

10.1136/bmjopen-2026-118024 online supplemental file 1

10.1136/bmjopen-2026-118024 online supplemental file 2

10.1136/bmjopen-2026-118024 online supplemental file 3

10.1136/bmjopen-2026-118024 online supplemental file 4

10.1136/bmjopen-2026-118024 online supplemental file 5

10.1136/bmjopen-2026-118024 online supplemental file 6

10.1136/bmjopen-2026-118024 online supplemental file 7

10.1136/bmjopen-2026-118024 online supplemental file 8

10.1136/bmjopen-2026-118024 online supplemental file 9

10.1136/bmjopen-2026-118024 online supplemental file 10

10.1136/bmjopen-2026-118024 online supplemental file 11

## Data Availability

Data are available upon reasonable request.
