# Supplementary material for: What should be discussed when considering an induction of labour? A UK-wide, multi-centre Delphi study to develop a core information set for induction of labour
Source: BMJ Open. 2026 May 27;16(5):e118024. doi: 10.1136/bmjopen-2026-118024 (PMC13218194; doi:10.1136/bmjopen-2026-118024)
Supplement: online supplemental file 1 [file bmjopen-16-5-s001.pdf]

Study Protocol

Development of Core Information Sets for induction of labour and caesarean section

LIST of CONTENTS

**GENERAL INFORMATION**	**Page No.**
TITLE PAGE	i
LIST OF CONTENTS	ii
KEY STUDY CONTACTS	iii
STUDY FLOW CHART	iv
SECTION	
1. BACKGROUND AND RATIONALE	1
2. RESEARCH QUESTION/AIM(S)	1
3. STUDY DESIGN/METHODS	2
4. STUDY SETTING	6
5. SAMPLE AND RECRUITMENT	6
6. ETHICAL AND REGULATORY COMPLIANCE	10
7. DISSEMINATION POLICY	12
8. REFERENCES	12
9. APPENDICES	13

KEY STUDY CONTACTS

Chief Investigators	Dr Abi Merriel<br><br>Centre for Women's Health Research, Institute of Life Course and Medical Sciences, Faculty of Health & Life Sciences, The University of Liverpool, Liverpool Women's Hospital, Crown Street, L8 7SS
Funder(s)	NIHR (Advanced Fellowship)
Key Protocol Contributors	Abi Merriel, Senior Clinical Lecturer, abi.merriel@liverpool.ac.uk<br><br>Andrew Demetri, NIHR Academic Clinical Fellow andrew.demetri@bristol.ac.uk<br><br>Danya Bakhbakhi, NIHR Academic Clinical Lecturer, danya.bakhbakhi@bristol.ac.uk<br><br>Anna Davies, Research Fellow, University of Bristol, anna.Davies@bristol.ac.uk

STUDY FLOW CHART

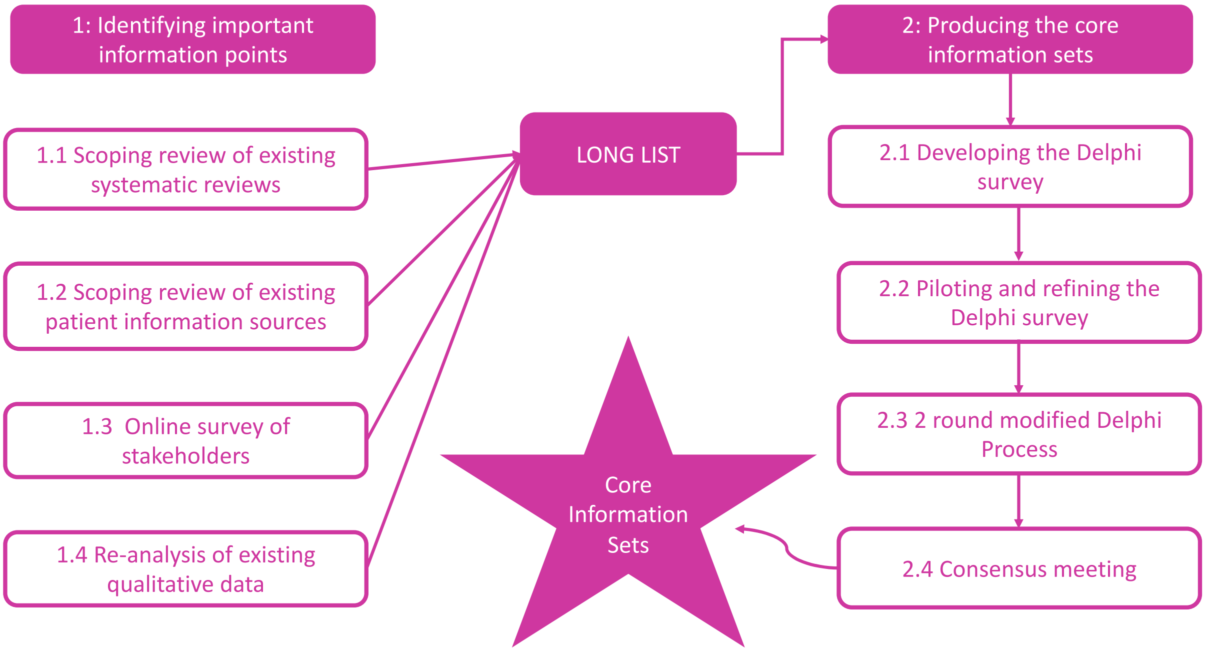

STUDY PROTOCOL

BACKGROUND

The development of these core information sets is part of a programme of work aimed at developing a tool called 'Options' to support women at high risk of emergency caesarean in their first pregnancies in making choices between planned spontaneous vaginal birth, planned caesarean section and induction of labour. The information sets developed will of course be of use to any woman considering these choices. A vaginal birth core information set is already in development (https://www.comet-initiative.org/Studies/Details/2069).

This protocol covers the development of two linked maternity core information sets

1) Induction of labour
2) Caesarean section

They are being included in the same protocol as the methodology is identical and the patient/stakeholder group we will invite to participate will be accessed in the same way. We plan to develop these two core information sets simultaneously as they will be required to feed into the 'Options' tool at the same time.

1 RATIONALE

Good clinical practice and guidance recommends that women have full choice and autonomy with regards to their labour. In order to facilitate this, it is vital that women are armed with key information points as to what decisions may lead to or entail. With regards to labour, it is important that women are provided with consistent information regarding different types of delivery. A lack of information prior to childbirth can contribute to poorer birth experiences by increasing anxieties with 'fear of the unknown' (Gammie & Key 2014, Lavender et al 2019). There has been work looking at identifying important outcomes surrounding caesarean section (Briscoe 2020) and induction of labour (Dos Santos 2018). But less has been done to understand what information women want to receive about caesarean section or induction of labour prior to deciding whether to proceed with these interventions.

In our ongoing Decision-Making during Childbirth qualitative study, the interviewed postnatal women have brought to light the lack, and inconsistency, of information they have received about both mode of birth (ie vaginal birth and caesarean) and induction of labour, despite shared decision-making being prioritised by NHS improvement (NHS England 2019). We believe it is important that women have access to consistent, accurate information, containing the facts that are important to them, when making decisions.

With this study, will develop a core information set for induction of labour and caesarean which will add to the vaginal birth core information set we are currently developing. This will guide the baseline

information which should be shared with women when discussing these interventions, and act as the foundation for further discussions on the topics that are important to each individual patient (Blazeby 2015, McNair 2019).

2 RESEARCH AIM

The aim of this research is to develop an understanding of what information is important for women to know about induction of labour and caesarean section.

2.1 Objectives

1. To develop a long list of information points women need to know about induction of labour and caesarean section.
2. To refine and carry out the Delphi surveys resulting in a final core information set.

2.2 Outcome

The outcome of this work will be two core information sets about induction of labour and caesarean section. Which can be used in synergy with the vaginal birth core information set currently being developed to guide the information provided to women about these processes/interventions around the time of birth.

3 STUDY DESIGN and METHODS of DATA COLLECTION AND DATA ANALYIS

There is no recognised single methodology for the development of a core information set. We will therefore use guidance from the COMET handbook for the development of core outcome sets (Williamson 2017) as the process is aligned to this methodology, and from existing core information set studies (Blazby 2015, McNair 2019)

Objective 1: Identifying information women need to know, developing the long list of information points.

1.1 <u>Scoping review of existing systematic reviews</u>

Aim: To identify information items and outcomes of interest to women about induction of labour and caesarean section.

Method: A pragmatic systematic search will be developed to identify outcomes reported in studies and key issues women identify about induction of labour and caesarean section. As we are looking for information points, we will focus on systematic reviews to ensure the number of records retrieved is manageable. We will identify systematic reviews of both quantitative and qualitative studies.

Records will be uploaded to systematic reviewing software (Covidence) and papers will be screened, the full texts retrieved, and the key information items extracted.

1.2 Scoping review of existing patient information leaflets

Aim: To identify information items provided in existing patient information sheets.

Method:
We will search the internet for sources of trusted patient information leaflets/articles/electronic information sources for example national and international medical/midwifery/nursing organisations (e.g. Royal Colleges), leaflets from heatlh providers (e.g. NHS or health systems in other countries) and from trusted non-governmental organisations (e.g. National Childbirth Trust). Information points will be extracted from these sources and added to the long-list from objective 1.1.

1.3 Online survey of key stakeholders

Aim: To explore the key stakeholder views on what information is important for women to receive about induction of labour and caesarean section.

Participants:
-Women/Birthing people, aged 18 or over, who are planning a pregnancy, antenatal or postnatal
- parents/birth partners of those planning pregnancy, currently pregnant or postnatal
-Healthcare professionals who work alongside women in labour and postnatally (doctors, midwives, midwifery care assistants, physiotherapists)
-Medico legal experts who have an interest in obstetrics
-Representatives from groups who have an interest in women's birthing rights (e.g. Birthrights, Maternity Action)

Method:
We will aim to engage approximately 100 survey participants, recruited online through social media (Appendix 1) , through posters/information/staff emails in NHS hospitals and in community spaces (Appendix 2). The survey will be carried out using REDCap software (Appendix 3). They will be asked to complete a consent form before starting (Appendix 4) and a link to the participant information sheet will be available within this consent text (Appendix 5). The survey will include information on the participants role and demographics, including if they have previously experienced an induction of labour or caesarean section. A free text box will allow participants to input what information they believe is vital to be shared with women about induction of labour and caesarean section.

All participants in the survey will also be invited to participate in the Delphi process (objective 2.2) if they consent for further contact.

Participants in the survey will be entered into a prize draw to win a voucher worth £10 (4 vouchers available).

Analysis:
The sample will be described using descriptive statistics and Key information points will be extracted from the survey data. These information points will be added to those from 1.1 and 1.2.

1.4 Identification of Key Information points from existing qualitative studies

Aim: To utilise data from existing qualitative datasets from studies led by the CI, which have focussed on information provision and decision making around birth.

Method: re-analysis of existing datasets where patients have provided their permission for data to be used for other studies. This will avoid research waste.

Dataset 1: Antenatal Care Education (ACE): The co-design and piloting of interventions to improve patient and staff experience through better birth preparedness. IRAS 262911, REC 19/SW/0073. We will re-analyse focus groups with women (46 participants) and Staff (21 participants) collected in 2019 which focussed on what information they wanted prior to birth to enable us to co-design an antenatal education session.

Dataset 2: Shared Decision making for labour and birth. IRAS 277301 REC 20/SW/0035. Dataset of 11 postnatal and 10 paired antenatal and postnatal interviews with women about how and when they wanted to receive information for decision making during birth and focus groups with 24 staff members.

Dataset 3: Vaginal Birth Core information set. University of Bristol Faculty of Health Sciences Research Ethics Committee ref 10530. 17 interviews focusing on information points around vaginal birth, but many women also discussed other modes of birth and induction of labour.

Analysis: These transcripts will be read and information points for induction or caesarean identified and added to the long list.

Objective 2: Delphi Process

2.1 Developing the Delphi

Aim: To produce the questionnaire for the first stage of the Delphi process

Method:
Members of the research team, patient contributors and stakeholders (up to 15 participants) will meet to develop the initial Delphi questionnaire using information items from 1.1-1.4. Items will be grouped, and categorised into domains before the final wording is agreed.

2.2 Piloting and refining of Delphi questionnaire

Aim: To ensure usability and clarity of the survey

Participants: Approximately 10 participants for each survey, from the aforementioned groups in 1.3.

Method:
Having recruited participants via email, online (Appendix 6) , via posters at the hospital/community areas (Appendix 7) or in person in the antenatal/postnatal clinics/wards at the hospital, they will be provided with a participant information leaflet (Appendix 8) approximately 10 think aloud interviews will be

carried out for each survey via secure teleconferencing software or inperson at Liverpool Women's Hospital or a nearby community site. Participants will be asked to complete a consent form, in RedCAP (Appendix 9) before completing the pilot questionnaire and being asked to 'think-aloud' while doing so. Think aloud interviews are a form of cognitive interviewing that asks participants to discuss the content of the survey they are completing whilst completing it. (Koskey 2016) This will allow us to identify what they understand by the questions and whether there are any sources of confusion or opportunities to clarify the content of the questions. At the end participants will be asked for general feedback on both the questionnaire and also if they feel any key information points were missing.

Notes will be taken by the interviewer, as well as the interviews being audio recorded on a University of Liverpool (UoL) encrypted device, downloaded to a UoL computer. The interviews will last upto one hour.

Analysis: The researcher will use their notes from the interview and listen to the recording to identify areas for improvement. After each think aloud interview, alterations will be made to the Delphi if necessary, to allow the testing of the new items with the next participant. The process will end when there are no further significant changes to the Delphi. Interviews will only be transcribed if necessary to ensure all points are noted and encompassed. In this case they will be transcribed either by the interviewer or a University approved service.

Participants will receive a £10 voucher.

2.3 <u>Modified Delphi Process</u>

Aim: To identify the information which should be shared with women when discussing induction of labour or caesarean section by developing two core information sets.

Participants:
-Women/birthing people, aged 16 or over, who are antenatal, planning a pregnancy or recently had a baby or their partners.
-Healthcare professionals who work alongside women in labour and postnatally (doctors, midwives, midwifery care assistants, physiotherapists)
-Medico legal experts who have an interest in obstetrics
-Representatives from groups who have an interest in women's birthing rights (e.g. Birthrights, Maternity Action)

Methods:
Recruitment: We will publicise the Delphi at the Hospital and in community settings using posters/promotional material/displays (Appendix 10&11) or in person in the antenatal/postnatal clinics/wards at the hospital. We will publicise the Delphi using the networks available through the CRN if sites are willing to display posters/cascade through their networks/via their social media. We will publicise the survey widely online, through our study social media, and social media of our partners, we will send individual invitations to experts and interested groups (Appendix 10). We may attend local community groups and provide ipads and any necessary support to complete the survey to ensure that disadvantaged groups/non-english speakers can be supported to participate in the Delphi Process.

Sample Size: We will obtain a sample with a broad range of experience, an approach supported by the COMET guidelines (Williamson 2017) and previous studies. We will aim to engage at least 100 participants in the Delphi process with 50 women, and 50 from across the other areas.

Process: Two-round modified Delphi process will be conducted online using REDCap software, with each round lasting for a minimum of 2 weeks. Participants will be asked to complete a consent before completing the survey (appendix 13). They will then be asked to complete basic demographic information as per the survey (Appendix 5). They will then be invited to score each survey item for importance for inclusion from 1-9 (1-3- limited importance; 4-6- important but not critical; 7-9- critical) . A-priori consensus criteria will be applied. Information items to be included will require ≥70% of participants from one stakeholder group to score it as critically important and <15% from any group to classify it as limited importance. Items will not be carried forward if over 70% of one group believe it is of limited importance, and if less than 15% believe it is critically important. Where items are rated as critical or there is no consensus, they will be carried into round two.

In round two participants will receive their own scores and the median scores for each item and will be asked to review and rescore each item. Items carried through to the consensus meetings will be selected based on the above criteria.

Participants in the survey will be entered into a prize draw to win a voucher worth £25. There will be 4 vouchers available for each survey.

2.4 Consensus meeting

Following the second Delphi round, an online consensus meeting will take place with participants from the online survey invited (up to 30 estimated). During this meeting, items that have survived the prior two rounds will be discussed and the core information set finalised.

A consensus meeting with participants from the different stakeholder groups. We will aim to have 30 participants in attendance. Participants will be provided with an information sheet (Appendix 12) asked to complete a consent form prior to the meeting (appendix 13). During this meeting, the participants will anonymously rate the retained information domains from the second round. Histograms and descriptive statistics will be created for each domain during the meetings and displayed to participants, to stimulate discussion. Through discussion and prioritisation, a consensus will be formed on the most important items. Where consensus is not reached, there will be further discussion and additional voting. All items retained from both meetings will be combined to form a final short list of information items to be included in the core information set.

To ensure that no elements of the discussion are missed, the meeting will be recorded on a University of Liverpool encrypted recorder and transferred to a University secure server. The recording may be transcribed by a University approved transcription service if the research team decide that this is necessary.

4 STUDY SETTING

We will recruit patients at Liverpool Women's hospital and work with the Clinical Research Network to publicise via their networks. At sites other than Liverpool Women's hospital we will be asking them to display recruitment posters/leaflets only, not directly approach patients. We will also recruit online, through social media to gain national coverage. We will undertake interviews and meetings via telephone and teleconference software.

5 SAMPLE AND RECRUITMENT

5.1 Eligibility Criteria

5.1.1 Inclusion criteria

Women:
(Online survey; Think-aloud interviews; Delphi survey & consensus meeting)
Any woman, who is over the age of 16, who is planning to be pregnant, currently pregnant, or has had a baby recently*.

The Delphi survey and all study documents will be available in English, Somali and Arabic. These languages have been chose because they are two of the more common non-European languages spoken in the Liverpool area. Therefore, participants need to be able to understand the surveys in these three languages.

*we have deliberately not defined the eligible postnatal period, so that women/birthing people who wish to share their views, even if their experiences are not immediate, can be included.

Healthcare professionals:
(Online survey; Delphi survey & consensus meeting)
Those who work with, or provide care for, women during labour and postnatally (e.g. obstetricians and gynaecologists include those in training, midwives, midwifery support workers, physiotherapists, anaesthetists). They will be recruited from hospital trusts/GP practices, online, through social media.

Medicolegal experts
(Online survey; Think-aloud interviews; Delphi survey & consensus meeting)
Self identified medico-legal experts whether legal (e.g. solicitors/barristers involved in medicolegal claims), academic or medics specialising in medico-legal claims.

Representatives from interested groups
(Online survey; Think-aloud interviews; Delphi survey & consensus meeting)
Representatives from groups who have an interest in women's health/birth/rights/information(e.g. Birthrights, Maternity Action, NCT).

5.1.2 Exclusion criteria

Women: Women who are under the age of 16. Women who are not able to give informed consent to participate in the study and women who do not understand/read one of the study languages.

5.2 Sampling

5.2.1 Size of sample

1.2 Online survey
We will aim to engage approximately 100 survey participants. This number of responses to the survey will allow a diverse group of participants.

1.3 Pilot of Delphi questionnaire and 'think aloud' interviews
We estimate that we will interview upto 10 participants for each topic. This estimate is based on previously conducted 'think-aloud' studies. Interviews will continue until data saturation is achieved and no further amendments of the Delphi survey are required.

2.1 & 2.2 Delphi survey and consensus meeting
There are no generally accepted guidelines for the optimal size to achieve a consensus in Delphi Studies and decision on how many individuals to include in a Delphi process is not based on statistical power and is a pragmatic decision. The group size is often dependent on the number of experts and patients available within the scope of the core information set. Careful consideration will be made to sample stakeholders with a breadth of experience. The assumptions made for this Delphi are based on COMET guidelines and previous studies. Typically stakeholder groups have included 13 to 222 participants and attrition rates in previous core outcome sets have ranged from 0-20%. One study of published core outcome sets, found the average response rate in round 2 for a two-round Delphi is 80%, with no evidence of attrition bias. Furthermore, it found higher number of items in the Delphi was associated with a lower response rate. In this study, a minimum of 100 participants in total will be recruited to account for a 20% drop-out rate. We will aim for at least half these participants to be women and partners as their views are key to this study. We will also carefully manage the number of items that will be included in the final Delphi survey. Previous core outcome set developers have aimed for less than 100 initial items for participants to prioritise.

The consensus meetings will consist of approximately 30 participants from those who took part in the Delphi survey rounds.

5.3 Recruitment
5.3.1 Sample identification

Participants will self-identify via responding to one of the posters/adverts/social media adverts. Specific stakeholder groups will be approached for participation in the survey by looking their contact details up online e.g. women's health organisations, law firms with expertise in obstetric claims.

5.3.2 Consent

1.2 Online survey
A copy of the study information sheet will be emailed to interested participants. A URL will lead the participants to the survey on REDCap. At the start of the survey, participants will be asked complete an online consent form on the software (Appendix 4). The participants will be required to complete this form in order to be able to undertake the survey.

1.3 Pilot of Delphi questionnaire and 'think aloud' interviews

Interested participants will be invited to take part in the study by an advert (Appendix 6 and 7) and a copy of the study information sheet will be emailed to interested participants (Appendix 8). After 24-48 hours the researcher will contact them to answer any questions they may have, and determine whether they wish to take part. Once they have agreed to participate, they will be emailed the details of their appointment for interview, along with an url link to an online consent form hosted in REDCap (Appendix 9). Participants will be required to complete this form before they join the interview.

2.1 Delphi Survey

Participants will be invited to participate online link via social media or a QR code on the poster (Appendix 10/11). Prior to commencing the survey, they will be directed the participant information leaflet to read (Appendix 12). The importance of participating in every round will be emphasised. Informed consent will be obtained from all participants who agree to take part prior to participation in the survey. Consent will be taken online via RedCap (Appendix 13). Participants contact information (name and email address) will be retained until the core information set is complete and they have been emailed the final core information set, at which point their personal data will be deleted unless they have opted to join the study mailing list.

2.2 Consensus meetings

Stakeholders will be asked if they are willing to participate in the consensus meeting in the final question of the Delphi questionnaire in round two and will invited to the meeting once the analysis has been completed. Interested participants will be invited to take part in the consensus meetings by email and a copy of the participant information sheet will be emailed to them (Appendix 15). Once they have agreed to participate, they will be emailed the details of the meeting, along with a url link to an online consent form hosted in REDCap (Appendix 15). Participants will be required to complete this form before they join the meeting.

6 ETHICAL AND REGULATORY CONSIDERATIONS

6.1 Assessment and management of risk

Woman reports issues concerning poor practice
We will encourage her to engage with the patient liaison team at her relevant maternity unit.

Distress during think-aloud interviews or consensus meeting
It is possible that participants may experience distress during the 'think-aloud' interviews or consensus meetings. Should a participant become distressed in the interview, they will be offered the opportunity to pause the interview, and if they choose, to stop it completely. Similarly, they will be offered to leave the consensus meeting if they choose. This will be explained to the participants prior to the interview and consensus meetings commencing. Furthermore, in the consensus meetings there might be conflict between parents and the professionals which may cause upset. The amount of distress a participant experiences will be judged on an individual level and it is expected that responses and reactions may vary.

There will be the opportunity to debrief with the parents after the group discussion and they can be directed to additional support if required. Should any participants become distressed, the distress protocol will be followed (see appendix 16). Participants will be encouraged to contact their own healthcare provider, GP, friend or family member. Alternatively, if appropriate and with participant consent to share the contact with the research team, a member of the research team could contact a form of support for them.

6.2 Research Ethics Committee (REC) and other Regulatory review & reports

The study will be performed subject to favourable opinion, authorisation and permission from all necessary regulatory and other bodies. This includes but is not limited to the Health Research Authority (HRA), a UK Research Ethics Committee and the NHS.
This study will be conducted in accordance with:

- International Conference for Harmonisation guidelines for Good Clinical Practice (ICH GCP)
- UK Policy Framework for Health and Social Care Research

Before commencement the CI will obtain the formal 'go-ahead' from the Host and Sponsor.

If amendments to the protocol and/or the study documents are required, relevant approvals will be sought from the ethics committee. The CI will be responsible for decisions to amend the protocol and the sponsor will determine whether the amendment is substantial or non-substantial. Relevant study documents will be altered, and the changes made tracked to demonstrate where they have been made.
Amendments to the protocol will be documented using sequential version numbers, with updated documents replaced in the site file.

6.3 Peer review

This study has been reviewed as part of a competitive application process for funding.

6.5 Protocol compliance

It is known that accidental protocol deviations can happen. These will be reported to the CI. Causes of deviation will be identified and procedures put in place to mitigate them.

6.5.1 Auditing

The study may be subject to inspection and audit by the University of Liverpool under their remit as sponsor and other regulatory bodies to ensure adherence to GCP and the UK Policy Framework for Health and Social Care Research (v3.2 10th October 2017).

Safety Monitoring

This study is a Delphi/interview study with no intervention, therefore the risks associated have been documented above and beyond this there are unlikely to be any safety related events.

Provision has been made through the distress policy (appendix 14) of dealing with adverse events, for example, psychological distress.

6.5.2 Protocol Violations

Participants who do not follow the planned protocol are considered to have a protocol violation. The nature of the protocol violation will be documented for each participant and reported to the Sponsor.

6.5.3 Withdrawal

If a participant wishes to withdraw, they will inform a member of the research team, whose contact details will be on the participant information sheet. These options will be made clear to participants in the participant information sheets, on the consent form, as well as verbally by the interviewer prior to commencement of the research.

6.6 Confidentiality

The Chief Investigator will preserve the confidentiality of participants taking part in the study and will abide by the Data Protection Act 2018 and the UK GDPR as amended from time to time and any successor legislation in the UK and any other directly applicable regulation relating to data protection and privacy.

All data collected for this study will be collected and managed at the University of Liverpool, in accordance with the University of Liverpool procedures. Data will be collected and retained in accordance with the General Data Protection Regulation (GDPR) (EU) 2016/679 and within the principles of Good Clinical Practice.

Once data collection has been completed and data files have been deidentified, all data will be stored on an the University of Liverpool secure password protected server the Active Datastore for data analyses, write-up, and dissemination. Prior to this, some data will be stored in the University of Liverpool RedCAP servers.

Audio recordings will be recorded on encrypted University of Liverpool recording devices and transferred into the UoL Active Datastore. Audio recordings will be deleted once the core information sets are complete and published. Any transcription that is required will be transferred securely to a University of Liverpool approved transcription service and the anonymised transcripts will then be stored on in the University Active Datastore.

The data will be kept confidential and protected through fully compliant UK Data Security requirements. Participant consent forms which will be completed electronically will be stored electronically in a password protected file in the University Active Datastore server and will be destroyed 10 years after the study is published.

Survey data will be separated from identifiable data before download and saved on a password protected file on the University Active Datastore secure server.

When interpreters are used we will use where possible the interpreting service used by the local NHS site, which have existing confidentiality agreements in place with the local trust. If we are using translation services, we will use the same procedures for them as the transcription services, putting confidentiality agreements in place to ensure that participant data remains confidential. It will not be necessary to disclose participant identifiable data to any translator."

Participant data will include:
Audio files (electronic), electronic consent forms, electronic copies of survey and Delphi responses.

Personal data will be treated in strict confidence. Participants' personal information, including name, and email address (and for some participants a telephone number) is required in order for the research team to keep in contact with participants for the duration of the research. Participants will be advised that if they wish to check what personal data the Study team are holding for this research, they should contact the CI. This data will be destroyed once the information set is completed and disseminated to the participants unless they request to remain on the wider study mailing list.

Aside from any planned contact, outlined in this protocol, the only time the research team would use personal data would be if there was a concern that a participant was at risk of harm due to their involvement in the study. If there was a need to inform relevant authorities of risks to participants, the study team would discuss this first with participants and encourage them to seek appropriate help as detailed in the distress policy.

Research data will be anonymised before analysis.

<u>Data Storage</u>
All data stored on the University of Liverpool Active Datastore will be accessible to only the relevant members of the research team. As the data is relevant to this study only, and is unlikely to have broader scientific relevance, it will be retained for least 10 years after, the publication of the study and then destroyed.

6.7 Access to the final study dataset

The final study data set will be stored in the UOL Active Datastore relevant members of the study team will have access to this data. Individuals in the wider steering group will receive parts of the data, as required, for analysis and discussion.

It is not envisaged that this data will be used for analysis outside the remit of this study, however, we will ask permission to use the data for subsequent analysis in the consent form.

6.8 End of study Definition

The end of the study will be when the final core information sets are produced. After this time the only remaining activity will be preparation of the findings for dissemination.

6.9 Indemnity

The University of Liverpool holds Indemnity and insurance cover with Newline Insurance Company, which apply to this study.

7 DISSEMINATION POLICY

7.1 Dissemination Plans

Communication and dissemination will be an agenda item at study team meetings to ensure the identification of appropriate dissemination strategies to maximise potential uptake of the research findings. A publication plan will also be developed and be subject to ongoing refinement as this study progresses. We will provide a synopsis for this study on a Study web page. We will also invite participants to be part of a mailing list and provide brief accessible reports via a study newsletter. We will submit to national conferences to disseminate our work and engage our peers for any future implementation or larger studies.

Our collaborative links will allow us to expand the reach of our findings and engage with the wider research community, key stakeholders, peers, patients, and the public. Furthermore, we will liaise closely with our patient representatives to ensure our findings are accessible to a wide audience. We will co-produce and disseminate a short, briefing paper.

On completion of the Study, the data will be analysed and reported back to the Funder and REC.

Due to the nature of this study, we do not intend on making the dataset publicly accessible, however, if other researchers/quality improvement teams request the data we will be happy to share it with them if appropriate ethical approvals are gained.

7.2 Authorship eligibility guidelines and any intended use of professional writers

Authorship will be granted to all members of the study team including patient representatives who participate in the design of the study, data collection or analysis of results and contribute to the final paper.

8 REFERENCES

Blazeby JM, Macefield R, Blencowe NS, Jacobs M, McNair AGK, Sprangers M, et al. Core information set for oesophageal cancer surgery. British Journal of Surgery. 2015 Jun 10;102(8):936–43.

Briscoe KE, Haas DM. Developing a Core Outcome Set for Cesarean Delivery Maternal Infectious Morbidity Outcomes. American Journal of Perinatology. 2020 Mar 28;37(04):436–52.

Dos Santos F, Drymiotou S, Antequera Martin A, Mol BW, Gale C, Devane D, van't Hooft J, Johnson MJ, Hogg M, thangaratinam S. Development of a core outcomes set for trials on induction of labour: an international multistakeholder Delphi.

Gammie N, Key S. Time's up! Women's experience of induction of labour. The practising midwife. 2014 Apr;17(4):15–8

Koskey KLK. Using the cognitive pretesting method to gain insight into participants' experiences: An illustration and methodological reflection. International Journal of Qualitative Methods. 2016;15(1):1-13. doi:10.1177/1609406915624577

Lavender T, Walkinshaw SA, Walton I. A prospective study of women's views of factors contributing to a positive birth experience. Midwifery. 1999 Mar;15(1):40–6.

McNair AGK, Whistance RN, Main B, Forsythe R, Macefield R, Rees J, et al. Development of a core information set for colorectal cancer surgery: a consensus study. BMJ Open. 2019 Nov 14;9(11):e028623.

NHS England and NHS Improvement. Shared Decision Making: Summary Guide. Available from: https://www.england.nhs.uk/publication/shared-decision-making-summary-guide/ accessed on 28th November 2022

Williamson PR, Altman DG, Bagley H, et al. The COMET Handbook: Version 1.0. Trials. 2017;18(Suppl 3):1-50. doi:10.1186/s13063-017-1978-4

9. APPENDICIES

Appendix 1: Social media adverts for survey
Appendix 2: Poster for stakeholder survey
Appendix 3: Survey
Appendix 4: Consent text for online Survey
Appendix 5: Survey participant information sheet
Appendix 6: Social media advert for think-aloud interviews
Appendix 7: Think-aloud recruitment poster
Appendix 8: Think-aloud participant information sheet
Appendix 9: Think-aloud consent form
Appendix 10: Delphi survey advert
Appendix 11: Delphi survey poster
Appendix 12: Delphi Participant Information Sheet
Appendix 13: Consent text for Delphi survey
Appendix 14: Consensus meeting Participant information sheet
Appendix 15: Consent form for consensus meeting
Appendix 16: Distress policy.
