# Supplementary material for: What should be discussed when considering an induction of labour? A UK-wide, multi-centre Delphi study to develop a core information set for induction of labour
Source: BMJ Open. 2026 May 27;16(5):e118024. doi: 10.1136/bmjopen-2026-118024 (PMC13218194; doi:10.1136/bmjopen-2026-118024)
Supplement: online supplemental file 2 [file bmjopen-16-5-s002.pdf]

Supplementary File 2: COS-STAR Statement Checklist adapted for Core Information Set

SECTION/TOPIC	ITEM No.	CHECKLIST ITEM	REPORTED ON PAGE NUMBER
TITLE/ABSTRACT			
Title	1a	Identify in the title that the paper reports the development of a CIS	1
Abstract	1b	Provide a structured summary	2
INTRODUCTION			
Background and Objectives	2a	Describe the background and explain the rationale for developing the CIS.	4
	2b	Describe the specific objectives with reference to developing a CIS.	5
Scope	3a	Describe the health condition(s) and population(s) covered by the CIS.	4-5
	3b	Describe the intervention(s) covered by the CIS.	4-5
	3c	Describe the setting(s) in which the CIS is to be applied.	4-5
METHODS			
Protocol/Registry Entry	4	Indicate where the CIS development protocol can be accessed, if available, and/or the study registration details.	1
Participants	5	Describe the rationale for stakeholder groups involved in the CIS development process, eligibility criteria for participants from each group, and a description of how the individuals involved were identified.	6
Information Sources	6a	Describe the information sources used to identify an initial list of information items.	6
	6b	Describe how items were dropped/combined, with reasons (if applicable).	6
Consensus Process	7	Describe how the consensus process was undertaken.	7-8
Item Scoring	8	Describe how items were scored and how items were summarised.	8
Consensus Definition	9a	Describe the consensus definition.	8
	9b	Describe the procedure for determining how items were included or excluded from consideration during the consensus process.	8

Ethics and Consent	10	Provide a statement regarding the ethics and consent issues for the study.	5
RESULTS			
Protocol Deviations	11	Describe any changes from the protocol (if applicable), with reasons, and describe what impact these changes have on the results.	N/A
Participants	12	Present data on the number and relevant characteristics of the people involved at all stages of CIS development.	Table 1 and 2
Items	13a	List all items considered at the start of the consensus process.	Supplementary file 8
	13b	Describe any new items introduced and any items dropped, with reasons, during the consensus process.	Supplementary file 10,11
CIS	14	List the items in the final CIS.	Figure 2
DISCUSSION			
Limitations	15	Discuss any limitations in the CIS development process.	12-13
Conclusions	16	Provide an interpretation of the final CIS in the context of other evidence, and implications for future research.	11-13
OTHER INFORMATION			
Funding	17	Describe sources of funding/role of funders.	14
Conflicts of Interest	18	Describe any conflicts of interest within the study team and how these were managed.	14

From: Kirkham JJ, Gorst S, Altman DG, Blazeby JM, Clarke M, Devane D, et al. (2016) Core Item Set– STAndards for Reporting: The COS-STAR Statement. PLoS Med 13(10): e1002148. https://doi.org/10.1371/journal.pmed.1002148
