# Supplementary material for: What should be discussed when considering an induction of labour? A UK-wide, multi-centre Delphi study to develop a core information set for induction of labour
Source: BMJ Open. 2026 May 27;16(5):e118024. doi: 10.1136/bmjopen-2026-118024 (PMC13218194; doi:10.1136/bmjopen-2026-118024)
Supplement: online supplemental file 3 [file bmjopen-16-5-s003.pdf]

Request: Labour induction systematic reviews

Database	Date searched	Results
MEDLINE (Ovid)	27/2/23	237
EMBASE (Ovid)	27/2/23	420
CINAHL (EBSCOHOST)	27/2/23	194
PsycINFO (EBSCOHOST)	6/3/23	11
The Cochrane Library (CSDR)	27/2/23	20
NIHR Journals Library [HTA/Evidence Synthesis] https://www.journalslibrary.nihr.ac.uk/#/	27/2/23	0
International Network of Agencies for Health Technology Assessment (INAHTA) https://database.inahta.org	27/2/23	0
Epistemonikos https://www.epistemonikos.org/	27/2/23	206
Health Evidence https://www.healthevidence.org/	27/2/23	0
DoPHER (Database of promoting health effectiveness reviews) https://eppi.ioe.ac.uk/cms/Default.aspx?tabid=62	27/2/23	0
**Total across all databases**		1175
**Duplicates removed**		626
**Total in Endnote**		**549**

Note: Searches limited to systematic reviews, English language, 2018-2023

Ovid MEDLINE(R) ALL <1946 to February 27, 2023>

1	exp Labor, Induced/
2	(induc* adj3 labor).mp.
3	(induc* adj3 labour).mp.
4	((routine or elective) adj induction).mp.
5	(stimulat* adj3 labor).mp.
6	(stimulat* adj3 labour).mp.
7	membrane sweep*.mp.
8	mechanical cervical ripening.mp.
9	amniotomy.mp.
10	artificial rupture of the membranes.mp.
11	pharmacological cervical ripening.mp.
12	(balloon catheter and (labour or labor or pregnan* or cervical ripening)).mp.
13	((dinoprostone or prostaglandin*) adj5 (labour or labor or cervical ripening)).mp.
14	1 or 2 or 3 or 4 or 5 or 6 or 7 or 8 or 9 or 10 or 11 or 12 or 13
15	systematic review.pt.
16	meta-analysis.pt.
17	systematic review/
18	meta analysis/
19	network meta-analysis/
20	(0266-4623 or 1469-493X or 1366-5278 or 1530-440X or 2046-4053).is.
21	(systematic review? or evidence report* or technology assessment?).jw.
22	(meta-analys* or meta analys* or metaanalys* or meta synth* or meta-synth* or metasynth*).mp.
23	((systematic or meta) adj2 (analys* or review)).ti,kw.
24	((systematic* or methodologic*) adj5 (review* or overview*)).mp.
25	(integrative research review* or research integration or (evidence adj3 review*)).mp.
26	("review of reviews" or umbrella review).mp.
27	(overview adj2 review*).mp.
28	cochrane review*.mp.
29	(quantitativ* adj5 synthes*).mp.
30	(qualitativ* adj5 synthes*).mp.
31	(realist adj (review or synthes*)).mp.
32	(rapid review or rapid evidence assessment).mp.
33	(mixed method? adj1 (review or synthes*)).mp.
34	framework synthes*.mp.
35	(metaethnography or meta-ethnography).mp.
36	(meta-aggregation or meta-interpretation).mp.
37	(metatheory or meta-theory or metastudy or meta-study).mp.
38	(thematic synthes* or thematic analys*).mp. and review.pt,ti.
39	"interpretive synthes*".mp.
40	(narrative adj (review or synthes*)).mp.
41	((scoping or mapping) adj1 review).mp.
42	or/15-41
43	14 and 42
44	limit 43 to (english language and yr="2018 -Current")

Embase <1974 to 2023 February 27>

1 exp labor induction/
2 (induc* adj3 labor).mp.
3 (induc* adj3 labour).mp.
4 ((routine or elective) adj induction).mp.
5 (stimulat* adj3 labor).mp.
6 (stimulat* adj3 labour).mp.
7 membrane sweep*.mp.
8 mechanical cervical ripening.mp.
9 amniotomy.mp.
10 artificial rupture of the membranes.mp.
11 pharmacological cervical ripening.mp.
12 (balloon catheter and (labour or labor or pregnan* or cervical ripening)).mp.
13 ((dinoprostone or prostaglandin*) adj5 (labour or labor or cervical ripening)).mp.
14 or/1-13
15 "systematic review"/
16 meta analysis/
17 network meta-analysis/
18 (0266-4623 or 1469-493X or 1366-5278 or 1530-440X or 2046-4053).is.
19 (systematic review? or evidence report* or technology assessment?).jw.
20 (meta-analys* or meta analys* or metaanalys* or meta synth* or meta-synth* or metasynth*).mp.
21 ((systematic or meta) adj2 (analys* or review)).ti,kw.
22 ((systematic* or methodologic*) adj5 (review* or overview*)).mp.
23 (integrative research review* or research integration or (evidence adj3 review*)).mp.
24 ("review of reviews" or umbrella review).mp.
25 (overview adj2 review*).mp.
26 cochrane review*.mp.
27 (quantitativ* adj5 synthes*).mp.
28 (qualitativ* adj5 synthes*).mp.
29 (realist adj (review or synthes*)).mp.
30 (rapid review or rapid evidence assessment).mp.
31 (mixed method? adj1 (review or synthes*)).mp.
32 framework synthes*.mp.
33 (metaethnography or meta-ethnography).mp.
34 (meta-aggregation or meta-interpretation).mp.
35 (metatheory or meta-theory or metastudy or meta-study).mp.
36 (thematic synthes* or thematic analys*).mp. and review.pt,ti.
37 "interpretive synthes*".mp.
38 (narrative adj (review or synthes*)).mp.
39 ((scoping or mapping) adj1 review).mp.
40 or/15-39
41 14 and 40
42 limit 41 to (english language and yr="2018 - 2023")

CINAHL via EBSCOHOST

S1 (MH "Labor, Induced+")
S2 induc* N3 labor
S3 induc* N3 labour
S4 (routine or elective) N0 induction
S5 stimulat* N3 labor
S6 stimulat* N3 labour
S7 "membrane sweep*"
S8 "mechanical cervical ripening"
S9 amniotomy
S10 "artificial rupture of the membranes"
S11 "pharmacological cervical ripening"
S12 (balloon catheter and (labour or labor or pregnan* or cervical ripening))
S13 ((dinoprostone or prostaglandin*) N5 (labour or labor or cervical ripening))
S14 S1 OR S2 OR S3 OR S4 OR S5 OR S6 OR S7 OR S8 OR S9 OR S10 OR S11 OR S12 OR S13
S15 PT systematic review
S16 PT meta analysis
S17 PT meta synthesis
S18 IS (0266-4623 or 1469-493X or 1366-5278 or 1530-440X or 2046-4053)
S19 (systematic review? or evidence report* or technology assessment?)
S20 (meta-analys* or meta analys* or metaanalys* or meta synth* or meta-synth* or metasynth*)
S21 TI (((systematic or meta) N2 (analys* or review))) OR SU (((systematic or meta) N2 (analys* or review)))
S22 ((systematic* or methodologic*) N5 (review* or overview*))
S23 (integrative research review* or research integration or (evidence N3 review*))
S24 ("review of reviews" or umbrella review)
S25 (overview N2 review*)
S26 "cochrane review*"
S27 (quantitativ* N5 synthes*)
S28 (qualitativ* N5 synthes*)
S29 (realist N0 (review or synthes*))
S30 (rapid review or rapid evidence assessment)
S31 (mixed method? N1 (review or synthes*))
S32 "framework synthes*"
S33 (metaethnography or meta-ethnography)
S34 (meta-aggregation or meta-interpretation)
S35 (metatheory or meta-theory or metastudy or meta-study)
S36 ((thematic synthes* or thematic analys*)) AND PT review
S37 ((thematic synthes* or thematic analys*)) AND TI review
S38 "interpretive synthes*"
S39 (narrative N0 (review or synthes*))
S40 ((scoping or mapping) N1 review)
S41 (MH "Systematic Review")
S42 (MH "Cochrane Library")
S43 (MH "Meta Analysis")
S44 (MH "Meta Synthesis")
S45 S16 OR S17 OR S18 OR S19 OR S20 OR S21 OR S22 OR S23 OR S24 OR S25 OR S26 OR S27 OR S28 OR S29 OR S30 OR S31 OR S32 OR S33 OR S34 OR S35 OR S36 OR S37 OR S38 OR S39 OR S40 OR S41 OR S42 OR S43 OR S44
S46 S14 AND S45 Limiters - Publication Year: 2018-2023. Narrow by Language: - english

PsycINFO via EBSCOHOST

S1 induc* N3 labor
S2 induc* N3 labour
S3 (routine or elective) N0 induction
S4 stimulat* N3 labor
S5 stimulat* N3 labour
S6 "membrane sweep*"
S7 "mechanical cervical ripening"
S8 amniotomy
S9 "artificial rupture of the membranes"
S10 "pharmacological cervical ripening"
S11 (balloon catheter and (labour or labor or pregnan* or cervical ripening))
S12 ((dinoprostone or prostaglandin*) N5 (labour or labor or cervical ripening))
S13 S1 OR S2 OR S3 OR S4 OR S5 OR S6 OR S7 OR S8 OR S9 OR S10 OR S11 OR S12
S14 (systematic review? or evidence report* or technology assessment?)
S15 (meta-analys* or meta analys* or metaanalys* or meta synth* or meta-synth* or metasynth*)
S16 TI (((systematic or meta) N2 (analys* or review))) OR SU (((systematic or meta) N2 (analys* or review)))
S17 ((systematic* or methodologic*) N5 (review* or overview*))
S18 (integrative research review* or research integration or (evidence N3 review*))
S19 ("review of reviews" or umbrella review)
S20 (overview N2 review*)
S21 "cochrane review*"
S22 (quantitativ* N5 synthes*)
S23 (qualitativ* N5 synthes*)
S24 (realist N0 (review or synthes*))
S25 (rapid review or rapid evidence assessment)
S26 (mixed method? N1 (review or synthes*))
S27 "framework synthes*"
S28 (metaethnography or meta-ethnography)
S29 (meta-aggregation or meta-interpretation)
S30 (metatheory or meta-theory or metastudy or meta-study)
S31 (((thematic synthes* or thematic analys*))) AND TI review
S32 "interpretive synthes*"
S33 (narrative N0 (review or synthes*))
S34 ((scoping or mapping) N1 review)
S35 DE "Systematic Review"
S36 DE "Meta Analysis"
S37 S14 OR S15 OR S16 OR S17 OR S18 OR S19 OR S20 OR S21 OR S22 OR S23 OR S24 OR S25 OR S26 OR S27 OR S28 OR S29 OR S30 OR S31 OR S32 OR S33 OR S34 OR S35 OR S36
S38 S13 AND S37
S39 S13 AND S37 Limiters - Published: 20180101-20231231, Narrow by Language: - english

The Cochrane Library (CSDR) via The Cochrane Library

#1 MeSH descriptor: [Labor, Induced] explode all trees

#2 (induc* NEAR/3 labor):ti,ab,kw
#3 (induc* NEAR/3 labour):ti,ab,kw
#4 (routine NEXT induction):ti,ab,kw
#5 (elective NEXT induction):ti,ab,kw
#6 (stimulat* NEAR/3 labor):ti,ab,kw
#7 (stimulat* NEAR/3 labour):ti,ab,kw
#8 (membrane NEXT sweep*):ti,ab,kw
#9 ("mechanical cervical ripening"):ti,ab,kw
#10 (amniotomy):ti,ab,kw
#11 ("artificial rupture of the membranes"):ti,ab,kw
#12 ("pharmacological cervical ripening"):ti,ab,kw
#13 (balloon catheter and (labour or labor or pregnan* or cervical ripening)):ti,ab,kw
#14 (dinoprostone NEAR/5 (labour or labor or cervical ripening)):ti,ab,kw
#15 (prostaglandin* NEAR/5 (labour or labor or cervical ripening)):ti,ab,kw
#16 {OR #1-#15}

NIHR Journals Library
[HTA/Evidence synthesis records] https://www.journalslibrary.nihr.ac.uk/#/

labour AND induc*
Limit to 2018-2023

International Network of Agencies for Health Technology Assessment (INAHTA)
https://database.inahta.org

((((labour)[title]) OR ((labor)[title])) AND ((induc*)[title])) OR ("Labor, Induced"[mhe])
Limit to 2018-2023

Epistemonikos
https://www.epistemonikos.org/

(title:((title:(labour OR labor) OR abstract:(labour OR labor)) AND (title:(induc*) OR abstract:(induc*))) OR abstract:((title:(labour OR labor) OR abstract:(labour OR labor)) AND (title:(induc*) OR abstract:(induc*)))) OR (title:("membrane sweep*") OR abstract:("membrane sweep*")) OR (title:("routine induction") OR abstract:("routine induction")) OR (title:("elective induction") OR abstract:("elective induction")) OR (title:("mechanical cervical ripening") OR abstract:("mechanical cervical ripening")) OR (title:(amniotomy) OR abstract:(amniotomy)) OR (title:("artificial rupture of the membranes") OR abstract:("artificial rupture of the membranes")) OR (title:("pharmacological cervical ripening") OR abstract:("pharmacological cervical ripening")) OR (title:((balloon catheter AND (labour OR labor OR pregnan* OR cervical ripening))) OR abstract:((balloon catheter AND (labour OR labor OR pregnan* OR cervical ripening)))) OR (title:(((dinoprostone OR prostaglandin*) AND (labour OR labor OR cervical ripening))) OR abstract:(((dinoprostone OR prostaglandin*) AND (labour OR labor OR cervical ripening)))) OR (title:(stimulat* AND (labour OR labor)) OR abstract:(stimulat* AND (labour OR labor)))
Limit to Systematic reviews, 2018-2023

Health Evidence
https://www.healthevidence.org/

Results for: [(labour OR labor) AND induc*] AND Limit:

Date = Published from 2018 to 2023

Review Type = Meta-analysis, Narrative review, Systematic review of reviews

DoPHER (Database of promoting health effectiveness reviews)
https://eppi.ioe.ac.uk/cms/Default.aspx?tabid=62
Browse through list of titles

Centre for Reviews and Dissemination
https://www.york.ac.uk/crd/
