# Supplementary material for: What should be discussed when considering an induction of labour? A UK-wide, multi-centre Delphi study to develop a core information set for induction of labour
Source: BMJ Open. 2026 May 27;16(5):e118024. doi: 10.1136/bmjopen-2026-118024 (PMC13218194; doi:10.1136/bmjopen-2026-118024)
Supplement: online supplemental file 4 [file bmjopen-16-5-s004.pdf]

NHS trusts	**Non NHS Trusts**	**International**
North Bristol Hospital	The Royal College of Midwives	Chester County Paediatrics
St Michael's Hospital	Tommy's	The American College of Obstetricians and Gynaecologists
Saint Mary's Hospital	National Childbirth Trust	HSE Ireland
Nottingham University Hospitals	Baby Centre UK	Mayo Clinic
Royal Devon and Exeter Hospital	Healthier Together	Health Hub Singapore
King's College Hospital		RANZCOG
Earth and North Hertfordshire Hospital		Pregnancy Birth & Baby Au
St George's University Hospitals		New Zealand College of Midwives
Liverpool Women's Hospital		The Society of Obstetricians and Gynaecologists of Canada
Harrogate and District Hospital		India Parenting
Imperial College Healthcare		
Salisbury Hospital		
Birmingham Women's and Children's Hospital		
Bradford Teaching Hospitals		
University Hospital Southampton		
Hull University Teaching Hospitals		
The Leeds Teaching Hospitals		
University Hospitals of Leicester		
Norfolk and Norwich University Hospitals		

North West Anglia Hospital
GIG CYMRU NHS Wales
South Tyneside and Sunderland Hospital
Chelsea and Westminister Hospital
James Cook Hospital
York Teaching Hospital
Guy's and St Thomas' Hospital
Firmley Health
Royal Free London Hospital
University Hospitals Coventry and Warwickshire
Buckinghamshire Healthcare
Cambridge University Hospitals
Sherwood Forest Hospitals
University Hospitals Dorset
Maidstone and Tunbridge Wells Hospital
Great Western Hospitals
Calderdale and Huddersfield Hospital
United Lincolnshire Hospitals
Kingston Hospital
Whittington Health
The Shrewsbury and Telford Hospital
County Durham and Darlington Hospital
Sheffield Teaching Hospitals

James Paget University Hospitals
Royal Berkshire Hospital
Blackpool Teaching Hospitals
The Newcastle upon Tyne Hospitals
NHS Forth Valley
East Lancashire Hospital
North Tees and Hartlepool Hospital
Warrington and Halton Teaching Hospitals
Dartford and Gravesham Hospital
Walsall Healthcare
Medway
Barnsley Hospital
Homerton Healthcare
Milton Keynes University Hospital
Surrey and Sussex Healthcare
