# Supplementary material for: What should be discussed when considering an induction of labour? A UK-wide, multi-centre Delphi study to develop a core information set for induction of labour
Source: BMJ Open. 2026 May 27;16(5):e118024. doi: 10.1136/bmjopen-2026-118024 (PMC13218194; doi:10.1136/bmjopen-2026-118024)
Supplement: online supplemental file 5 [file bmjopen-16-5-s005.pdf]

Development of a Core Information Set for Induction of Labour & Caesarean

Thank you for taking the time to look at our survey. This is one part of a study which is looking at understanding what information women / birthing people need to know before they make the decision to have an induction of labour / caesarean birth. As part of this we want to incorporate the views and experiences of as many different people as possible.

We are therefore inviting women who are either currently pregnant, planning a pregnancy or who recently had a baby and their partners, healthcare professionals who work with pregnant women / birthing people, medico-legal experts and individuals who are part of charities interested in maternity care to complete a survey about what information they believe is important for women / birthing people to know about induction of labour / caesarean section. The survey should take no more than 15 minutes of your time.

You will not be identifiable in your answers. You do not need to leave us your contact details at all unless you would like to. However, you will be invited to leave your contact details to be entered into a prize draw for a £10 voucher (4 are available). In addition to this, you will be invited to leave your details if you would like to receive the results of this study. We will also ask if you would be willing to be contacted by the research team to take part in later parts of this study. If you answer 'yes' to any of these, we ask that you leave your email address.

Your participation is entirely voluntary, and you can stop completing the questionnaire at any time and you can withdraw your participation from the study at any time for 2 weeks after you complete the questionnaire, as long as you have left us your email address.

For the full information leaflet about this study please click this link.

If you would like further information about this study as a whole please click this link.

If you have any concerns to raise about the conduct of this study, please contact sponsor@liverpool.ac.uk.

If you have any queries about the study, please email the study team on options@liverpool.ac.uk.

Would you like to be entered into a prize draw for a chance to win 1 of 4 £10 vouchers?

If 'Yes', please enter email address below.

◯ Yes
◯ No

Would you be willing to be contacted by the research team to be invited to join or comment on other parts of this study and other relevant research projects?

If 'Yes', please enter email address below.

◯ Yes
◯ No

I wish to be contacted by email by the research team about the results of this study.

If 'Yes', please enter email address below.

◯ Yes
◯ No

Please enter you email address: ________________________________

You may be aware that from 25th May 2018, the law changed regarding how we can handle your personal information. To give you assurance that we have dealt with these changes appropriately, you can find out more about how we use your information at https://www.liverpool.ac.uk/legal/data_protection/

◯ If you are willing to participate, please click this box.

◯ I wish to complete only the Induction of Labour Survey
◯ I wish to complete only the Caesarean Survey
◯ I wish to complete the combined Induction of Labour and Caesarean Survey

Thank you for agreeing to take part in this study. We are interested in what you think is important to discuss when planning to have a vaginal birth. Please answer all of the questions. The information that you provide will remain anonymous.

Development of a Core Information Set for Induction of Labour

Development of a Core Information Set for Caesarean

Development of a Core Information Set for Induction of Labour & Caesarean

First, a few things about you.
To allow us to analyse the results of the study, we need some brief information about you and your expertise.

Please select the most appropriate answers.

Are you a:

- ◯ Pregnant person
- ◯ Planning a pregnancy
- ◯ Recently had a baby
- ◯ Partner
- ◯ Medico-legal expert
- ◯ Midwife
- ◯ Maternity Care Assistant / Health Care Assistant
- ◯ Obstetrics and Gynaecology Consultant
- ◯ Obstetrics and Gynaecology trainee
- ◯ Obstetrics and Gynaecology SHO
- ◯ GP
- ◯ Anaesthetic Consultant
- ◯ Anaesthetic trainee / Speciality doctor
- ◯ Operating Department Practitioners (ODPs)
- ◯ Nurse
- ◯ Physiotherapist
- ◯ Other healthcare professional
- ◯ Member of an interested organisation

Ethnicity:

- ◯ White British
- ◯ White Other
- ◯ Mixed/ Multiple ethnic group
- ◯ Asian/ Asian British
- ◯ Black/ African/ Caribbean/ Black British
- ◯ Other ethnic groups
- ◯ Prefer not to say

How many weeks pregnant are you?

◯ 0
◯ 1
◯ 2
◯ 3
◯ 4
◯ 5
◯ 6
◯ 7
◯ 8
◯ 9
◯ 10
◯ 11
◯ 12
◯ 13
◯ 14
◯ 15
◯ 16
◯ 17
◯ 18
◯ 19
◯ 20
◯ 21
◯ 22
◯ 23
◯ 24
◯ 25
◯ 26
◯ 27
◯ 28
◯ 29
◯ 30
◯ 31
◯ 32
◯ 33
◯ 34
◯ 35
◯ 36
◯ 37
◯ 38
◯ 39
◯ 40
◯ 41
◯ 42

Please state which one: ______________________________

Where do you practice?

◯ Hospital
◯ Community
◯ Midwife led centre
◯ Other

Where do you practice?

◯ Hospital
◯ Community
◯ Other

Please specify: ______________________________

How many years have you held this position?

◯ Less than 5
◯ 5-10
◯ 11-20
◯ More than 20

Region of residence:

◯ North West
◯ North East
◯ Yorkshire and the Humber
◯ East Midlands
◯ West Midlands
◯ East England
◯ London
◯ South East
◯ South West
◯ Scotland
◯ Wales
◯ Northern Ireland
◯ Other

Please specify: __________________________________

Your age:

◯ Under 21
◯ 21-30
◯ 31-40
◯ 41-50
◯ 51-60
◯ 61-70
◯ 71-80
◯ Over 80

Your sex:

◯ Male
◯ Female
◯ Prefer not to say

Is the gender you identify with the same as your sex registered at birth?

◯ Yes
◯ No
◯ Prefer not to say

Enter gender identity: __________________________________

How many children do you have?

◯ Currently planning a pregnancy
◯ 1
◯ 2
◯ 3
◯ 4
◯ 5
◯ 6
◯ 7
◯ 8
◯ 9
◯ 10
◯ Other

Please specify:

____

If you have previously given birth, what type of birth have you / your partner previously had or experienced?

☐ Spontaneous vaginal birth
☐ Forceps birth
☐ Ventouse / Kiwi / Silastic birth
☐ Emergency caesarean section
☐ Elective caesarean section
☐ Not applicable

Please use this box to write down the key information that you think are absolutely crucial to discuss with a woman / birthing person before induction of labour is chosen.

Please use this box to write down the key information that you think are absolutely crucial to discuss with a woman / birthing person before caesarean is chosen.

Thank you for completing this survey.
