# Supplementary material for: What should be discussed when considering an induction of labour? A UK-wide, multi-centre Delphi study to develop a core information set for induction of labour
Source: BMJ Open. 2026 May 27;16(5):e118024. doi: 10.1136/bmjopen-2026-118024 (PMC13218194; doi:10.1136/bmjopen-2026-118024)
Supplement: online supplemental file 6 [file bmjopen-16-5-s006.pdf]

Start time: [survey-time-started:Round 1 Induction of Labour Delphi Consensus Survey]
End time: [survey-time-completed:Round 1 Induction of Labour Delphi Consensus Survey]

Round 1 Induction of Labour Delphi Consensus Survey

Thank you for taking the time to look at our survey.

This is one part of a study which is looking at understanding what is the important information women/birthing people need when making decisions about whether to have an induction of labour. As part of this we wanted to incorporate the views and experiences of as many different people possible.

We are therefore inviting women/birthing parents who are either currently pregnant, planning a pregnancy or who have had a baby recently, healthcare professionals who work with pregnant/postnatal women/birthing parents, medico-legal experts and individuals who are part of groups with an interest in maternity care/ birthing rights to complete a survey in which they score the importance of individual information points relating to induction of labour. There will be 2 rounds of the survey, open for at least 2 weeks, taking place approximately one month apart. Each round of the survey should take no more than 20 minutes of your time.

You will not be identifiable in your answers. To participate in the Delphi you will need to leave us your email address which will be deleted when the study is completed. This is so we can send you a personalised link to the second round of the survey. You will also be invited to be entered into a prize draw for a £25 voucher (4 available). We will also ask if you would like to receive the results of this study, or if you are willing to be contacted by the research team to take part in later parts of this study or other relevant research projects. If you answer 'yes' to any of these, we ask that you leave your email address.

Your participation is entirely voluntary, and you can stop completing the questionnaire at any time and you can withdraw your participation from the study at any time for 2 weeks after you complete the questionnaire, as long as you have left us your email address.

For further information about the study as a whole please click here, and for more in depth participant information please click the document link below.

If you have any concerns to raise about the conduct of this study, please contact sponsor@liverpool.ac.uk

If you have any queries about the study, please email options@liverpool.ac.uk

[Attachment: "Maternity CIS_PIS_Delphi V1.1_28032023 Induction.pdf"]

Am I eligible to participate? You can take part if you are:

- Pregnant, planning pregnancy or have had a baby
- A partner of someone who is pregnant, planning pregnancy or has had a baby
- A healthcare professional
- For example, obstetricians, midwives, midwifery care assistants, anaesthetists, general practitioners, physiotherapists who care for people who are pregnant, are in labour, or have experienced induction of labour
- A medico-legal expert
- Legal professionals with a specialist interest in childbirth
- A member of an interested organisation
- Someone working for a charity or group that is interested in or advocating for birthing rights

How will my data be used and where can I get more information about the research?
All the information you give us will be strictly confidential.

When you agree to take part you will be given an ID number and this is the number that will be used on your records.

All the information that we get from you is anonymised and securely stored at the University of Liverpool, according to the University of Liverpool storage policies.

You will not be able to be identified from the information that is stored.

Consent to take part in the survey By taking part in this survey you are agreeing for the anonymous answers you give to be used in our analyses.

If you agree, we will ask you for your email address so that we can email you a link with the second questionnaire.

All reports and publications will not have any individual information, and will be anonymous.

Would you like to be entered into a prize draw for a chance to win 1 of 4 £25 vouchers?	◯ Yes<br>◯ No
Would you be willing to be contacted by the research team to be invited to join or comment on other parts of this study and other relevant research projects?	◯ Yes<br>◯ No
I wish to be contacted by email by the research team about the results of this study.	◯ Yes<br>◯ No
I am willing to participate in this survey.	◯ Yes

Please provide your email address so that we may contact you ______

______ ______

____

What is a 'core information set'?

What is a 'core information set'?

A core information set is an agreed standardised set of information that should be discussed prior to going through an experience or procedure, as a minimum, with ALL women/birthing people considering having induction of labour.

The aim of this study is to identify the most important information points for those considering induction of labour.

This is so that the information discussed prior to induction of labour covers the information women/birthing people need to know to ensure they have the information they need to make their decision.

This core information set can then be used to guide conversations with people planning on having an induction of labour, as well as being used in patient information leaflets and literature on the subject, all with the aim of improving information about birth choices.

What is an 'information point'?

An information point is something relating to the experience someone may go through when having induction of labour. This could be either during the birth, or following birth. This could be related to mental or physical health, or subsequent pregnancy.

For example:

Were there any medical complications after giving birth?

How does havinginduction of labour affect life afterwards?

How do I complete the survey?

- Each information item is stated in bold at the beginning of each item followed by an explanation. This allows us to use the same questionnaire for parents, healthcare professionals, researchers, medico-legal experts and charity representatives.

- We would like you to score the importance of each item on a scale from one to nine.

- Please indicate if you think an item is of limited importance (score 1 to 3), important but not critical (score 4 to 6), or critical (score 7 to 9).

- You should only rate an item as 7-9 if you think it is important to tell all women planning induction of labour.

- It is important that you try to use the full range of the scale when selecting your answer.

- If you do not have a view or do not wish to answer any questions, please indicate 'unable to rate'.

- Please keep in mind you are answering about the care of all people who have experienced, or may experience, induction of labour.

Example question...

How important is it for all pregnant women/birthing parents planning or considering an induction of labour to know about...?

	1	Limited importance 2	3	4	Important not critical5	6	7	Critical8	9	Unable to rate
Options if they change their mind about induction of labour?	○	○	○	○	○	○	○	○	○	○

Survey sections

The survey covers information points under the following 10 section headings:

- Reasons for being offered an induction of labour
- Methods of induction of labour
- Induction of labour process
- Risks and benefits of induction of labour
- Monitoring during induction of labour
- Outcome of induction of labour
- Pain management
- Practicalities of induction of labour
- Induction of labour and the NHS
- Decision making of induction of labour This survey should take approximately 20 minutes to complete.

First, a few things about you...

To allow us to analyse the results of the study, we need some brief information about you and your expertise. Please select the most appropriate answers.

Are you a?
(please tick all that apply to you)

☐ Person planning a pregnancy
☐ Person who is currently pregnant
☐ Person who has given birth at some point in the past
☐ Partner of someone who is currently pregnant, or has given birth in the past
☐ Person who works in the area of vaginal birth in a professional, voluntary or research capacity

---

What is your role?
(please tick all that apply)

☐ Midwife
☐ Midwifery care assistant
☐ Obstetrics and gynaecology doctor
☐ Anaesthetist
☐ General practitioner
☐ Operating department practitioner (ODP)
☐ Physiotherapist
☐ Member of an interested organisation or charity
☐ Medico-legal professional
☐ Researcher
☐ Other

What organisation are you part of?

____

How many years have you been part of this organisation?

◯ Fewer than 5
◯ 5-10
◯ 11-20
◯ More than 20

If Other, what is your role?

____

How many years have you been in this role?

◯ Fewer than 5
◯ 5-10
◯ 11-20
◯ More than 20

If medico-legal professional, what is your role?

____

How many years have you been in this role?

◯ Fewer than 5
◯ 5-10
◯ 11-20
◯ More than 20

If research professional, what is your role?

____

How many years have you been in this role?

◯ Fewer than 5
◯ 5-10
◯ 11-20
◯ More than 20

How many years have you been an ODP?

◯ Fewer than 5
◯ 5-10
◯ 11-20
◯ More than 20

How many years have you been a physiotherapist?

◯ Fewer than 5
◯ 5-10
◯ 11-20
◯ More than 20

How many years have you been a midwife?

◯ Fewer than 5
◯ 5-10
◯ 11-20
◯ More than 20

What is your current role as a midwife?

◯ Trainee midwife
◯ Band 5
◯ Band 6
◯ Band 7
◯ Band 8
◯ Other

If Other, what is your role?

____

How many years have you been an obstetrics &/or gynaecology doctor?

◯ Fewer than 5 years
◯ 5-10
◯ 11-20
◯ More than 20

What is your current role?

◯ Trainee
◯ Clinical fellow
◯ Specialty doctor
◯ Consultant
◯ Other

If Other, what is your role?

____

How many years have you been an anaesthetic doctor?

◯ Fewer than 5
◯ 5-10
◯ 11-20
◯ More than 20

What is your current role?

◯ Trainee
◯ Clinical fellow
◯ Specialty doctor
◯ Consultant
◯ Other

If Other, what is your role?

____

How many years have you been a GP?

◯ Fewer than 5 years
◯ 5-10
◯ 11-20
◯ More than 20

What is your current role?

◯ Trainee
◯ Qualified GP
◯ Other

If Other, what is your role?

____

How many weeks pregnant are you currently?	◯ 4<br>◯ 5<br>◯ 6<br>◯ 7<br>◯ 8<br>◯ 9<br>◯ 10<br>◯ 11<br>◯ 12<br>◯ 13<br>◯ 14<br>◯ 15<br>◯ 16<br>◯ 17<br>◯ 18<br>◯ 19<br>◯ 20<br>◯ 21<br>◯ 22<br>◯ 23<br>◯ 24<br>◯ 25<br>◯ 26<br>◯ 27<br>◯ 28<br>◯ 29<br>◯ 30<br>◯ 31<br>◯ 32<br>◯ 33<br>◯ 34<br>◯ 35<br>◯ 36<br>◯ 37<br>◯ 38<br>◯ 39<br>◯ 40+<br>◯ Prefer not to say
How many children do you have?	◯ 0<br>◯ 1<br>◯ 2<br>◯ 3<br>◯ 4<br>◯ 5<br>◯ 6<br>◯ 7<br>◯ 8<br>◯ 9<br>◯ 10<br>◯ More than 10<br>◯ Prefer not to say

How long ago did your (most recent) birth occur?

- ◯ In the last 6 months
- ◯ In the last year
- ◯ 1
- ◯ 2
- ◯ 3
- ◯ 4
- ◯ 5
- ◯ 6
- ◯ 7
- ◯ 8
- ◯ 9
- ◯ 10
- ◯ 11
- ◯ 12
- ◯ 13
- ◯ 14
- ◯ 15
- ◯ 16
- ◯ 17
- ◯ 18
- ◯ 19
- ◯ 20
- ◯ More than 20 years ago

If you have previously given birth, what type of birth(s) have you experienced?
(please tick all that apply)

- ☐ Vaginal birth
- ☐ Instrumental birth (forceps, ventouse, kiwi, silastic)
- ☐ Emergency caesarean section
- ☐ Elective caesarean section
- ☐ Prefer not to say

What is your current employment status?

- ◯ Employed full time/On maternity leave
- ◯ Employed part time/On maternity leave
- ◯ Not currently employed
- ◯ Homemaker
- ◯ Retired
- ◯ Student
- ◯ Other
- ◯ Prefer not to say

Current employment status

____

What is your highest level of education?

- ◯ Pre-GCSEs or equivalent
- ◯ GCSEs or equivalent
- ◯ A-levels or equivalent
- ◯ Bachelors degree or equivalent
- ◯ Post-graduate degree
- ◯ Other
- ◯ Prefer not to say

What is your highest level of education?

____

Area of residence

◯ East of England
◯ London
◯ Midlands
◯ North East England and Yorkshire
◯ North West England
◯ Northern Ireland
◯ Scotland
◯ South East England
◯ South West England
◯ Wales
◯ Other

Area of work

◯ East of England
◯ London
◯ Midlands
◯ North East England and Yorkshire
◯ North West England
◯ Northern Ireland
◯ Scotland
◯ South East England
◯ South West England
◯ Wales
◯ Other

If Other, what is your area of residence

____

If Other, what is your area of work

____

How old are you?

◯ Under 21
◯ 21-30
◯ 31-40
◯ 41-50
◯ 51-60
◯ 61-70
◯ 71-80
◯ Over 80
◯ Prefer not to say

Your gender?

◯ Female
◯ Male
◯ Prefer not to say

Is the gender you identify with the same as your sex registered at birth?

◯ Yes
◯ No
◯ Prefer not to say

Enter gender identity

____

Ethnicity

◯ White British
◯ White Other
◯ Mixed/multiple ethnic groups
◯ Asian/Asian British
◯ Black/African/Caribbean/Black British
◯ Other ethnic groups
◯ Prefer not to say

Ethnicity

____

Your local maternity care organisation is:

◯ North Bristol NHS Trust
◯ Royal United Hospitals Bath NHS Foundation Trust
◯ Cambridge University Hospitals NHS Foundation Trust
◯ Liverpool Women's NHS Foundation Trust
◯ The Shrewsbury and Telford Hospital NHS Trust
◯ University College Hospitals (London) NHS Foundation Trust
◯ NHS Grampian
◯ Other

If other, please detail here

____

Survey Progress: 8%

Section 1: Reasons for being offered induction of labour

How important is it for all pregnant women/birthing parents planning or considering an induction of labour to know about...?

	1	Limited importance 2	3	4	Important not critical5	6	7	Critical8	9	Unable to rate
How common induction of labour is	◯	◯	◯	◯	◯	◯	◯	◯	◯	◯

	1	Limited importa nce 2	3	4	Importa nt not critical5	6	7	Critical8	9	Unable to rate
Reasons that induction of labour could be planned<br>e.g. Diabetes, aged 40+, IVF pregnancy, multiple pregnancies, chronic conditions, personal request.	○	○	○	○	○	○	○	○	○	○

	1	Limited importa nce 2	3	4	Importa nt not critical5	6	7	Critical8	9	Unable to rate
When induction can be offered for example post-dates, at certain times for some conditions (diabetes, twins)	○	○	○	○	○	○	○	○	○	○

	1	Limited importa nce 2	3	4	Importa nt not critical5	6	7	Critical8	9	Unable to rate
Unexpected reasons induction of labour could be offered<br>e.g. pre-labour rupture of membranes, concerns with mum's health, obstetric cholestasis, infection, bleeding, pre-eclampsia, severe maternal illness, small/large baby, growth problems for baby, post-term	○	○	○	○	○	○	○	○	○	○

Survey Progress: 16%

Section 2: Methods of induction of labour

How important is it for all pregnant women/birthing parents planning or considering induction of labour to know...?

	1	Limited importance 2	3	4	Important not critical5	6	7	Critical8	9	Unable to rate
The role of membrane sweeping to ripen the cervix	○	○	○	○	○	○	○	○	○	○

	1	Limited importance 2	3	4	Important not critical5	6	7	Critical8	9	Unable to rate
Methods to begin induction of labour by ripening the cervix, including medical (propess, prostin, misoprostol) and mechanical methods (balloon catheter, rods)	○	○	○	○	○	○	○	○	○	○

	1	Limited importance 2	3	4	Important not critical5	6	7	Critical8	9	Unable to rate
Methods of inducing contractions including artificial rupture of membranes and the hormone drip	○	○	○	○	○	○	○	○	○	○

	1	Limited importance 2	3	4	Important not critical5	6	7	Critical8	9	Unable to rate
The evidence to support/dispute complementary therapies that birthing people may try e.g. acupuncture, homeopathy, nipple stimulation, castor oil, massaging breasts, raspberry leaf tea, curry, fresh pineapple, eating 6 dates a day, reflexology, aromatherapy, sexual intercourse, keep active-walking, primrose oil, vaginal douching prior to induction	○	○	○	○	○	○	○	○	○	○

Survey Progress: 24%

Section 3: Induction of labour process

How important is it for all pregnant women/birthing parents planning or considering induction of labour to know...?

	1	Limited importa nce 2	3	4	Importa nt not critical5	6	7	Critical8	9	Unable to rate
The possible locations of inductions e.g. at home or in hospital	○	○	○	○	○	○	○	○	○	○

	1	Limited importa nce 2	3	4	Importa nt not critical5	6	7	Critical8	9	Unable to rate
Care during induction of labour e.g. during ripening phase and labour, how often will be checked (vaginal examinations)	○	○	○	○	○	○	○	○	○	○

	1	Limited importa nce 2	3	4	Importa nt not critical5	6	7	Critical8	9	Unable to rate
Induction urgency levels and its impact on the length of the process	○	○	○	○	○	○	○	○	○	○

	1	Limited importa nce 2	3	4	Importa nt not critical5	6	7	Critical8	9	Unable to rate
Factors affecting whether induction leads to active labour e.g. how far a long pregnancy is, how 'ripe' cervix is at the start of induction	○	○	○	○	○	○	○	○	○	○

	1	Limited importance 2	3	4	Important not critical5	6	7	Critical8	9	Unable to rate
Potential variation in length of induction of labour	○	○	○	○	○	○	○	○	○	○

	1	Limited importance 2	3	4	Important not critical5	6	7	Critical8	9	Unable to rate
Ability to speed up/slow down process of contractions, and the pro's and con's of doing this	○	○	○	○	○	○	○	○	○	○

	1	Limited importance 2	3	4	Important not critical5	6	7	Critical8	9	Unable to rate
Birth of the placenta and reducing bleeding after birth (management of the third stage)	○	○	○	○	○	○	○	○	○	○

	1	Limited importance 2	3	4	Important not critical5	6	7	Critical8	9	Unable to rate
Information regarding partners, their presence and role during the birth.	○	○	○	○	○	○	○	○	○	○

Survey Progress: 32%

Section 4: Risks and benefits of induction

How important is it for all pregnant women/birthing parents planning or considering an induction of labour to know about...?

	1	Limited importance 2	3	4	Important not critical5	6	7	Critical8	9	Unable to rate

Considerations with induction for the mother<br>e.g. may not work, may take several days work, may involve more vaginal exams, place of birth may need to change from original plan, may not be able to use pool	○	○	○	○	○	○	○	○	○	○
	1	Limited importa nce 2	3	4	Importa nt not critical5	6	7	Critical8	9	Unable to rate
Possible risks for the mother with induction of labour<br>e.g. increased risk of bleeding after birth, infection in the lining of the womb, need for antibiotics	○	○	○	○	○	○	○	○	○	○
	1	Limited importa nce 2	3	4	Importa nt not critical5	6	7	Critical8	9	Unable to rate
Possible risks with induction of labour for the baby<br>e.g. fetal distress due to no rest between contractions, changes in babies heart rate	○	○	○	○	○	○	○	○	○	○
	1	Limited importa nce 2	3	4	Importa nt not critical5	6	7	Critical8	9	Unable to rate
Benefits of induction for mother<br>e.g. reduce risk of caesarean birth, give birth earlier, planned date for start of induction, less anxiety	○	○	○	○	○	○	○	○	○	○

	1	Limited importa nce 2	3	4	Importa nt not critical5	6	7	Critical8	9	Unable to rate
Benefits of induction for the baby<br>e.g. reduce risks of stillbirth, reduce risks of infection (in some situations)	○	○	○	○	○	○	○	○	○	○

	1	Limited importa nce 2	3	4	Importa nt not critical5	6	7	Critical8	9	Unable to rate
The different risks associated with cervical ripening. Including the side effects of the specific medical and mechanical methods used<br>e.g. nausea, diarrhoea, vaginal soreness	○	○	○	○	○	○	○	○	○	○

	1	Limited importa nce 2	3	4	Importa nt not critical5	6	7	Critical8	9	Unable to rate
The different risks associated with induction of contractions<br>e.g. uterus contracting too frequently	○	○	○	○	○	○	○	○	○	○

	1	Limited importa nce 2	3	4	Importa nt not critical5	6	7	Critical8	9	Unable to rate
Red flags to alert midwifery/medical staff to during the induction<br>e.g. pain, bleeding, reduced fetal movements	○	○	○	○	○	○	○	○	○	○

	1	Limited importance 2	3	4	Important not critical5	6	7	Critical8	9	Unable to rate
Specifically important risk situations<br>e.g. Previous caesarean section, small baby, BMI.	○	○	○	○	○	○	○	○	○	○

Survey Progress: 40%

Section 5: Monitoring during induction of labour

How important is it for all pregnant women/birthing parents planning or considering an induction of labour to know about...?

	1	Limited importance 2	3	4	Important not critical5	6	7	Critical8	9	Unable to rate
How they will be monitored during induction<br>e.g. vital signs (temperature, pulse, respiration, blood pressure), differences between cervical ripening and induction of contractions	○	○	○	○	○	○	○	○	○	○

	1	Limited importance 2	3	4	Important not critical5	6	7	Critical8	9	Unable to rate
How their baby will be monitored during induction<br>e.g. baby heart trace, differences between cervical ripening and induction of contractions	○	○	○	○	○	○	○	○	○	○

Survey Progress: 48%

Section 6: Outcomes of induction of labour   How important is it for all pregnant women/birthing parents planning or considering an induction of labour to know about...?

	1	Limited importance 2	3	4	Important not critical5	6	7	Critical8	9	Unable to rate
Possible emergency situations e.g. Cord prolapse, caesarean section, shoulders getting stuck at birth.	○	○	○	○	○	○	○	○	○	○

	1	Limited importance 2	3	4	Important not critical5	6	7	Critical8	9	Unable to rate
What happens if active labour does not commence<br>e.g. resting, starting again, a different method or caesarean birth	○	○	○	○	○	○	○	○	○	○

	1	Limited importance 2	3	4	Important not critical5	6	7	Critical8	9	Unable to rate
We may not know why active labour does not commence for you	○	○	○	○	○	○	○	○	○	○

	1	Limited importance 2	3	4	Important not critical5	6	7	Critical8	9	Unable to rate
The general facts about how many women/birthing people having an induction of labour have spontaneous vaginal birth, assisted vaginal birth and emergency caesarean birth	○	○	○	○	○	○	○	○	○	○

	1	Limited importance 2	3	4	Important not critical5	6	7	Critical8	9	Unable to rate

Postnatal issues following induction<br>e.g. mental and physical health	○	○	○	○	○	○	○	○	○	○

Survey Progress: 56%

Section 7: Pain management

How important is it for all pregnant women/birthing parents planning or considering an induction of labour to know the...?

	1	Limited importance 2	3	4	Important not critical5	6	7	Critical8	9	Unable to rate
Different pharmacological pain relief options available<br>e.g. Gas & air (Entonox), Oral (paracetamol/dihydrocodeine), Injected (morphine/pethidine), Remifentanyl patient controlled analgesia, Epidural)	○	○	○	○	○	○	○	○	○	○

	1	Limited importance 2	3	4	Important not critical5	6	7	Critical8	9	Unable to rate
Different non-pharmacological pain relief options available<br>e.g. TENS, water, water injections	○	○	○	○	○	○	○	○	○	○

	1	Limited importance 2	3	4	Important not critical5	6	7	Critical8	9	Unable to rate
Impact of induction of labour on pain experienced	○	○	○	○	○	○	○	○	○	○

Survey Progress: 64%

Section 8: Practicalities of induction of labour

How important is it for all pregnant women/birthing parents planning or considering an induction of labour to know about...?

	1	Limited importa nce 2	3	4	Importa nt not critical5	6	7	Critical8	9	Unable to rate
Eating and drinking during the induction process	○	○	○	○	○	○	○	○	○	○

	1	Limited importa nce 2	3	4	Importa nt not critical5	6	7	Critical8	9	Unable to rate
Moving around during induction e.g. birthing balls, showers & baths	○	○	○	○	○	○	○	○	○	○

	1	Limited importa nce 2	3	4	Importa nt not critical5	6	7	Critical8	9	Unable to rate
What happens on the day	○	○	○	○	○	○	○	○	○	○

Survey Progress: 72%

Section 9: Induction of labour and the NHS

How important is it for all pregnant women/birthing parents planning or considering an induction of labour to know about...?

	1	Limited importa nce 2	3	4	Importa nt not critical5	6	7	Critical8	9	Unable to rate
The financial costs to the NHS for induction of labour	○	○	○	○	○	○	○	○	○	○

Survey Progress: 80%

Section 10: Decision making about induction of labour

How important is it for all pregnant women/birthing parents planning or considering an induction of labour to know about...?

	1	Limited importa nce 2	3	4	Importa nt not critical5	6	7	Critical8	9	Unable to rate
What could happen if you do nothing and decide to wait for labour to come naturally<br>e.g. stillbirth, not going into labour naturally despite waiting	○	○	○	○	○	○	○	○	○	○

	1	Limited importa nce 2	3	4	Importa nt not critical5	6	7	Critical8	9	Unable to rate
Decision making for induction<br>e.g. choice lies with the woman/birthing person, offering of alternative options, ability to say no	○	○	○	○	○	○	○	○	○	○

	1	Limited importa nce 2	3	4	Importa nt not critical5	6	7	Critical8	9	Unable to rate
Maternal satisfaction with induction of labour	○	○	○	○	○	○	○	○	○	○

	1	Limited importa nce 2	3	4	Importa nt not critical5	6	7	Critical8	9	Unable to rate
Information on the concerns people might have<br>e.g. fear of delay, being alone, having a feeling of time being up	○	○	○	○	○	○	○	○	○	○

	1	Limited importa nce 2	3	4	Importa nt not critical5	6	7	Critical8	9	Unable to rate
Impact on future pregnancies	○	○	○	○	○	○	○	○	○	○

	1	Limited importa nce 2	3	4	Importa nt not critical5	6	7	Critical8	9	Unable to rate
Comparison of planned caesarean section versus induction of labour	○	○	○	○	○	○	○	○	○	○

	1	Limited importa nce 2	3	4	Importa nt not critical5	6	7	Critical8	9	Unable to rate
Having a structured way of approaching decisions e.g. the 'BRAIN' tool (benefits, risks, alternatives, intuition, do nothing)	○	○	○	○	○	○	○	○	○	○

Survey Progress: 88%

Additional Information

Is there any additional information you would like to know about induction of labour birth? __________________________________

Survey Progress: 100%
