# Supplementary material for: What should be discussed when considering an induction of labour? A UK-wide, multi-centre Delphi study to develop a core information set for induction of labour
Source: BMJ Open. 2026 May 27;16(5):e118024. doi: 10.1136/bmjopen-2026-118024 (PMC13218194; doi:10.1136/bmjopen-2026-118024)
Supplement: online supplemental file 7 [file bmjopen-16-5-s007.pdf]

Induction of labour Core Information Set

Birth Options

What is a core information set?

A core information set is the information everyone needs before making a decision about their care. They do not replace personalised discussions. The Birth Options core information sets have been made for families and healthcare professionals to use to provide information to support decisions about birth. Women, birthing people, partners, midwives and doctors have decided which information is most important.

This information is intended as a guide and uses evidence from national guidelines, national statistics and research studies. It includes some more general information that explains usual practice. It has the best available information at the time that it was made (2025).

This is the birth options core information set for induction of labour

It includes information about the process of **induction of labour**.

It does not include extensive information about spontaneous vaginal birth, instrumental vaginal birth, or caesarean birth.

Other Core Information Sets are available for **vaginal birth, planned/unplanned caesarean birth, emergency caesarean birth, post caesarean, and will soon be available for instrumental birth**.

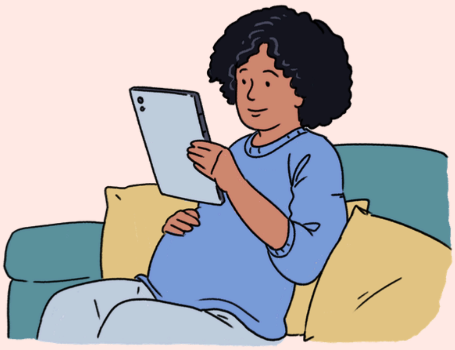

www.birthoptions.co.uk

FUNDED BY NIHR National Institute for Health and Care Research

University of Liverpool

NHS Liverpool Women's NHS Foundation Trust

When and why an induction of labour may be offered

Induction of labour means any intervention encouraging labour to begin when it hasn't started naturally.

Each pregnancy is different, you can discuss what's best for you based on your health and your baby's wellbeing with your midwife/doctor.

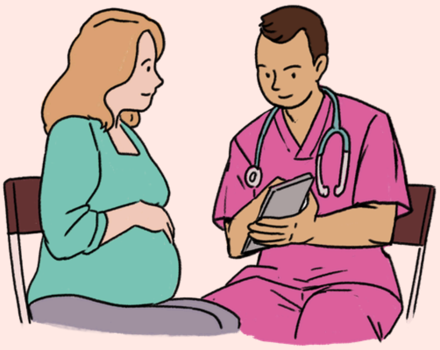

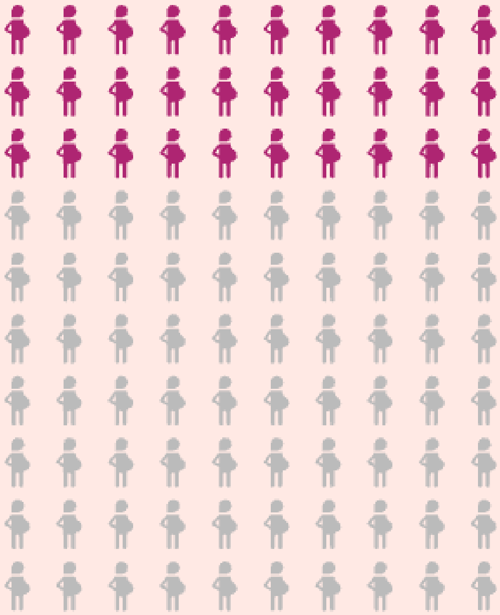

Induction of labour is common.

In the UK, about **30 in 100** women have an induction of labour.

You might be offered induction if:

1. You're overdue (past 41 weeks)
2. Your waters break before labour
3. You have certain health conditions
4. Concerns about your baby's health
5. You are expecting more than one baby
6. You're 40 years old or more
7. You request it

FUNDED BY NIHR | National Institute for Health and Care Research

University of Liverpool

NHS Liverpool Women's NHS Foundation Trust

When and why an induction of labour may be offered

Birth Options

After your due date:

Weeks of Pregnancy	Proportion of Spontaneous Labours Started by this Gestational Age	Obstetric Care: what to expect at this point in pregnancy
At 41 weeks (1 week overdue)	**83 in 100** women/birthing people<br>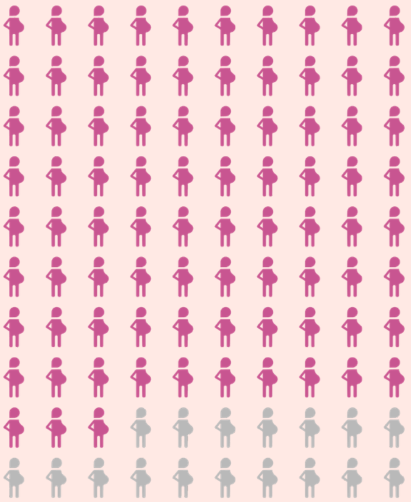	Your team will offer to plan a date for induction. This is due to increasing chances of:<br>• Caesarean birth<br>• Stillbirth<br>• Neonatal admission<br>• Neonatal death<br>You can choose to wait for spontaneous labour if you wish.
Up to 42 weeks	**99 in 100** women/birthing people<br>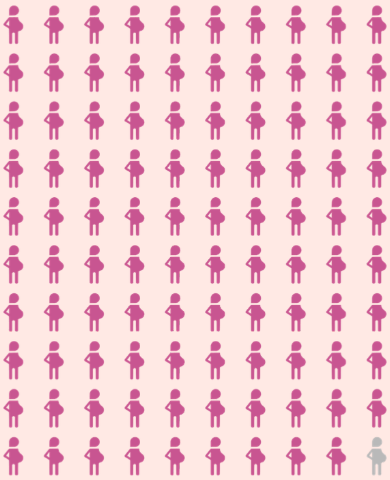	If you wish to wait for spontaneous labour beyond 42 weeks – extra monitoring may be offered, but it cannot always predict or prevent stillbirth or other complications.

FUNDED BY NIHR | National Institute for Health and Care Research
UNIVERSITY OF LIVERPOOL
NHS Liverpool Women's NHS Foundation Trust

Options for starting an induction of labour

There are different ways of starting labour depending on the readiness of the neck of your womb (cervix), your pregnancy history and what is available at your hospital.

Before an induction, a stretch and sweep (membrane sweep) can be offered which involves using a finger to separate the membranes around the baby from the neck of the womb (cervix). This is usually offered after 39 weeks of pregnancy, but can be offered earlier.

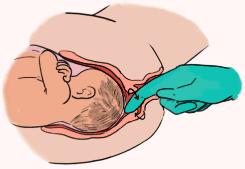

Membrane Sweep

Medication can be placed inside your vagina or cervix, or a device (either a balloon or dilators) can be placed inside the neck of your womb (cervix). This is called cervical ripening. You may have the option to go home or stay in hospital. Talk to your midwife or doctor about this.

Cervical ripening

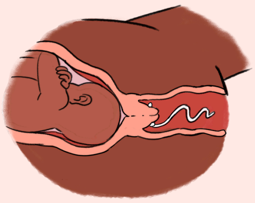

Prostaglandin pessary

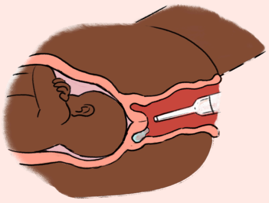

Prostaglandin gel

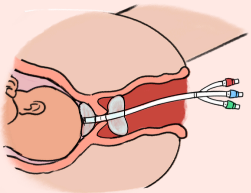

Balloon dilator

These methods can take up to 24 hours. If they do not work the first time, you may need to try the same or another method. Talk to your midwife or doctor about what is available at your trust and what the best option is for you.

Once the neck of your womb (cervix) is open enough, your midwife/doctor will offer to break your waters (amniotomy/artificial rupture of membranes). This may start contractions.

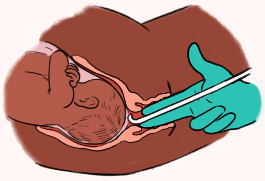

Breaking your waters (amniotomy/artificial rupture of membranes)

If contractions do not start, your midwife/doctor will recommend the hormone drip (oxytocin) to speed up labour and reduce the risk of infection.

This acts like the natural oxytocin in your body.

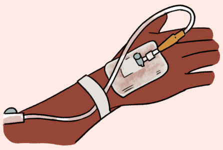

Cannula

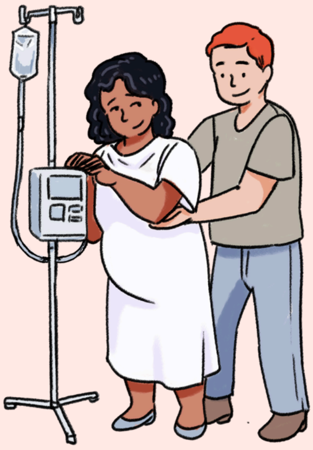

Oxytocin drip

FUNDED BY NIHR | National Institute for Health and Care Research
University of Liverpool
NHS Liverpool Women's NHS Foundation Trust

If labour does not commence despite being induced, a doctor will discuss with you what your options are going forward.

Depending on your specific case they may offer you:

1. Further attempt at inducing labour using a different method.
2. A period of rest and then revisit your options.
3. Seeing if you go into active labour on your own.
4. A Caesarean birth.

Alternative therapies and methods of starting labour

You may want to try alternative methods for inducing labour. Some of the commonly mentioned methods are listed below:

- Herbal Supplements
- Acupuncture/Acupressure
- Homeopathy
- Castor Oil, Hot Baths, and Enemas
- Sexual Intercourse
- Breast/Nipple Stimulation
- Hypnosis
- Raspberry Leaf Tea
- Curry (Spicy Foods)
- Aromatherapy
- Walking
- Vaginal Douching
- Reflexology
- Evening Primrose Oil
- Eating Dates
- Fresh Pineapple

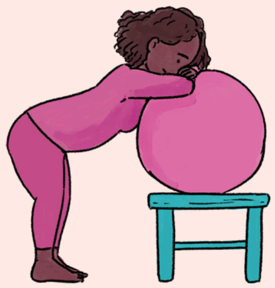

There is no evidence that these methods work.

Supporting you to make an informed decision about induction of labour

The “BRAIN” decision-making tool may help in deciding about induction of labour.

1. **B**enefits – What are the benefits?
2. **R**isks – What are the risks?
3. **A**lternatives – What are the alternatives?
4. **I**ntuition – How do you feel? What is your gut feeling?
5. **N**othing – Do nothing, wait for labour to start.

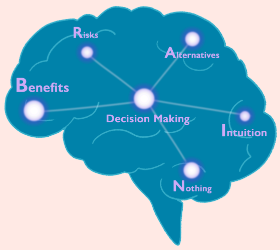

B.R.A.I.N

The decision to have an induction of labour is entirely up to you.

FUNDED BY NIHR | National Institute for Health and Care Research
University of Liverpool
NHS Liverpool Women's NHS Foundation Trust

Benefits of Induction of Labour

Birth Options

Benefits to mother:

- Feeling an increased sense of control when planning your birth.
- Reduces your chance of developing late onset pre-eclampsia (>39 weeks).
- Reduces your chance of an unplanned/emergency caesarean birth by approximately 10% compared to waiting for your labour to start until 41 weeks.

Benefits to baby:

- Baby is more likely to be born with a better APGAR score (quick health check of baby).
- Reduces the likelihood of stillbirth late in pregnancy.
- Reduces the risk of your baby needing admission to the neonatal intensive care unit (NICU).
- Reduces the likelihood of neonatal death.
- If you've been told that your baby is large-for-dates, the chances of their shoulders getting stuck at the time of birth (shoulder dystocia) may be reduced.

Inducing labour prevents one perinatal death for roughly every 544 women who have it. Perinatal death means inside the womb and up to first week of life.

Benefits of oxytocin use:

- Is generally considered safe for mother and baby.
- It has not been shown to increase the uptake of epidurals.

Benefits in specific circumstances:

- If you have had a previous caesarean birth, mechanical methods (using a foley or Cook's catheter) are associated with a lower risk of the scar on your womb coming apart (rupturing).
- Induction may be more beneficial for those whom stillbirth rates are higher for example; Those from a minority ethnic background, those who live in more deprived areas of the UK or your baby has fetal growth restriction.
- Women with a BMI above 30kg/m2 may have a reduced chance of an unplanned/ emergency caesarean birth if they are induced between 39-40 weeks compared to waiting for spontaneous labour to begin.

FUNDED BY NIHR | National Institute for Health and Care Research
UNIVERSITY OF LIVERPOOL
NHS Liverpool Women's NHS Foundation Trust

Risks of Induction of Labour for pregnant women/birthing parents and baby

Birth Options

Key considerations for mothers:

- Induction is a medical intervention with associated monitoring and examinations.
- Induction may limit the choice of birth location and the use of birthing pools.
- Some women/birthing parents report they found induced labour more painful.
- Outpatient induction offers less frequent monitoring and may increase the chance of undetected complications.
- Induction may not lead to active labour.

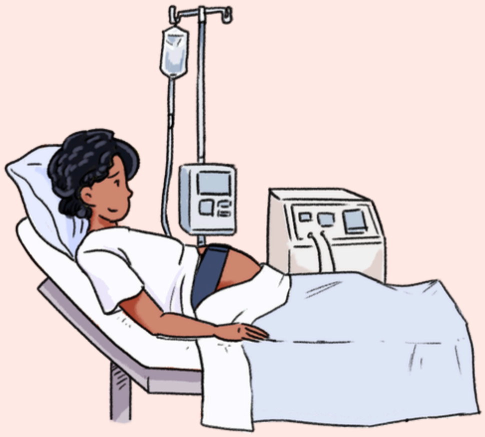

- You may need an instrumental vaginal birth, but this risk is no higher than if labour starts on its own (spontaneously).
- You may have heavy bleeding after birth (postpartum haemorrhage).
- Infection: your chance may be higher if your labour is longer than average, or your waters break early in the induction.
- Cervical ripening methods (membrane sweeping, medications, mechanical methods) can be painful when inserted/carried out.
- Increased chance of serious pelvic floor injury (perineal tear) if your baby is large for dates or you have an instrumental birth.

Risks in specific circumstances

- Previous caesarean birth: higher chance of uterine rupture and emergency caesarean birth if you are induced.

Chance of uterine rupture

2 in 1000 → **4 in 1000**

Spontaneous labour → Induction of labour

Chance of unplanned/emergency caesarean birth

25 in 100 → **35 in 100**

Spontaneous labour → Induction of labour

FUNDED BY NIHR | National Institute for Health and Care Research

University of Liverpool

NHS Liverpool Women's NHS Foundation Trust

Risks for baby:

Contractions becoming too frequent during induction is managed by:

- stopping hormone drip
- giving an injection to stop contractions
- an emergency caesarean

Delaying starting the oxytocin drip may mean that your labour takes longer and there may be an increased risk of your baby developing an infection. Induction can be delayed due to hospital staffing or demand.

Emergency situations:

Depending on your individual situation, there is a small risk of certain serious complications, which may lead to an emergency caesarean birth or other procedures:

- Cord prolapse – when we break your waters the baby's umbilical cord may fall into your vagina
- Shoulder dystocia – where the baby's shoulders get stuck during birth
- Abruption – Bleeding from behind the placenta
- Uterine rupture – particularly if you've had a previous caesarean birth or uterine surgery

You should **tell your midwife immediately** if you have:

- Significant pain
- Bleeding
- Reduced fetal movements

Alternatives

The decision to have an induction of labour is entirely up to you. You can choose not have an induction of labour. The alternatives are spontaneous labour and vaginal birth or a caesarean birth.

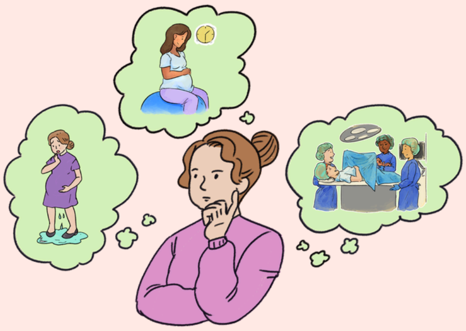

Intuition

How you feel and any gut feelings are important. You can speak to your midwife at any point during an induction if you feel uneasy about something.

Do nothing

You can do nothing and wait for labour to come on.

Monitoring you and your baby during induction of labour

Birth Options

Before your induction begins:

- Your midwife will assess the baby's position and may perform an ultrasound to check.
- You will be offered a vaginal exam to check the readiness of your cervix, which helps track the progress of induction.
- Your baby's heart rate will be monitored using a CTG (cardiotocography).

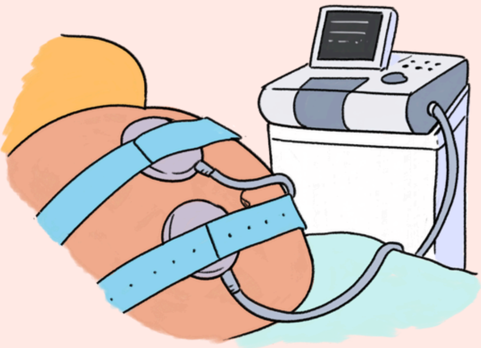

Wired cardiotocography

During your induction:

- Your baby's heartrate will be monitored before and after your cervical ripening medication or device.
- Your midwife will monitor contractions and the baby's heart rate as needed.
- Once labour is established, vaginal examinations are advised every 4 hours to track progress.
- If an oxytocin drip is used continuous heart rate monitoring is recommended to monitor your baby's heart pattern in response to your contractions.
- If the drip is not used your doctor or midwife may still recommend continuous heart rate monitoring depending on your situation.

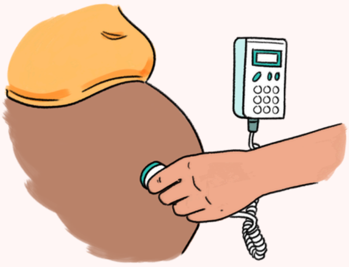

Doppler heart rate monitoring

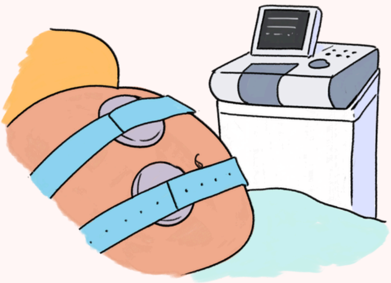

Wireless (telemetry) cardiotocography

During your labour:

- Your midwife will assess your baby's heart rate, sometimes a fetal scalp electrode is needed.
- Your temperature, heart rate, and blood pressure (vital signs) are checked regularly throughout your labour.

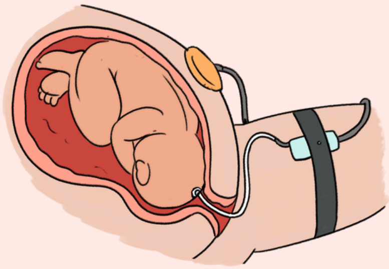

Fetal scalp electrode

FUNDED BY NIHR | National Institute for Health and Care Research
University of Liverpool
NHS Liverpool Women's NHS Foundation Trust

Pain relief during Induction

Birth Options

Pain Relief Option	Onset	Duration	Other Information
Tablets:<br>Paracetamol<br>Dihydrocodeine 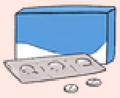	Around 1 hour	4-6 hours	Mild to moderate pain relief.
Gas & Air (Entonox) 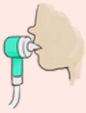	Immediate	Wears off within a few minutes	Provides mild pain relief, does not fully eliminate pain. Safe for both mother and baby, can be used at any stage of labour.
Injections (morphine, pethidine, diamorphine) 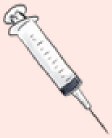	Around 30 minutes	Lasts a few hours	Strong painkillers that may cause drowsiness or slow breathing in mother and baby; anti-sickness medication may be needed due to feeling sick which is a common side effect.
Epidural 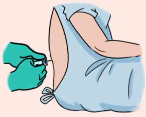	Around 40 minutes	Continuous relief throughout labour	Very effective, doesn't cause drowsiness or sickness, minimal effect on baby, but may increase the likelihood of instrumental birth (e.g. forceps or ventouse).
Patient-Controlled Intravenous Analgesia (PCA) 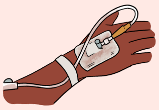	Quick via pump	Short-term, effective for contractions	Allows self-administered opioid relief, can be combined with gas & air, requires close monitoring for potential side effects like reduced breathing rate or oxygen levels.
Sterile Water Injections 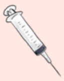	Relief starts around 10 minutes	Up to 3 hours	Useful for relieving back pain, may cause a brief stinging sensation at injection site.
Other Techniques 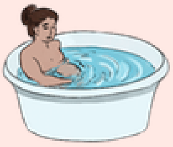	Immediate	Depends on how they work for you	Includes breathing exercises, TENS showers, baths/birthing pool, and massages; natural methods for early labour pain relief.

FUNDED BY NIHR National Institute for Health and Care Research
UNIVERSITY OF LIVERPOOL
NHS Liverpool Women's NHS Foundation Trust

Induction of labour:
Which type of birth are you likely to have?

Birth Options

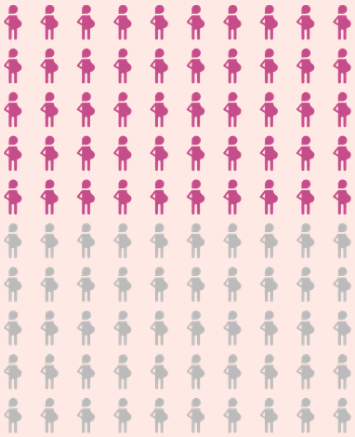

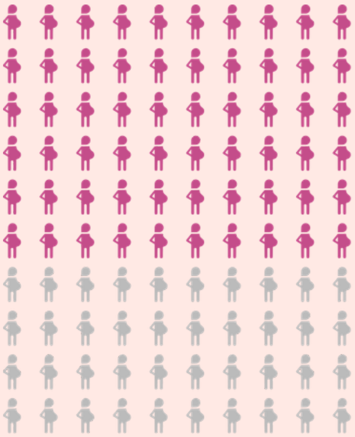

50–60

in 100

women who are induced have a vaginal birth without assistance

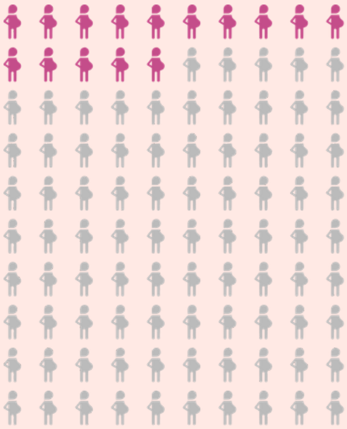

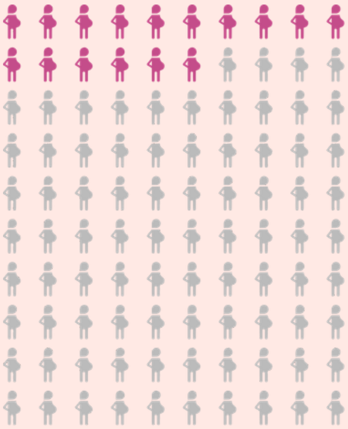

15–16

in 100

women who are induced have an instrumental birth

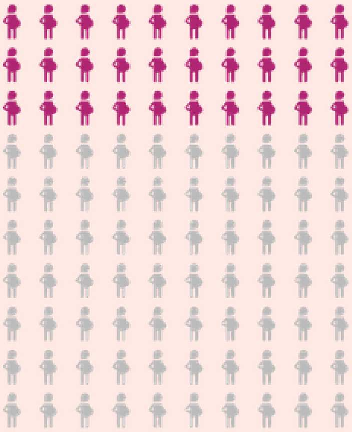

30 in 100 women who are induced have an unplanned/emergency caesarean section

These figures are for all women giving birth.

- As maternal age increases, the likelihood of having a caesarean after an induction of labour also tends to rise.
- Women from ethnic minority backgrounds who give birth at or beyond 41 weeks may also face a higher chance of caesarean birth.

FUNDED BY NIHR | National Institute for Health and Care Research

University of Liverpool

NHS Liverpool Women's NHS Foundation Trust

What you may need to know once you have chosen an induction of labour

Eating and drinking during the induction process:

- During your induction, you can eat and drink as you please unless advised otherwise.

Moving around during induction e.g. birthing balls, showers & baths:

- During the first part of your induction, you can move around, take showers and baths and use a birthing ball.
- Once in active labour, your movement may be more restricted by monitoring equipment, treatments required and pain relief choices.

What happens on the day:

- The practicalities of what will happen on the day of your induction will vary between hospitals. Discuss these with your team.

Key questions that you may wish to ask:

- Where do I go?
- Where will I stay?
- How long can my birth partner stay with me?
- Can my birth partner stay overnight?

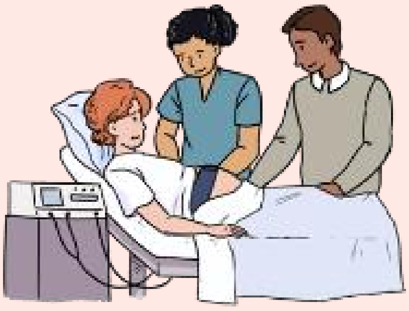

In most units you will start in one part of the labour ward and move to a different part when you are having your waters broken

What to expect during an induction of labour

The flow diagram on the next page shows the process of induction of labour.
This will be individual to you.

Factors affecting success of induction:

· The readiness of your cervix based on Bishop's score
· If you've laboured before
· Baby's growth
· Your health and age

Calculating a Bishops Score

- Dilatation – How open is the cervix
- Effacement – How thin is the cervix
- Station – Where is baby's head
- Position – Where is the cervix
- Consistency – How does the cervix feel

Partner involvement:

Partners or supporters are welcome during labour and can help provide emotional and practical support. Ask your local unit for their policy.

FUNDED BY NIHR | National Institute for Health and Care Research
UNIVERSITY OF LIVERPOOL
NHS Liverpool Women's NHS Foundation Trust

Start: you arrive at hospital

An initial assessment with your clinician.

- Discussion
- Observations
- Vaginal examination*
- Monitor baby's heart rate (CTG)
- +/- membrane sweep

Birth Options

*The readiness of your cervix will be checked by calculating your Bishop Score. Bishop Scores range from 0-13 with 13 being the most ready for labour.

If your cervix is ready (Bishop score of 7 or above)

If your cervix is not ready (Bishop score of 6 or below)

Step 1: You will be offered a vaginal medication (prostaglandin) or device in your cervix

You will have a **risk assessment** to decide where you will be while you wait. This can be up to 24 hours.

Inpatient induction (hospital)

Outpatient induction (home)

Labour may begin at any point

Step 2: Reassessment and re-examination to see if your cervix is open

Cervix is open

Cervix is not open

Options:

- Revisiting step 1- perhaps trying an alternative
- Rest
- Wait and see (may not be recommended)
- Caesarean birth

You will be transferred to a labour area

Your waters will be broken (amniotomy/artificial rupture of membranes).

Wait and see how labour progresses

You may be started on an **oxytocin drip** to encourage contractions (aim is 3-4 contractions in 10 minutes)

Aim: Progress through first stage of labour (4-10cm dilation, active phase) and into second stage (10cm dilated)

Birth of baby

FUNDED BY NIHR | National Institute for Health and Care Research

University of Liverpool

NHS Liverpool Women's NHS Foundation Trust

Postnatal Information: After induced labour

If you had a vaginal birth:

- Depending on what happened during your induction e.g. If you had an instrumental birth or an epidural, your hospital stay, and recovery may be longer.
- Your chances of breastfeeding when you leave hospital are like those who spontaneously labour.

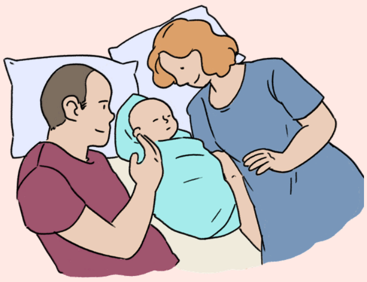

If you had a caesarean birth:

There is a Birth Options Caesarean Birth Postnatal core information set you may find helpful.

Satisfaction with birth experience:

- Experiences of induction vary, with some women and birthing people reporting positive experiences and others reporting disappointment or dissatisfaction.
- These feelings may be influenced by the reasons labour was induced, which often include concerns about the mother's or baby's health.
- Some women report disappointment at not having a spontaneous vaginal birth.

Your mental health:

Everyone's induction of labour experience is different. Talk to your midwife or GP if you experience any sudden changes in your mood after your birth.

Longer-term:

There is little or no evidence from maternal or childhood follow-up studies.

FUNDED BY NIHR | National Institute for Health and Care Research
UNIVERSITY OF LIVERPOOL
NHS Liverpool Women's NHS Foundation Trust

WHERE WE GOT THIS INFORMATION FROM

Birth Options

When and why an induction of labour may be offered

- National Institute for Health and Care Excellence (2021) Inducing labour. www.nice.org.uk/guidance/ng207 - accessed 23/01/2026
- https://digital.nhs.uk/data-and-information/publications/statistical/nhs-maternity-statistics/2024-25 - accessed 23/01/2026
- National Institute for Health and Care Excellence: Hypertension in pregnancy: diagnosis and management. [NG133] [Internet]. NICE, London; 2019 [cited 2024 Aug 14]. Available from: https://www.nice.org.uk/guidance/NG133
- National Institute for Health and Care Excellence: Diabetes in pregnancy: management from preconception to the postnatal period [NG3] [Internet]. NICE, London; 2015 [cited 2024 Aug 14]. Available from: https://www.nice.org.uk/guidance/ng3
- Girling J, Knight CL, Chappell L, the Royal College of Obstetricians and Gynaecologists. Intrahepatic cholestasis of pregnancy: Green-top Guideline No. 43 June 2022. BJOG Int J Obstet Gynaecol [Internet]. 2022 Dec [cited 2024 Aug 14];129(13). Available from: https://obgyn.onlinelibrary.wiley.com/doi/10.1111/1471-0528.17206
- Morris RK, Johnstone E, Lees C, Morton V, Smith G, the Royal College of Obstetricians and Gynaecologists. Investigation and Care of a Small-for-Gestational-Age Fetus and a Growth Restricted Fetus (Green-top Guideline No. 31). BJOG Int J Obstet Gynaecol [Internet]. 2024 Aug [cited 2024 Aug 14];131(9). Available from: https://obgyn.onlinelibrary.wiley.com/doi/10.1111/1471-0528.1781
- RCOG [Internet]. [cited 2024 Aug 14]. Reduced Fetal Movements (Green-top Guideline No. 57). Available from: https://www.rcog.org.uk/guidance/browse-all-guidance/green-top-guidelines/reduced-fetal-movements-green-top-guideline-no-57/
- National Institute for Health and Care Excellence: Twin and triplet pregnancy [NG137] [Internet]. NICE. London; 2019 [cited 2024 Aug 14]. Available from: https://www.nice.org.uk/guidance/NG137
- RCOG [Internet]. [cited 2024 Aug 14]. Induction of Labour at Term in Older Mothers (Scientific Impact Paper No. 34). Available from: https://www.rcog.org.uk/guidance/browse-all-guidance/scientific-impact-papers/induction-of-labour-at-term-in-older-mothers-scientific-impact-paper-no-34/

Options for starting labour and induction of labour

- National Institute for Health and Care Excellence (2021) Inducing labour. www.nice.org.uk/guidance/ng207 - accessed 23/01/2026
- Hofmeyr GJ, Gülmezoglu AM, Pileggi C. Vaginal misoprostol for cervical ripening and induction of labour. Cochrane Database of Systematic Reviews 2010, Issue 10. Art. No.: CD000941. DOI: 10.1002/14651858.CD000941.pub2
- Kerr RS, Kumar N, Williams MJ, Cuthbert A, Aflaifel N, Haas DM, Weeks AD. Low-dose oral misoprostol for induction of labour. Cochrane Database of Systematic Reviews 2021, Issue 6. Art. No.: CD014484. DOI: 10.1002/14651858.CD014484
- Rattanakanokchai S, Gallos ID, Kietpeerakool C, Eamudomkarn N, Alfirevic Z, Oladapo OT, et al. Methods of induction of labour: a network meta-analysis. Cochrane Pregnancy and Childbirth Group, editor. Cochrane Database Syst Rev [Internet]. 2023 Jan 3 [cited 2024 Aug 15];2023(1). Available from: http://doi.wiley.com/10.1002/14651858.CD015234
- De Vaan MD, Ten Eikelder ML, Jozwiak M, Palmer KR, Davies-Tuck M, Bloemenkamp KW, et al. Mechanical methods for induction of labour. Cochrane Pregnancy and Childbirth Group, editor. Cochrane Database Syst Rev [Internet]. 2023 Mar 30 [cited 2024 Aug 15];2023(7). Available from: http://doi.wiley.com/10.1002/14651858.CD001233.pub4
- Recommendations: Intrapartum care: Guidance 1.8.40 to 1.8.42. NICE. Available at: https://www.nice.org.uk/guidance/ng207/chapter/recommendations
- Smyth RMD, Markham C, Dowswell T. Amniotomy for shortening spontaneous labour. Cochrane Database of Systematic Reviews 2013, Issue 6. Art. No.: CD006167. DOI: 10.1002/14651858.CD006167.pub4
- Leathersich SJ, Vogel JP, Tran TS, Hofmeyr GJ. Acute tocolysis for uterine tachysystole or suspected fetal distress. Cochrane Database of Systematic Reviews 2018, Issue 7. Art. No.: CD009770. DOI: 10.1002/14651858.CD009770.pub2

Alternative therapies and methods of starting labour

- National Institute for Health and Care Excellence (2021) Inducing labour. www.nice.org.uk/guidance/ng207 - accessed 23/01/2026

Supporting you to make an informed decision about induction of labour

- Wickham, S. (2002) What's Right For Me? Making decisions in pregnancy and childbirth. 2022 Edn. IBSN-978-1914465048

Birth Options

Benefits of induction of labour

- RCOG. Induction of Labour at Term in Older Mothers 2013. Available from: https://www.rcog.org.uk/media/lp4n13jn/sip_34.pdf.
- RCOG. Care of women with obesity in pregnancy. 2018. Available from: https://www.rcog.org.uk/guidance/browse-all-guidance/green-top-guidelines/care-of-women-with-obesity-in-pregnancy-green-top-guideline-no-72/
- National Institute for Health and Care Excellence (2021) Inducing labour. www.nice.org.uk/guidance/ng207 - accessed 23/01/2026
- Middleton P, Shepherd E, Crowther CA. Induction of labour for improving birth outcomes for women at or beyond term. Cochrane Database of Systematic Reviews. 2018(5).
- MBRRACE-UK IGa. State of the nation report | MBRRACE-UK. 2025.
- Boulvain M, Thornton JG. Induction of labour at or near term for suspected fetal macrosomia. Cochrane Database of Systematic Reviews. 2023(3).
- Bugg GJ, Siddiqui F, Thornton JG. Oxytocin versus no treatment or delayed treatment for slow progress in the first stage of spontaneous labour. Cochrane Database Syst Rev. 2013(6):Cd007123.
- Rcog. Birth After Previous Caesarean Birth 2015 [Available from: https://www.rcog.org.uk/media/kpkjwd5h/gtg_45.pdf.
- Li J, Shao X, Song S, Liang Q, Liu Y, Qi X. Immediate versus delayed induction of labour in hypertensive disorders of pregnancy: a systematic review and meta-analysis. BMC Pregnancy and Childbirth. 2020;20(1):735.
- Krogh LQ, Glavind J, Henriksen TB, Thornton J, Fuglsang J, Boie S. Full-term induction of labor vs expectant management and cesarean delivery in women with obesity: systematic review and meta-analysis. American Journal of Obstetrics & Gynecology MFM. 2023;5(5):100909.
- Denison F, Aedla N, Keag O, Hor K, Reynolds R, Milne A, et al. Care of Women with Obesity in Pregnancy. BJOG: An International Journal of Obstetrics & Gynaecology. 2019;126(3):e62-e106.

Risks of induction of labour

- National Institute for Health and Care Excellence (2021) Inducing labour. www.nice.org.uk/guidance/ng207 - accessed 23/01/2026
- Middleton P, Shepherd E, Morris J, Crowther CA, Gomersall JC. Induction of labour at or beyond 37 weeks' gestation. Cochrane Database Syst Rev. 2020;7(7):Cd004945.
- Harkness M, Yuill C, Cheyne H, McCourt C, Black M, Pasupathy D, et al. Experience of induction of labour: a cross-sectional postnatal survey of women at UK maternity units. BMJ Open. 2023;13(5):e071703.
- RCOG. Birth After Previous Caesarean Birth 2015 [Available from: https://www.rcog.org.uk/media/kpkjwd5h/gtg_45.pdf.
- Caesarean birth. London: National Institute for Health and Care Excellence (NICE); 2024 Jan 30. PMID: 33877751. Available from: https://www.nice.org.uk/guidance/ng192/chapter/Recommendations.
- National Institute for Health and Care Excellence: Guidelines. Diabetes in pregnancy: management from preconception to the postnatal period. London: National Institute for Health and Care Excellence (NICE)Copyright © NICE 2020.; 2020.
- Li J, Shao X, Song S, Liang Q, Liu Y, Qi X. Immediate versus delayed induction of labour in hypertensive disorders of pregnancy: a systematic review and meta-analysis. BMC Pregnancy and Childbirth. 2020;20(1):735.
- Morris RK, Johnstone E, Lees C, Morton V, Smith G, Obstetricians tRCo, et al. Investigation and Care of a Small-for-Gestational-Age Fetus and a Growth Restricted Fetus (Green-top Guideline No. 31). BJOG: An International Journal of Obstetrics & Gynaecology. 2024;131(9):e31-e80.
- WHO Guidelines Approved by the Guidelines Review Committee. WHO recommendations on induction of labour, at or beyond term. Geneva: World Health Organization © World Health Organization 2022.; 2022.
- Denison F, Aedla N, Keag O, Hor K, Reynolds R, Milne A, et al. Care of Women with Obesity in Pregnancy. BJOG: An International Journal of Obstetrics & Gynaecology. 2019;126(3):e62-e106.
- RCOG. Umbilical Cord Prolapse 2014 [updated 2024. Available from: https://www.rcog.org.uk/media/3wykswng/gtg-50-umbilicalcordprolapse-2014.pdf.
- RCOG. Shoulder Dystocia 2012 [Third:[Available from: https://www.rcog.org.uk/media/ewgpnmio/gtg_42.pdf.

Monitoring you and your baby during the induction of labour

- National Institute for Health and Care Excellence (2021). Overview/ Inducing Labour/ Guidance/ NICE. (online) www.nice.org.uk
- National Institute for Health and Care Excellence (2022). Overview/ Fetal monitoring in labour/ Guidance/ NICE. (online) www.nice.oeg.uk
- Trust, N. National C. (2022) Induced labour: reasons, pros and cons/ Pregnancy, Your pregnancy week by week articles and support/ NCT (National Childbirth Trust)
- National Institute for Health and Care Excellence (2023). Overview/ Intrapartum Care/ Guidance/ NICE. (online) www.nice.org.uk
- www.nice.org.uk. (N.D) Information for the public/ Inducing labour/ Guidance/ NICE. (online)
- NHS National Institute for Health Research (2016) Choices when pregnancy reached 41 weeks.
- Gülmezoglu AM (2012) Induction of labour for improving birth outcomes for women at or beyond term. Cochrane Database Systematic Review. 2012 Jun 13 ;(6): CD004945.
- Stock S (2012) Outcomes of elective induction of labour compared with expectant management: population based study. British Medical Journal. BMJ 2012;344:e2838 doi: 10.1136/bmj.e2838 (Published 10 May 2012)
- Middleton P. et al. (2020) Induction of labour in women with normal pregnancies at or beyond 37 weeks. (online) www.cochrane.org.

Pain relief during induction of labour

- National Institute for Health and Care Excellence (2021) Inducing labour. www.nice.org.uk/guidance/ng207 - accessed 23/01/2026
- National Institute for Health and Care Excellence (2023) Intrapartum Care. https://www.nice.org.uk/guidance/ng235/resources/intrapartum-care-pdf-66143897812933 - accessed 23/01/2026
- Othman M, Jones L, Neilson JP. Non-opioid drugs for pain management in labour. Cochrane Database of Systematic Reviews 2012, Issue 7. Art. No.: CD009223. DOI: 10.1002/14651858.CD009223.pub2. Accessed 17 March 2025.
- Middleton P, Shepherd E, Morris J, Crowther CA, Gomersall JC. Induction of labour at or beyond 37 weeks' gestation. Cochrane Database of Systematic Reviews 2020, Issue 7. Art. No.: CD004945. DOI: 10.1002/14651858.CD004945.pub5. Accessed 18 March 2025.

FUNDED BY NIHR | National Institute for Health and Care Research

UNIVERSITY OF LIVERPOOL

NHS Liverpool Women's NHS Foundation Trust

WHERE WE GOT THIS INFORMATION FROM

Birth Options

Induction of labour: which type of birth are you likely to have?

- National Institute for Health and Care Excellence (2021) Inducing labour. www.nice.org.uk/guidance/ng207 - accessed 23/01/2026
- National Maternity & Perinatal Audit: Induction of labour Snapshot. 2025
- NHS Maternity Statistics, England, 2023-24 - NHS England Digital – accessed 14/03/2025

What to expect during an induction of labour

- Recommendations: Inducing labour: Guidance. NICE. Available at: https://www.nice.org.uk/guidance/ng207/chapter/recommendations
- Marconi AM, Bozzetti P, Morabito A, et al.: Comparing two dinoprostone agents for cervical ripening and induction of labor: A randomized trial. Eur J Obstet Gynecol Reprod Biol. 2008;138(2):135–40. 10.1016/j.ejogrb.2007.08.009
- Batinelli L, Serafini A, Nante N, Petraglia F, Severi FM, Messina G. Induction of labour: clinical predictive factors for success and failure. J Obstet Gynaecol. 2018 Apr;38(3):352-358. doi: 10.1080/01443615.2017.1361388. Epub 2017 Oct 23. PMID: 29058493.
- The indication for induction of labor impacts the risk of cesarean delivery. J Matern Fetal Neonatal Med. 2014;29(2):224–8. 10.3109/14767058.2014.993965
- Ellis JA, Brown CM, Barger B, et al.: Influence of Maternal Obesity on Labor Induction: A Systematic Review and Meta-Analysis. J Midwifery Women's Health. 2019;64(1):55–67. 10.1111/jmwh.12935
- Recommendations | Intrapartum care | Guidance | NICE – accessed 14/03/2025
- Recommendations: Antenatal care: Guidance: available at: https://www.nice.org.uk/guidance/ng201#:~:text=This%20guideline%20includes%20recommendations%20on:%20organisation#:~:text=This%20guideline%20includes%20recommendations%20on:%20organisation
- Bohren MA, Hofmeyr GJ, Sakala C, Fukuzawa RK, Cuthbert A. Continuous support for women during childbirth. Cochrane Database of Systematic Reviews 2017, Issue 7. Art. No.: CD003766. DOI: 10.1002/14651858.CD003766.pub6.
- Bohren MA, Berger BO, Munthe-Kaas H, Tunçalp Ö. Perceptions and experiences of labour companionship: a qualitative evidence synthesis. Cochrane Database of Systematic Reviews 2019, Issue 3. Art. No.: CD012449. DOI: 10.1002/14651858.CD012449.pub2.
- Jakub M, Marta M, Jagoda G, Kamila G, Stanislaw G. Is Unfavourable Cervix prior to Labor Induction Risk for Adverse Obstetrical Outcome in Time of Universal Ripening Agents Usage? Single Center Retrospective Observational Study. J Pregnancy. 2020 Sep 1;2020:4985693. doi: 10.1155/2020/4985693. PMID: 32953176; PMCID: PMC7481947.

What you may need to know once you have chosen an induction of labour

- Singata, Mandisa, et al. 'Restricting Oral Fluid and Food Intake during Labour'. Cochrane Database of Systematic Reviews, no. 8, 2013. www.cochranelibrary.com, https://doi.org/10.1002/14651858.CD003930.pub3.

Postnatal Information: After induced labour

- RCOG Third- and Fourth-degree Perineal Tears, Management 2015 [Green-top Guideline No. 29]. Available from: https://www.rcog.org.uk/media/5jeb5hzu/gtg-29.pdf.
- Braund S, Deneux-Tharaux C, Sentilhes L, Seco A, Rozenberg P, Goffinet F. Induction of labor and risk of postpartum hemorrhage in women with vaginal delivery: A propensity score analysis. International Journal of Gynecology & Obstetrics. 2024;164(2):732-40.
- Khireddine I, Le Ray C, Dupont C, Rudigoz RC, Bouvier-Colle MH, Deneux-Tharaux C. Induction of labor and risk of postpartum hemorrhage in low risk parturients. PLoS One. 2013;8(1):e54858.
- Coates R. Attitudes of pregnant women and healthcare professionals to labour induction and obtaining consent for labour induction. Best Pract Res Clin Obstet Gynaecol. 2021 Nov;77:64-75. doi: 10.1016/j.bpobgyn.2021.08.008.

How we present information about risk

Very common	1 in 1 to 1 in 10
Common	Less than 1 in 10 to 1 in 100
Uncommon	Less than 1 in 100 to 1 in 1000
Rare	Less than 1 in 1000 to 1 in 10,000
Very rare	Less than 1 in 10,000

FUNDED BY NIHR | National Institute for Health and Care Research

UNIVERSITY OF LIVERPOOL

NHS Liverpool Women's NHS Foundation Trust
