# Supplementary figures and images for: What should be discussed when considering an induction of labour? A UK-wide, multi-centre Delphi study to develop a core information set for induction of labour
Source: BMJ Open. 2026 May 27;16(5):e118024. doi: 10.1136/bmjopen-2026-118024 (PMC13218194; doi:10.1136/bmjopen-2026-118024)

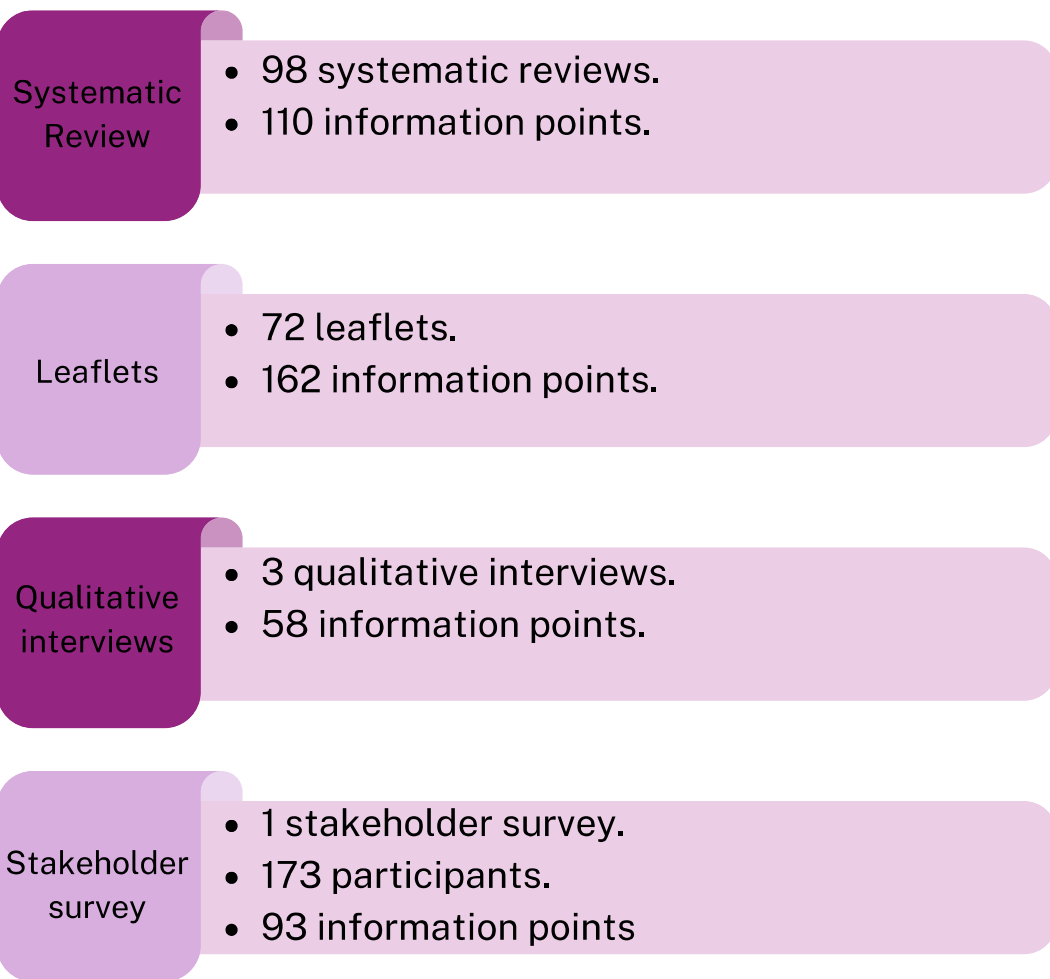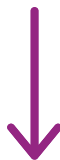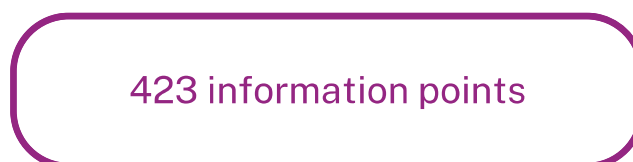

*Amalgamated and duplicates removed...*

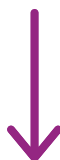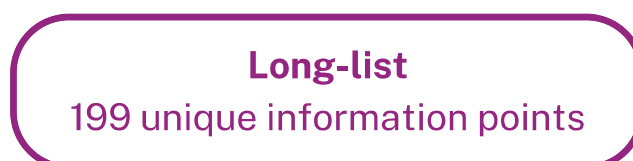

Supplement: online supplemental file 9 [file bmjopen-16-5-s009.pdf]
