# Supplementary material for: What should be discussed when considering an induction of labour? A UK-wide, multi-centre Delphi study to develop a core information set for induction of labour
Source: BMJ Open. 2026 May 27;16(5):e118024. doi: 10.1136/bmjopen-2026-118024 (PMC13218194; doi:10.1136/bmjopen-2026-118024)
Supplement: online supplemental file 10 [file bmjopen-16-5-s010.pdf]

**Induction**	1,2,3	7,8,9		
Questions	NOT critical	Critical	Consensus	In or Out R2
	%	%		
How common induction of labour is	10.6	38.9	No	In
Reasons that induction of labour could be planned e.g. Diabetes, aged 40+, IVF pregnancy, multiple pregnancies, chronic conditions, personal request.	1.6	71	No	In
When induction can be offered for example post-dates, at certain times for some conditions (diabetes, twins)	0.8	73.7	No	In
Unexpected reasons induction of labour could be offerede.g. pre-labour rupture of membranes, concerns with mum's health, obstetric cholestasis, infection, bleeding, pre-eclampsia, severe maternal illness, small/large baby, growth problems for baby, post-term	5.2	73.1	No	In
The role of membrane sweeping to ripen the cervix	1.3	78.5	No	In
Methods to begin induction of labour by ripening the cervix, including medical (propess, prostin, misoprostol) and mechanical methods (balloon catheter, rods)	0.6	88.1	Yes	In
Methods of inducing contractions including artificial rupture of membranes and the hormone drip	0.8	87.9	Yes	In
The evidence to support/dispute complementary therapies that birthing people may try e.g. acupuncture, homeopathy, nipple stimulation, castor oil, massaging breasts, raspberry leaf tea, curry, fresh pineapple, eating 6 dates a day, reflexology, aromatherapy, sexual intercourse, keep active-walking, primrose oil, vaginal douching prior to induction	8.2	56.9	No	In
The possible locations of inductionse.g. at home or in hospital	0.5	79.8	No	In
Care during induction of labour e.g. during ripening phase and labour, how often will be checked (vaginal examinations)	0	84.3	Yes	In
Induction urgency levels and its impact on the length of the process	0	87.5	Yes	In
Factors affecting whether induction leads to active labour e.g. how far a long pregnancy is, how 'ripe' cervix is at the start of induction	0	84.6	Yes	In
Potential variation in length of induction of labour	0	79.8	No	In
Ability to speed up/slow down process of contractions, and the pro's and con's of doing this	0.6	81.6	Yes	In

Birth of the placenta and reducing bleeding after birth (management of the third stage)	1.6	76.8	No	In
Information regarding partners, their presence and role during the birth.	2.1	72.2	No	In
Considerations with induction for the mothere.g. may not work, may take several days work, may involve more vaginal exams, place of birth may need to change from original plan, may not be able to use pool	0	94.9	Yes	In
Possible risks for the mother with induction of laboure.g. increased risk of bleeding after birth, infection in the lining of the womb, need for antibiotics	0	95.4	Yes	In
Possible risks with induction of labour for the babye.g. fetal distress due to no rest between contractions, changes in babies heart rat	0	97.9	Yes	In
Benefits of induction for mother e.g. reduce risk of caesarean birth, give birth earlier, planned date for start of induction, less anxiet	2.1	83.2	Yes	In
Benefits of induction for the baby e.g. reduce risks of stillbirth, reduce risks of infection (in some situations)	1.5	88.9	Yes	In
The different risks associated with cervical ripening. Including the side effects of the specific medical and mechanical methods used e.g. nausea, diarrhoea, vaginal soreness	1.1	83	Yes	In
The different risks associated with induction of contractionse.g. uterus contracting too frequently	0.3	88.6	Yes	In
Red flags to alert midwifery/medical staff to during the induction e.g. pain, bleeding, reduced fetal movements	0.3	95.4	Yes	In
Specifically important risk situationse.g. Previous caesarean section, small baby, BMI.	0.3	83.2	Yes	In
How they will be monitored during induction e.g. vital signs (temperature, pulse, respiration, blood pressure), differences between cervical ripening and induction of contractions	1	72.9	No	In
How their baby will be monitored during induction e.g. baby heart trace, differences between cervical ripening and induction of contractions	0.3	80.4	Yes	In
Possible emergency situationse.g. Cord prolapse, caesarean section, shoulders getting stuck at birth	0.3	84.1	Yes	In
What happens if active labour does not commence e.g. resting, starting again, a different method or caesarean birth	0.6	83.8	Yes	In
We may not know why active labour does not commence for you	6.1	56.4	No	In
The general facts about how many women/birthing people having an induction of labour have spontaneous vaginal birth, assisted vaginal birth and emergency caesarean birth	8.2	61.7	No	In

Postnatal issues following induction e.g. mental and physical health	0.9	75	No	In
Different pharmacological pain relief options availablee.g. Gas & air (Entonox), Oral (paracetamol/dihydrocodeine), Injected (morphine/pethidine), Remifentanyl patient controlled analgesia, Epidural)	1.1	82.4	Yes	In
Different non-pharmacological pain relief options available e.g. TENS, water, water injections	1.8	78.1	No	In
Impact of induction of labour on pain experienced	1	83.8	Yes	In
Eating and drinking during the induction process	2.2	68.6	No	In
Moving around during inductione.g. birthing balls, showers & baths	2.2	75.3	No	In
What happens on the day	1.1	83.7	Yes	In
The financial costs to the NHS for induction of labour	71.8	6.7	No	In
What could happen if you do nothing and decide to wait for labour to come naturally e.g. stillbirth, not going into labour naturally despite waiting	1.1	87.8	Yes	In
Decision making for induction e.g. choice lies with the woman/birthing person, offering of alternative options, ability to say no(	0.5	93.4	Yes	In
Maternal satisfaction with induction of labour	2.7	66.6	No	In
Information on the concerns people might have e.g. fear of delay, being alone, having a feeling of time being up	3.2	64.1	No	In
Impact on future pregnancies	2.4	76.6	No	In
Comparison of planned caesarean section versus induction of labour	2.9	76.5	No	In
Having a structured way of approaching decisionse.g. the 'BRAIN' tool (benefits, risks, alternatives, intuition, do nothing)	5.1	69.4	No	In

Remove from second round is >= 80% of one group think it is of no importance, and <15% think it is critical

Remove if:

>=80% think 1,2,3

[illegible]
