# Supplementary material for: What should be discussed when considering an induction of labour? A UK-wide, multi-centre Delphi study to develop a core information set for induction of labour
Source: BMJ Open. 2026 May 27;16(5):e118024. doi: 10.1136/bmjopen-2026-118024 (PMC13218194; doi:10.1136/bmjopen-2026-118024)
Supplement: online supplemental file 11 [file bmjopen-16-5-s011.pdf]

KEY:
Consensus IF ≥80% score 7,8,9 (critically important) AND ≤15% score 1,2,3 (Limited importance).
No consensus IF <80% score 7,8,9 (critically important) OR >15% score 1,2,3 (Limited importance).
Consensus out IF ≥80% score 1,2,3 (Limited importance) AND ≤15% score 7,8,9 (Critcally important).

In if either parents or professionals, **consensus**.
Discuss if both parents and professionals, **no consensus.**
Out if either parents or professionals, **consensus out.**

0	0	Parents	Professionals	In/Out/Discuss
Section	Question	Outcome	Outcome	
r2_section1_q1	How common induction of labour is	NO CONSENSUS	NO CONSENSUS	Discuss
r2_section1_q2	Reasons that induction of labour could be planned e.g. Diabetes, aged 40+, IVF pregnancy, multiple pregnancies, chronic conditions, personal request.	CONSENSUS	CONSENSUS	In
r2_section1_q3	When induction can be offered for example post-dates, at certain times for some conditions (diabetes, twins)	CONSENSUS	CONSENSUS	In
r2_section1_q4	Unexpected reasons induction of labour could be offerede.g. pre-labour rupture of membranes, concerns with mum's health, obstetric cholestasis, infection, bleeding, pre-eclampsia, severe maternal illness, small/large baby, growth problems for baby, post-term	CONSENSUS	CONSENSUS	In
r2_section2_q1	The role of membrane sweeping to ripen the cervix	CONSENSUS	CONSENSUS	In
r2_section2_q2	Methods to begin induction of labour by ripening the cervix, including medical (propess, prostin, misoprostol) and mechanical methods (balloon catheter, rods)	CONSENSUS	CONSENSUS	In
r2_section2_q3	Methods of inducing contractions including artificial rupture of membranes and the hormone drip	CONSENSUS	CONSENSUS	In
r2_section2_q4	The evidence to support/dispute complementary therapies that birthing people may try e.g. acupuncture, homeopathy, nipple stimulation, castor oil, massaging breasts, raspberry leaf tea, curry, fresh pineapple, eating 6 dates a day, reflexology, aromatherapy, sexual intercourse, keep active-walking, primrose oil, vaginal douching prior to induction	NO CONSENSUS	NO CONSENSUS	Discuss
r2_section3_q1	The possible locations of inductionse.g. at home or in hospital	CONSENSUS	CONSENSUS	In
r2_section3_q2	Care during induction of labour e.g. during ripening phase and labour, how often will be checked (vaginal examinations)	CONSENSUS	CONSENSUS	In
r2_section3_q3	Induction urgency levels and its impact on the length of the process	CONSENSUS	CONSENSUS	In
r2_section3_q4	Factors affecting whether induction leads to active labour e.g. how far a long pregnancy is, how 'ripe' cervix is at the start of induction	CONSENSUS	CONSENSUS	In
re_section3_q5	Potential variation in length of induction of labour	CONSENSUS	CONSENSUS	In
re_section3_q6	Ability to speed up/slow down process of contractions, and the pro's and con's of doing this	CONSENSUS	CONSENSUS	In
r2_section3_q7	Birth of the placenta and reducing bleeding after birth (management of the third stage)	CONSENSUS	CONSENSUS	In
re_section3_q8	Information regarding partners, their presence and role during the birth.	CONSENSUS	CONSENSUS	In
r2_section4_q1	Considerations with induction for the mothere.g. may not work, may take several days work, may involve more vaginal exams, place of birth may need to change from original plan, may not be able to use pool	CONSENSUS	CONSENSUS	In

r2_section4_q2	Possible risks for the mother with induction of laboure.g. increased risk of bleeding after birth, infection in the lining of the womb, need for antibiotics	CONSENSUS	CONSENSUS	In
r2_section4_q3	Possible risks with induction of labour for the babye.g. fetal distress due to no rest between contractions, changes in babies heart rate	CONSENSUS	CONSENSUS	In
r2_section4_q4	Benefits of induction for mother e.g. reduce risk of caesarean birth, give birth earlier, planned date for start of induction, less anxiety	CONSENSUS	CONSENSUS	In
r2_section4_q5	Benefits of induction for the baby e.g. reduce risks of stillbirth, reduce risks of infection (in some situations)	CONSENSUS	CONSENSUS	In
r2_section4_q6	The different risks associated with cervical ripening. Including the side effects of the specific medical and mechanical methods used e.g. nausea, diarrhoea, vaginal soreness	CONSENSUS	CONSENSUS	In
r2_section4_q7	The different risks associated with induction of contractionse.g. uterus contracting too frequently	CONSENSUS	CONSENSUS	In
r2_section4_q8	Red flags to alert midwifery/medical staff to during the induction e.g. pain, bleeding, reduced fetal movements	CONSENSUS	CONSENSUS	In
r2_section4_q9	Specifically important risk situationse.g. Previous caesarean section, small baby, BMI.	CONSENSUS	CONSENSUS	In
r2_section5_q1	How they will be monitored during induction e.g. vital signs (temperature, pulse, respiration, blood pressure), differences between cervical ripening and induction of contractions	CONSENSUS	CONSENSUS	In
r2_section5_q2	How their baby will be monitored during induction e.g. baby heart trace, differences between cervical ripening and induction of contractions	CONSENSUS	CONSENSUS	In
r2_section6_q1	Possible emergency situationse.g. Cord prolapse, caesarean section, shoulders getting stuck at birth.	CONSENSUS	CONSENSUS	In
r2_section6_q2	What happens if active labour does not commence e.g. resting, starting again, a different method or caesarean birth	CONSENSUS	CONSENSUS	In
r2_section6_q3	We may not know why active labour does not commence for you	NO CONSENSUS	NO CONSENSUS	Discuss
r2_section6_q4	The general facts about how many women/birthing people having an induction of labour have spontaneous vaginal birth, assisted vaginal birth and emergency caesarean birth	NO CONSENSUS	CONSENSUS	In
r2_section6_q5	Postnatal issues following induction e.g. mental and physical health	CONSENSUS	CONSENSUS	In
r2_section7_q1	Different pharmacological pain relief options availablee.g. Gas & air (Entonox), Oral (paracetamol/dihydrocodeine), Injected (morphine/pethidine), Remifentanyl patient controlled analgesia, Epidural)	CONSENSUS	CONSENSUS	In
r2_section7_q2	Different non-pharmacological pain relief options available e.g. TENS, water, water injections	CONSENSUS	CONSENSUS	In
r2_section7_q3	Impact of induction of labour on pain experienced	CONSENSUS	CONSENSUS	In
r2_section8_q1	Eating and drinking during the induction process	NO CONSENSUS	CONSENSUS	In
r2_section8_q2	Moving around during inductione.g. birthing balls, showers & baths	CONSENSUS	CONSENSUS	In
r2_section8_q3	What happens on the day	CONSENSUS	CONSENSUS	In
r2_section9_q1	The financial costs to the NHS for induction of labour	CONSENSUS OUT	CONSENSUS OUT	Out
r2_section10_q1	What could happen if you do nothing and decide to wait for labour to come naturally e.g. stillbirth, not going into labour naturally despite waiting	CONSENSUS	CONSENSUS	In
r2_section10_q2	Decision making for induction e.g. choice lies with the woman/birthing person, offering of alternative options, ability to say no	CONSENSUS	CONSENSUS	In

r2_section10_q3	Maternal satisfaction with induction of labour	NO CONSENSUS	CONSENSUS	In
r2_section10_q4	Information on the concerns people might have e.g. fear of delay, being alone, having a feeling of time being up	NO CONSENSUS	CONSENSUS	In
r2_section10_q5	Impact on future pregnancies	CONSENSUS	CONSENSUS	In
r2_section10_q6	Comparison of planned caesarean section versus induction of labour	CONSENSUS	CONSENSUS	In
r2_secton10_q7	Having a structured way of approaching decisionse.g. the 'BRAIN' tool (benefits, risks, alternatives, intuition, do nothing)	CONSENSUS	CONSENSUS	In
r2_section11_q1	Possible delays during the process of induction	CONSENSUS	CONSENSUS	In
