# Supplementary material for: What should be discussed when considering an induction of labour? A UK-wide, multi-centre Delphi study to develop a core information set for induction of labour
Source: BMJ Open. 2026 May 27;16(5):e118024. doi: 10.1136/bmjopen-2026-118024 (PMC13218194; doi:10.1136/bmjopen-2026-118024)
Supplement: Supplementary file 12 [file bmjopen-2026-118024.reviewer_comments.pdf]

PEER REVIEW HISTORY

BMJ Open publishes all reviews undertaken for accepted manuscripts. Reviewers are asked to complete a checklist review form and are provided with free text boxes to elaborate on their assessment. These free text comments are reproduced below.

ARTICLE DETAILS

Title (Provisional)

What should be discussed when considering an induction of labour? A UK-wide, multi-centre Delphi study to develop a core information set for induction of labour

Authors

Bunni, Eve Minna Leyshon; Kingdon, Carol; Bradley, Vicky; Hunt, Alexandra; Mahdi, Amy; Axcell, Thomas; Jagadish, Ria; Fox, Sophie; O'dair, Millie; Simms, Charlotte; Munn, Yee Tan; Bonnett, Laura; Greenfield, Benjamin; Cunningham, Caroline; Holt, Siobhan; Burden, Christy; Ficquet, Joanna; Otero-Romero, Elena; parry-smith, william; Black, Mairead; Merriel, Abi; Options Collaborative Group, Options Collaborative Group

---

VERSION 1 - REVIEW

Reviewer 1

Name East, Christine

Affiliation La Trobe University, School of Nursing and Midwifery / Judith Lumley Centre

Date 19-Mar-2026

COI None

Thank you for the invitation to review the manuscript, "What should be discussed when considering an induction of labour? A Delphi study to develop a core information set for induction of labour"

I have no doubt that the group has developed an "… induction of labour CIS [that] is intended to be immediately useful to clinicians tasked with delivering current clinical guideline recommendations for informed decision-making" – this guide will be a wonderful addition to have. A key strength of the research is that the patient and public involvement was embedded throughout. And how disappointing that, despite your efforts, there were no non-English speaking participants.

Addressing a few very minor points may improve the overall quality of the manuscript

1. P8, Line 20: The sentence "Round 2 of the survey was distributed via the emails left during round 1" needs clarity. I assume that participants were invited to 'leave' their email address

in the 1st round REDCap survey if the wished to be involved in round 2, but that could be clearer.

2. Were any mechanisms in place to protect from hoax respondents to the surveys, particularly noting the chance to win a monetary voucher? See, for example, https://doi.org/10.2196/68092. Perhaps a comment can be added to the Strengths and Limitations section.

3. When were the various elements of the work undertaken?

4. P10, line 52: please replace “1” with “One”

Congratulations on both undertaking and reporting this important and helpful exercise.

Reviewer **2**

Name **Mlodawski, J**

Affiliation **Jan Kochanowski University in Kielce, Obstetrics and Gynecology**

Date **23-Mar-2026**

COI **None**

The manuscript addresses an important, timely, and practically relevant topic. Developing a core information set for labor induction is valuable, because it responds to a real problem of insufficient and inconsistent information provided to patients considering induction.

However, I would suggest major revision, mainly because of methodological issues and several inconsistencies.

In round 1, as many as 87% of participants in the parent group were White British, and the dominant participants were people aged 31–40 with bachelor/postgraduate education. This means that the final CIS may reflect, to a greater extent, the informational needs of women who are more educationally and socially privileged, and more digitally active, rather than the pregnant population as a whole.

This is not really the ethnic diversity declared by the authors.

The authors report that 58 professionals participated in round 1, but only 23 in round 2. This is a substantial drop, which may affect the stability of the consensus between groups. Simply stating that this level of attrition “is not unusual for a Delphi” does not fully solve the problem.

One group included: people planning pregnancy, currently pregnant women, postpartum women, partners, and in addition professionals could also respond from the parent perspective. This raises the question whether “parent/non-professional” is really a homogenous analytical category?

The discussion and conclusions sometimes go one step too far, suggesting that standardization of the conversation by itself will reduce variation and improve access to information. This is probable, but it has not been demonstrated yet.

The authors could consider supplementing the section on factors influencing induction success and the course of induction in women with an unfavourable cervix with additional observational data (doi:10.1155/2020/4985693). This study may be particularly useful in the context of information given to patients about the probability of failed induction and the importance of baseline cervical status.

Reference numbers 22 and 26 appear twice in the reference list.

The authors state that the final CIS content was populated according to a predefined hierarchy of sources, with priority given to NICE, RCOG, and systematic reviews. This is reasonable, but it has consequences: not all issues considered important by participants necessarily have equally strong evidene base. The text should more clearly distinguish:

(1) what was considered important by stakeholders,

(2) what is well supported by high-quality evidence,

(3) where evidence gaps still exist.

I think that addressing the points above is able to improve the manuscript considerably. I am therefore looking forward to the revised version.

VERSION 1 - AUTHOR RESPONSE

Reviewer	Reviewer’s comment	Author's response
Dr. Christine East, La Trobe University	I have no doubt that the group has developed an “… induction of labour CIS [that] is intended to be immediately useful to clinicians tasked with delivering current clinical guideline recommendations for informed decision-making” – this guide will be a wonderful addition to have. A key strength of the research is that the patient and public involvement was embedded throughout. And how disappointing that, despite your efforts, there were no non-English speaking participants.<br><br>Addressing a few very minor points may improve the overall quality of	We thank the reviewer for their comprehensive comments. We have responded to Reviewer 1’s specific remarks on a point-by-point basis and made the suggested changes as detailed below.

	the manuscript.	
Dr. J Mlodawski, Jan Kochanowski University in Kielce	The manuscript addresses an important, timely, and practically relevant topic. Developing a core information set for labour induction is valuable, because it responds to a real problem of insufficient and inconsistent information provided to patients considering induction.<br><br>However, I would suggest major revision, mainly because of methodological issues and several inconsistencies.	We thank the reviewer for their comprehensive comments. We have responded to Reviewer 2's specific remarks on a point-by-point basis and made the suggested changes as detailed below.
**Item**	**Reviewer 1 comment**	**Author's response**
1.1	P8, Line 20: The sentence "Round 2 of the survey was distributed via the emails left during round 1" needs clarity. I assume that participants were invited to 'leave' their email address in the 1st round REDCap survey if the wished to be involved in round 2, but that could be clearer.	Thank you for your comment we have reconstructed the sentence to improve clarity:<br><br>Round 2 of the survey was distributed via the emails provided by the participants during round 1.<br><br>The results highlight how many email addresses were left by participants:<br><br>Email addresses were provided by 365/368 participants from round 1.
1.2	Were any mechanisms in place to protect from hoax respondents to the surveys, particularly noting the chance to win a monetary voucher? See, for example, https://doi.org/10.2196/68092. Perhaps a comment can be added to the Strengths and Limitations section.	Thank you for your useful comment we had identified this as a problem and used a CAPTCHA system within REDCap to mitigate hoax respondents. We have modified the text to include a sentence in the methods section:<br><br>We have also added and provided a short summary about CAPTCHA using the reference you suggested:<br><br>To avoid hoax respondents, an increasingly common problem with online survey research, CAPTCHA screening software was used at the beginning of each survey. Captcha is a recognised method of protecting against bots.<br><br>References:

		Ng WZ, Erdembileg S, Liu JCJ, Tucker JD, Tan RKJ. Increasing Rigor in Online Health Surveys Through the Reduction of Fraudulent Data. J Med Internet Res. 2025;27:e68092.<br><br>And the specific reference for the software we used has been added for clarity:<br><br>Rezniczek GA. nedCAPTCHA (REDCap External Module) [Computer software]. Version 1.4.2 ed2025.
1.3	When were the various elements of the work undertaken?	Thank you for this comment, we have now included in the methods section of the article:<br><br>The research was conducted over a 2-year period between 2023 and 2025.
1.4	P10, line 52: please replace "1" with "One"	Thank you for this comment we have made this alteration:<br><br>One information item met the pre-defined criteria to be removed from the information set – the financial cost to the NHS of induction.
**Item**	**Reviewer 2 Comment**	**Author's Response**
2.1	In round 1, as many as 87% of participants in the parent group were White British, and the dominant participants were people aged 31–40 with bachelor/postgraduate education. This means that the final CIS may reflect, to a greater extent, the informational needs of women who are more educationally and socially privileged, and more digitally active, rather than the pregnant population as a whole. This is not really the ethnic diversity declared by the authors.	Thank you for your comment. Diversity in our sample is a very important point, and we tried to mitigate this in several ways, and throughout all stages of the research. Including, translations, recruiting online and in person, holding in person meetings in lower income areas of Liverpool. However, despite this, we were below the UK average diversity and therefore we have strengthened this comment in the limitations section.<br><br>A key strength of this study is its consensus methodology with parents and professionals which strengthened its essential clinical content and relevance, ensuring it is well situated to support discussions about birthing options.(18) However, despite our attempts to recruit a diverse sample of participants, we were not able to access a sample completely representative of the birthing population. 87% of participants were white British, 13% were other ethnicities, falling below the UK average ethnic minority rate 16%. 85% of parents had higher-level education, which is above the national reading age of 9-11. (43) With this in mind, the core information set could be more set up for the groups described above. On the other hand, the majority of participants were 31-40 years old, capturing the most common age group to give birth in the UK, according to the most recent national maternity data where 53% of births occur

		between the age of 30-39, 36% are age 20-29, with individuals at the extremes of age representing the smallest proportion.(3)<br><br>We adopted several strategies to increase the diversity of the participants - the Delphi surveys were online, and the involvement groups were held on Microsoft Teams, supplemented by in-person meetings in a local children's centre in a socioeconomically disadvantaged area of Liverpool, where we had a majority of either non-English speaking or black and minority ethnic group women. We aimed to capture the opinions of stakeholders from all over the UK, as well as ensuring women who do not usually engage in research had their voices included, particularly while populating the core information set. The survey, study documents and materials calling attention of potential participants to the research were made available in English, Arabic and Polish, the most spoken languages in study sites, with local efforts made to recruit non-English speakers. Nevertheless, no non-English speaking participants attempted the survey. This highlights a gap in engagement, one possible approach in future would be to meet women face to face with interpreting facilities available. Methods of seeking participants through several social media platforms and local hospitals aimed to capture participants across all educational backgrounds but factors including, internet-access, the survey length, usability and the Delphi study itself may have influenced the educational level of the respondents. (44)<br><br>References:<br><br>McNair AGK, Whistance RN, Main B, Forsythe R, Macefield R, Rees J, et al. Development of a core information set for colorectal cancer surgery: a consensus study. BMJ Open. 2019;9(11):e028623.<br><br>HS Document Readability Tool 2026 [Available from: https://readability.ncldata.dev/.<br><br>NHS Maternity Statistics, 2024-25 - NHS England Digital: @NHSDigital; 2026 [Available from: https://digital.nhs.uk/data-and-information/publications/statistical/nhs-maternity-statistics/2024-25.<br><br>R Profillidis VA, Botzoris GN. Chapter 4 - Executive Judgment, Delphi, Scenario Writing, and Survey Methods. In: Profillidis VA, Botzoris GN, editors.

		Modeling of Transport Demand: Elsevier; 2019. p. 125-61.eference:
2.2	The authors report that 58 professionals participated in round 1, but only 23 in round 2. This is a substantial drop, which may affect the stability of the consensus between groups. Simply stating that this level of attrition "is not unusual for a Delphi" does not fully solve the problem.	Thank you for your comment, we have elaborated on the limitation of attrition focusing on the parent professional split and general Delphi research.<br><br>Limitations of Delphi studies include difficulties with retention; attrition rates can vary from 0-92%. (23) The drop-out rate between rounds was 52% across respondents. Initial observations reveal that if the group is split into parents and professionals, the dropout rate is 50% among parents and 60% among professionals. Which would support the assumption that there an issue maintaining recruitment of professionals across rounds in comparison to parents. Furthermore, of the professionals that responded in the second round, 65% were also parents. While it was a 50/50 split in round one. This may indicate that the group who feel most strongly about having this information available are parents and that professionals have multiple competing demands on their time.(45)<br><br>The general guidance to mitigate dropout in Delphi studies, is to provide a short between-round time frame and send reminders out to participants. (46) This Delphi had a 2-week between round timeframe, and 3 reminders were sent out to round 1 survey participants via email with the aim of maintaining interest and ownership. Although the response rate falls within the normal bounds of survey research, it is noteworthy that, in a Delphi study, the sample size and heterogeneity of the sample can affect response rates between rounds. (46, 47). Thus, in an effort to be inclusive, this study required a larger sample size potentially contributing to the dropout rate.<br><br>References:<br><br>Shang Z. Use of Delphi in health sciences research: A narrative review. Medicine (Baltimore). 2023;102(7):e32829.<br><br>Peckham S, Zhang W, Eida T, Hashem F, Kendall S. Research engagement and research capacity building: a priority for healthcare organisations in the UK. Journal of Health Organization and Management. 2023;37(3):343-59.<br><br>Varndell W, Fry M, Lutze M, Elliott D. Use of the Delphi method to generate guidance in emergency nursing practice: A systematic review. Int Emerg Nurs. 2021;56:100867.

		Story DA, Tait AR. Survey Research. Anesthesiology. 2019;130(2):192-202.
2.3	One group included: people planning pregnancy, currently pregnant women, postpartum women, partners, and in addition professionals could also respond from the parent perspective. This raises the question whether "parent/non-professional" is really a homogenous analytical category?	Thank you for your comment we have clarified the group split in the methods section:<br><br>The groups were split into "professionals" and "parents". Those who indicated that they either worked in maternity care, were a medico-legal professional or from a representative group were categorised primarily into the "professional" group. Professionals were also able to state whether they were a parent and data for this has been provided in table 2.<br><br>We have also improved the wording in table 2 to make it clearer that the table has been analysed according to the participant's professional status and demographic data only was collected including their "parent status" but the professional group itself was not split when it came to the ranking of the information points.<br><br>The split has then been discussed in the discussion section in more detail, thank you for highlighting the importance of commenting on the homogeneity of the sample:<br><br>Professionals had the opportunity to indicate whether they were a parent. While this may increase the heterogeneity of this group, the professional status of the participant was considered their primary identity for the sake of the Delphi and feedback between rounds. That said, it was felt that the professional may wish to include their parent-status as they may feel that it is part of their core-identity.
2.4	The discussion and conclusions sometimes go one step too far, suggesting that standardization of the conversation by itself will reduce variation and improve access to information. This is probable, but it has not been demonstrated yet.	Thank you for your comment, we have modified the wording in the article:<br><br>This study has demonstrated a systematic way of standardising critical pieces of information that women, their partners and health professionals collaboratively feel are important to discuss prior to induction of labour to support their labour and birth decisions. Further work disseminating and utilising the information in a clinical setting will evaluate whether such a tool can reduce the variation in the information that women and their partners access while making their birth decisions.

2.5	The authors could consider supplementing the section on factors influencing induction success and the course of induction in women with an unfavourable cervix with additional observational data (doi:10.1155/2020/4985693). This study may be particularly useful in the context of information given to patients about the probability of failed induction and the importance of baseline cervical status.	We thank you for this important consideration, we have provided a populated core information set which has provided the information around the core information points using the predefined hierarchy of evidence – we appreciate your suggestion of the addition of this observational study and have added it to the reference list in the section "what to expect during an induction of labour" where factors influencing success are listed.<br><br>Reference:<br>Jakub M, Marta M, Jagoda G, Kamila G, Stanislaw G. Is Unfavourable Cervix prior to Labor Induction Risk for Adverse Obstetrical Outcome in Time of Universal Ripening Agents Usage? Single Center Retrospective Observational Study. J Pregnancy. 2020 Sep 1;2020:4985693. doi: 10.1155/2020/4985693. PMID: 32953176; PMCID: PMC7481947.
2.6	Reference numbers 22 and 26 appear twice in the reference list.	Thank you for highlighting this – we have deleted this duplicate.
2.7	The authors state that the final CIS content was populated according to a predefined hierarchy of sources, with priority given to NICE, RCOG, and systematic reviews. This is reasonable, but it has consequences: not all issues considered important by participants necessarily have equally strong evidene base. The text should more clearly distinguish:<br>(1) what was considered important by stakeholders,<br>(2) what is well supported by high-quality evidence,<br>(3) where evidence gaps still exist.	Thank you for highlighting this, populating the core information set has exposed significant gaps and quality of evidence available.<br><br>We have included a short entry highlighting this as a limitation of populating the sets. While the core information points have gained consensus to be included in the set, the evidence is not always robust enough to make strong statements in the populated sets, we also needed to ensure the information was accessible to all education levels and adjusted information and wording according to the feedback in engagement groups. The research and parent groups considered how to articulate the hierarchy of background evidence to the audience and felt a paragraph at the beginning of the set, and a reference list would provide transparency while maintaining accessibility:<br><br>**"This information is intended as a guide and uses evidence from national guidelines, national statistics and research studies. It includes some more general information that explains usual practice. It has the best available information at the time that it was made (2025)."**<br><br>The article includes a paragraph highlighting the need for further research and evidence available to populate the core information set. Which we have expanded to address the points you raise.

		Further research is required. During involvement meetings women suggested that local data is important for making birth decisions. However, that was beyond the scope of this study with its predefined hierarchy of evidence and commitment to use evidence underpinning NICE guidelines, systematic reviews and national statistics. That said, not all the evidence women wanted was available to populate the CIS. For example, while existing research shows induction of labour can reduce the likelihood of stillbirth, which disproportionately affects Black and Asian mothers, those with obesity and older women, there is no high quality, clear evidence to include information for women on optimal timing of induction in these groups. The hierarchy of literature used by the research team highlights that guidelines contain mixed quality evidence within them. However, when populating the sets, women in our engagement groups felt that communicating the strength of evidence would overcomplicate the information. Instead, they favoured transparency by including references to the sources used. Future research should address questions important to women, for example the optimal timing of induction of labour, and find ways to engage women from all groups in information development and use.

VERSION 2 - REVIEW

Reviewer **1**

Name **East, Christine**

Affiliation **La Trobe University, School of Nursing and Midwifery / Judith Lumley Centre**

Date **23-Apr-2026**

COI

Thank you for addressing the feedback.

Reviewer **2**

Name **Mlodawski, J**

Affiliation **Jan Kochanowski University in Kielce, Obstetrics and Gynecology**

Date **23-Apr-2026**

COI

I still have a few smaller comments:

they still generalise the meaning of the final set a bit too broadly. In the Discussion the authors themself honestly state that 87% of participants were White British, 85% of parents had higher education, and no participant used the Arabic or Polish version. Also, the dropout between rounds was 52% overall, around 50% among parents and 60% among professionals, and in round 2 as many as 65% of professionals were also parents. This does not invalidate the result, but it does mean that the final CIS is rooted most strongly in the perspective of relatively well-educated, English-speaking participants, and partly in a more parent-leaning professional group. so the final conclusion would be better if it was softened just a little bit.

there is still not fully clear enough separation between what stakeholders considered important, and what is strongly suported by high-quality evidence. The authors did improve this point and added limitations related to the evidence hierarchy, but in the abstract and conclusion it still sounds a bit too uniform.

In Table 1 the number of responses for roles exceeds N=310, and in Table 2 a simmilar issue appears for the parent/professional roles. this is probably because more than one category could be selected, but it is not fully obvious at first glance everywhere.

In different parts of the manuscript there are terms like “pregnant people, parents and professionals”, “women/birthing people”, and in the acknowledgements the authors add that they use women/mother as a collective term. the intention is understandable, but linguistically and editorially the text would be stronger if the terminology was unified one more time across the abstract, methods, discussion and final declarations.

VERSION 2 - AUTHOR RESPONSE

Reviewer	Reviewer’s comment	Author's response
Dr. Christine East, La Trobe University	Thank you for addressing the feedback.	We thank the reviewer for their contributions in R1.
Dr. J Mlodawski, Jan Kochanowski University in Kielce	I still have a few smaller comments:	We thank the reviewer for their further comments. We have responded to Reviewer 2’s specific remarks on a point-by-point basis and made the suggested changes as detailed below.

**Item**	**Reviewer 2 comment**	**Author's response**
1.1	They still generalise the meaning of the final set a bit too broadly. In the Discussion the authors themself honestly state that 87% of participants were White British, 85% of parents had higher education, and no participant used the Arabic or Polish version. Also, the dropout between rounds was 52% overall, around 50% among parents and 60% among professionals, and in round 2 as many as 65% of professionals were also parents. This does not invalidate the result, but it does mean that the final CIS is rooted most strongly in the perspective of relatively well-educated, English-speaking participants, and partly in a more parent-leaning professional group. so the final conclusion would be better if it was softened just a little bit.	Thank you for your comment we have re-written the conclusion in the main body and abstract see below:<br><br>Abstract:<br>This study has established a consensus-based CIS for induction of labour from a sample of the birthing population and staff providing their care. The resultant set has been populated with evidence in line with national guidelines. It can be used by women and clinicians as a standardised starting point from which to personalise discussions about birth.<br><br>Main body conclusion:<br>This study has established a core information set for induction of labour with a sample of the birthing population, their partners and healthcare professionals. This set acts as a standardised starting point from which clinicians can tailor information to support birth options. The set has been populated with the best available evidence in line with national guidelines. However, gaps in obstetric research evidence remain. Further work is required to disseminate and utilise the information in a clinical setting and engage women across diverse socio-economic and ethnic groups for personalised maternity care.
1.2	There is still not fully clear enough separation between what stakeholders considered important, and what is strongly supported by high-quality evidence. The authors did improve this point and added limitations related to the evidence hierarchy, but in the abstract and conclusion it still sounds a bit too uniform.	Thank you for your comment we have re-written the conclusion in the main body and abstract see below:<br><br>Abstract:<br>This study has established a consensus-based CIS for induction of labour from a sample of the birthing population and staff providing their care. The resultant set has been populated with evidence in line with national guidelines. It can be used by women and clinicians as a standardised starting point from which to personalise discussions about birth.<br><br>Main body:<br>This study has established a core information set for induction of labour with a sample of the birthing population, their partners and healthcare professionals. This set acts as a standardised starting point from which clinicians can tailor information to support birth options. The set has been populated with the best available evidence in line with national guidelines. However, gaps in obstetric research evidence remain. Further work is

		required to disseminate and utilise the information in a clinical setting and engage women across diverse socio-economic and ethnic groups for personalised maternity care.
1.3	In Table 1 the number of responses for roles exceeds N=310, and in Table 2 a similar issue appears for the parent/professional roles. this is probably because more than one category could be selected, but it is not fully obvious at first glance everywhere.	Thank you for highlighting this – as you've correctly identified, more than one category could be selected for some sections. We had put (able to select more than one) next to Role and Type of Birth but this was not consistent across both tables, so we have rectified this for consistency and in addition we have put a * next to these categories and at the end of the table written:<br><br>* Participants could select more than one category.
1.4	In different parts of the manuscript there are terms like "pregnant people, parents and professionals", "women/birthing people", and in the acknowledgements the authors add that they use women/mother as a collective term. the intention is understandable, but linguistically and editorially the text would be stronger if the terminology was unified one more time across the abstract, methods, discussion and final declarations.	We thank the reviewer for bringing this to our attention we have removed the words "women/birthing people" and used the term women and kept the sentence that acknowledges those that identify by other terms. See deletions in the tracked manuscript.
